# Interspersed Assembled Monolayers Enhance Hole Transport in High-Efficiency Organic and Perovskite Solar Cells

**DOI:** 10.1021/jacs.5c05341

**Published:** 2025-06-26

**Authors:** Chieh-Ming Hung, Jing-Han Shi, Hsiao-Chun Tsai, Chi-Ping Lin, Bo-Han Chen, Shang-Da Yang, Pi-Tai Chou

**Affiliations:** † Department of Chemistry, Center for Emerging Materials and Advanced Devices, 33561 National Taiwan University, Taipei 106319, Taiwan; ‡ Institute of Photonics Technologies, 34881 National Tsing Hua University, Hsinchu 300044, Taiwan

## Abstract

We propose a novel concept called interspersed assembled monolayers (IAMs), which leverage a dispersant molecule sharing a similar backbone with the host self-assembled monolayer (SAM) but possessing a distinct donor–acceptor (D–A) strength, aimed to suppress micelle formation. We designed two dispersant backbones, NNN (triazolo) and NSN (thiadiazolo), both featuring electron-withdrawing backbones, but NSN exhibits a substantially larger dipole moment, which in the current study seems to reduce interfacial energy barriers. Compared to SAMs, employing an IAM strategy with a long side chain (BO) raises power conversion efficiencies (PCE) across various organic solar cell (OSC) architectures. In the PM6:Y6 system, the original PCE of 16.46% improves to 16.72% when using NNN-BO, and further increases to 18.04% with NSN-BO, which has a stronger dipole moment. Perovskite solar cells (PSCs) also benefit, with PCE rising from 23.84 to 24.17% (NNN-BO) and 25.01% (NSN-BO). Moreover, short-side-chain variants NSN-C4 and NSN-IB in PM6:L8-BO-based OSCs yield PCE of 19.01 and 18.94%, respectively, while in PSCs, these dispersants achieve 24.95 and 24.94%, which all closely approximate the performance of long-side-chain NSN-BO (19.23 and 25.01%). Systematic investigation thus demonstrates that, in the design of IAM molecules, both the conjugated backbone and appended side chains must be taken into account. The underlying mechanisms have been revealed through comprehensive femtosecond transient absorption and time-resolved photoluminescence, showing the key to dispersants in promoting charge extraction, mitigating recombination and film morphology. These IAM-integrated components also exhibit environmental and thermal stability, paving a practical way to high-performance IAM-based PSCs and OSCs.

## Graphical abstract

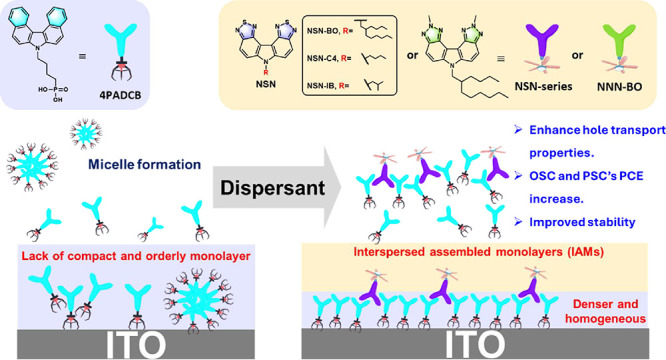

## Introduction

Recently, p-type self-assembled monolayers (SAMs) have been extensively employed in both organic solar cells (OSCs) and perovskite solar cells (PSCs).^1–5^ These ultrathin layers have attracted considerable attention primarily because the molecular dipole inherent to SAMs can effectively modify the work function of indium tin oxide (ITO).^6–9^ Such an adjustment aligns the energy levels between the highest occupied molecular orbital (HOMO) of the polymer donor and the valence band of the perovskite, resulting in a cascade-like band structure. This arrangement lowers the energy barrier for charge extraction and enhances carrier transport. Furthermore, utilizing a SAM as an extremely thin interfacial layer can simultaneously reduce the device’s series resistance and refine the electrode surface morphology. Compared with conventional materials such as PEDOT:PSS, whose acidity deteriorates long-term device durability and PTAA which can reduce reproducibility during synthesis, SAM-based approaches offer better operational stability and reliability. Thanks to these advantages, the implementation of SAMs as hole transport layers has successfully elevated the PCE of OSCs to over 20% ^10–14^ and pushed that of PSCs beyond 26%.^15–22^ This achievement underscores the substantial potential of SAMs in next-generation solar cell technologies demanding both high efficiency and sustained performance.

Most SAMs must be dissolved in highly polar solvents, which causes the hydrophobic tails of the molecules to cluster and form micelles, hindering the formation of a compact and orderly monolayer on the substrate. To address this, the use of a cosolvent has been proposed to adjust overall solubility and solvent polarity, thereby preventing spontaneous aggregation or micelle formation of SAM in solution and maintaining as many molecules as possible in a monomeric state.^23–25^ This strategy ultimately facilitates the creation of a more uniform and well-organized SAM on the substrate. Employing a co-SAM—formed from two or more types of SAM molecules—can further diminish the packing constraints posed by using only a single molecular species.^26–31^ When a single type of molecule is used exclusively, factors such as molecular orientation, length compatibility, and surface affinity can lead to local defects or incomplete coverage. Introducing molecules with different sizes or functional groups allows for mutual compensation, filling any gaps that may arise in a monomolecular layer and thus yielding a denser, more homogeneous film. During the solution processing and film formation, different molecules also affect one another’s affinity for both the solvent and the substrate. If one molecule adsorbs first and modifies the local energy landscape of the substrate, the second molecule can more readily insert or attach nearby. Notably, the presence of aromatic rings and other structural motifs can reinforce the overall film morphology via hydrogen bonding or push–pull interactions, reducing defects such as cracks or delamination caused by thermal or mechanical stresses. In brief, the co-SAM strategy may simultaneously achieve (1) synergistic functionality, (2) enhanced coverage and compactness, and (3) improved film formation kinetics and stability. As a result, problematic issues like interfacial leakage current and carrier recombination can be suppressed.

In this work, we merge the concepts of co-SAM with dispersant molecules, coining the term “interspersed assembled monolayers” (IAMs). Specifically, we selected 4PADCB—widely employed in both OSCs and PSCs—as the host SAM, and designed two kinds of dispersants, termed NNN and NSN (see Scheme 1 for the structures of 4PADCB, NNN, and NSN), whose hydrophobic backbones resemble that of 4PADCB. Unlike conventional co-SAM strategies, NNN and NSN do not contain a hydrophilic terminus but instead feature a 2-butyloctyl (BO) substituent, namely NNN-BO and NSN-BO. In addition, we synthesized NSN-C4 and NSN-IB featuring shorter side chains to systematically evaluate whether these modifications would diminish their effectiveness as IAMs agents. We expect that the newly designed series of molecules may accomplish several main objectives: (1) The structural similarity between NNN/NSN and 4PADCB reduces micelles through intermolecular push–pull (donor–acceptor) interactions. Simultaneously, dispersants bearing longer side chains provide greater steric hindrance than those with shorter side chains, thereby enhancing IAM dispersion and strengthening their ability to disrupt 4PADCB micellar aggregates. The phenomenon is referred to as IAMs. The cartoon schematic illustrating this concept can be found in Scheme 1. (2) NNN and NSN possess higher dipole moments, thereby further boosting the hole-transport capability of 4PADCB. (3) NNN and NSN can infiltrate the organic film when the OSC active layer is deposited to facilitate long-range molecular ordering, benefiting the carrier extraction. (4) A denser SAM layer raises the contact angle for the perovskite precursor solution, leading to the growth of larger perovskite grains. For the above reasons, an IAM constructed on the 4PADCB backbone cannot both inhibit micelle formation and enhance the hole-extraction ability of the SAM.

**Scheme 1:**
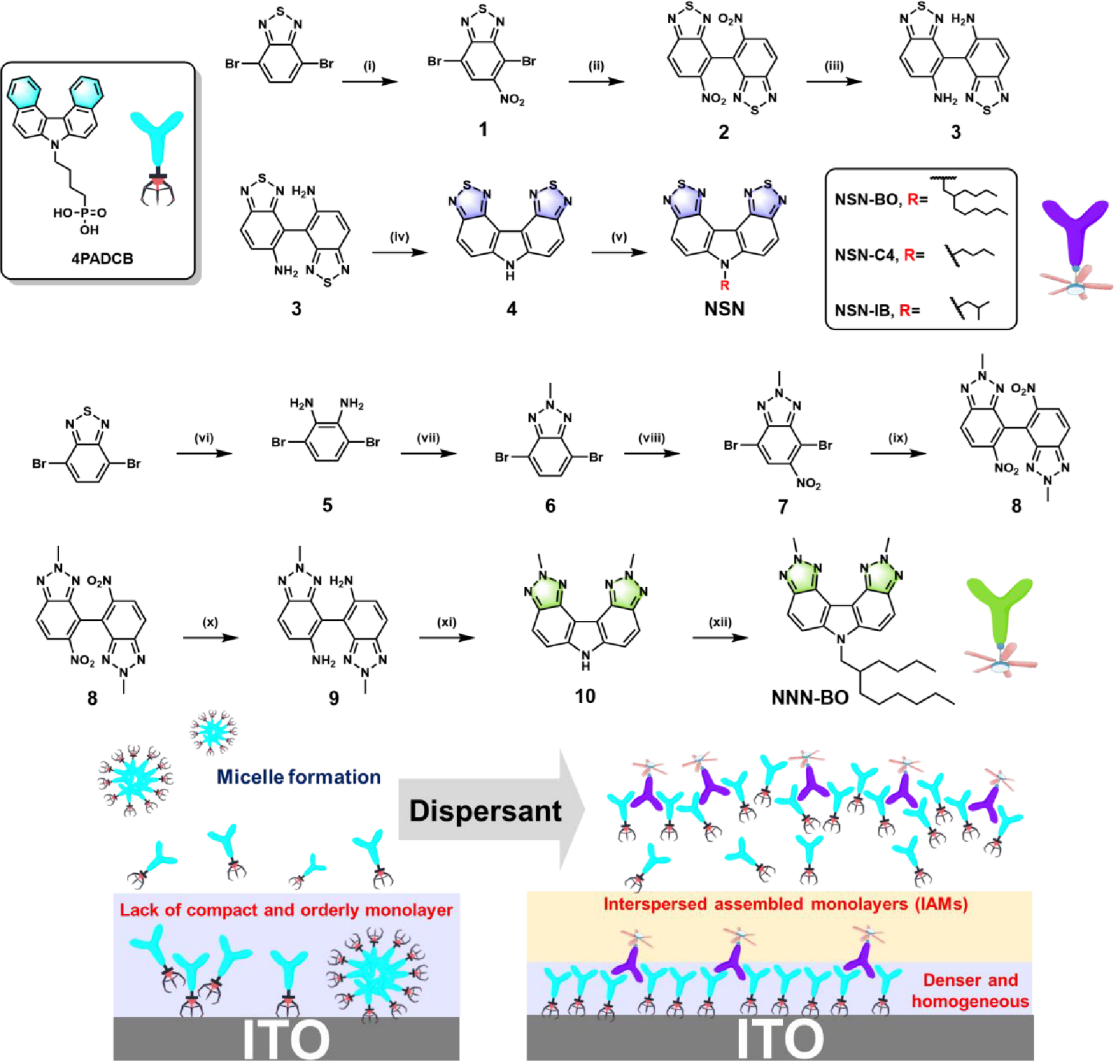
Synthetic Route to NNN-BO and NSN-Series^a^

a (i) Fuming HNO_3_, TfOH, 0 °C, 4 h; (ii) Cu powder, DMF, reflux, o/n; (iii) Fe, AcOH, R.T., 2 h; (iv) H_3_PO_4_, 120 °C, 2 h; (v) NaH, DMF, 60 °C, 1 h then alkyl halide, 120 °C, 18 h. The synthetic route to NNN-BO: (vi) NaBH_4_, EtOH, 0 °C, 6 h; (vii) NaNO_2_/H_2_O, AcOH, 0 °C→R.T. then MeI, K_2_CO_3_, DMF, 65 °C, o/n; (viii) fuming HNO_3_, TfOH, 0 °C, 4 h; (ix) Cu powder, DMF, reflux, 2 days; (x) Fe, AcOH, R.T., 2 h; (xi) H_3_PO_4_, 120 °C, 2 h; (xii) NaH, DMF, 60 °C, 1 h then 5-(iodomathyl)­undecane, 120 °C, 18 h. Cartoon schematic illustrating how a pure SAM tends to form micelles, leading to a loosely packed hole selective layer (HSL), whereas the introduction of dispersants alongside the host SAM facilitates the formation of interspersed assembled monolayers (IAMs), resulting in a more compact and efficient HSL.

Through the optimized IAMs formulation (4PADCB:dispersant = 10:1, dissolved in ethanol (EtOH):chloroform­(CF) = 4:1, with a total concentration of 1.1 mg/mL for OSCs and 0.55 mg/mL for PSCs), a well-controlled interface modification is achieved, For the PM6:L8-BO OCS, in comparison to the reference device using pure 4PADCB as SAMs to achieve a PCE of 18.12%, incorporating 4PADCB + NSN-BO leads to a pronounced rise in both short-circuit current (*J*_SC_) and fill factor (FF), elevating the PCE up to 19.23%. For perovskite solar cells, PCE is boosted from 23.84, 24.17 to 25.01% incorporating SAMs from pure 4PADCB, 4PADCB + NNN-BO to 4PADCB + NSN-BO, respectively. In organic and perovskite solar cells, using NSN-C4 and NSN-IB with shorter side chains as the IAM strategy yields only slightly lower efficiencies compared to NSN-BO. Consequently, we deduce that prioritizing modifications to the molecular backbone, rather than altering side chains, is more effective for enhancing overall device performance. It is important to emphasize that the choice of side chain is not trivial: side-chain steric bulk modulates the dispersion of the IAM within the host-SAM matrix and thus directly impacts hole selective layer (HSL) morphology. Equally important is the fact that IAMs significantly enhance environmental and thermal stability. Details of the results and discussion, particularly the mechanistic studies by comprehensive time-resolved spectroscopy, are elaborated below.

## Results and Discussion

The synthetic routes to NSN and NNN-BO are depicted in Scheme 1 where synthetic detail and corresponding characterizations are elaborated in the Supporting Information (SI). Briefly, NSN was synthesized via the nitration of 4,7-dibromobenzo­[*c*]­[1,2,5]­thiadiazole, followed by Ullmann reaction to afford compound 2. Notably, using an excess of copper powder and extending the reaction time streamlines both homocoupling and dehalogenation in a one-pot fashion with moderate yield (50–60%). Subsequently, the nitro groups were reduced to amines with iron dust and followed by a cyclization in phosphoric acid. The carbazole then underwent a nucleophilic substitution reaction with different alkyl halides, yielding NSN-BO, NSN-C4, and NSN-IB respectively. For NNN-BO, the same starting material was subjected to reduction by sodium borohydride. A sequential oxidative cyclization with sodium nitrite and methylation with iodomethane afforded compound 6, then the remaining steps were identical to those for NSN. Single crystals of NSN-BO were successfully obtained using a dichloromethane/hexane cosolvent system. However, the crystallization of NNN-BO was unsuccessful despite numerous attempts. Since the long-branched alkyl chain enhances solubility in nonpolar solvents, the crystallization would rely on the intermolecular interactions between the carbazole-derived head groups. We believe that NSN boasts a rather high polarity (6.22–6.46 D) that helps facilitate the crystallization in the presence of the antisolvent (hexane), whereas the lower polarity of NNN-BO (1.14 D) does not favor this process. Therefore, the crystal structure of the analogous compound 10 is presented in Figure 1. Notably, the chromophore core of both compound 10 and NSN-BO exhibits a planar structure with a dihedral angle of <5°, indicating their structural similarity to 4PADCB (see Scheme 1). We also investigated various alkyl chains, with shorter branches or linear alkyl groups (e.g., methyl), and whether that will influence dispersant ability. Since the absence of or too small an alkyl group leads to insolubility in CF, the side chain was extended to four carbons. With the rather high reaction yield and the molecular dipole moments, we take the spotlight on NSN-BO.

**Figure 1:**
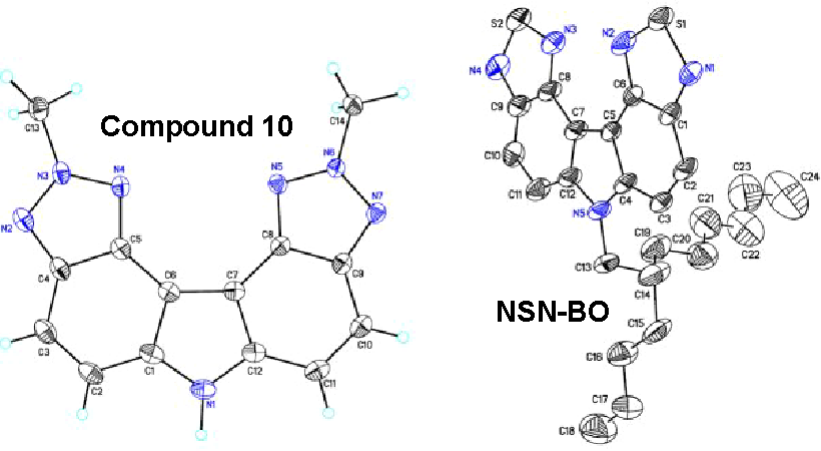
Crystal structure of compound 10 and NSN-BO.

To gain understanding of physical properties of NNN-BO and NSN-BO, we started out from the density functional theory (DFT) calculations at the B3LYP/6–31G (d, p) level to elucidate the molecular properties. As shown in Figure 2a, the gas-phase molecular dipole moments of NNN-BO and NSN-BO are calculated to be 1.14 and 6.46 D, respectively, where the dipole moment of NSN-BO surpasses that of 4PADCB (2.60 D; see Figure S1). Hence, introducing NSN-BO dispersants into 4PADCB, in theory, is able to enhance hole-extraction capability. Nonetheless, if the HOMO and lowest unoccupied molecular orbital (LUMO) levels of NNN-BO and NSN-BO fail to align with those of 4PADCB, no improvement in hole transfer would be realized. Fortunately, cyclic voltammetry (CV) analysis (Figure S2) reveals that NNN-BO and NSN-BO exhibit HOMO levels of −5.63 and −5.84 eV, respectively. Coupling these findings with their absorption spectra (Figure S3), the LUMO levels of NNN-BO and NSN-BO are calculated to be −2.27 and −2.81 eV, both of which form an appropriate cascade with 4PADCB (HOMO −5.35 and LUMO −2.28).^32^ Next, we deposited a 10:1 (4PADCB:dispersant) mixture, dissolved in an EtOH/CF blend (4:1 volume ratio), onto ITO substrates and conducted ultraviolet photoelectron spectroscopy (UPS) measurements. As displayed in Figure 2b,c, ITO modified by pure 4PADCB or 4PADCB + NNN-BO exhibits the same valence band (VB) energy of −5.36 eV, whereas incorporating 4PADCB + NSN-BO shifts the VB to −5.41 eV. This result indicates that when the dispersant has a sufficiently large dipole moment and a deep HOMO level, it can further modulate VB established by the host SAM. Similarly, Figures 2d and S1 show electrostatic surface potential (ESP) maps from the DFT calculations. The negative potential regions associated with the triazolo ring in NNN-BO and the thiadiazolo ring in NSN-BO can readily engage in donor–acceptor interactions with the positively charged carbazole segment of 4PADCB, thereby mitigating micelle formation through intermolecular push–pull effects (see Figure S4 for the schematic illustration). Moreover, by virtue of the push–pull effect and the vectorial addition of dipole moments, when the dipole moment of the IAM exceeds that of the host-SAM, the resulting overall dipole moment is enhanced, yielding a deeper valence-band maximum. This mechanism thus accounts for the observed valence-band level of −5.41 eV in the 4PADCB + NSN-BO system. Figure S5 presents X-ray photoelectron spectroscopy (XPS) analyses probing whether IAM incorporation reduces 4PADCB aggregation via push–pull interactions. The C 1s spectra reveal a clear π–π stacking signal at 290.88 eV for pure 4PADCB. Upon NNN-BO addition, this feature red-shifts to 290.71 eV; with NSN-BO, it red-shifts further to 289.78 eV, and its associated peak area diminishes significantly, confirming that IAM disrupts 4PADCB micelles through push–pull interactions. We then verified this hypothesis (donor–acceptor interactions) by dynamic light scattering (DLS) analysis (Figure 2e). The particle size distribution of pure 4PADCB dissolved in pure EtOH (1 mg/mL) is approximately 94.7 nm. This value aligns with previous reports of an 83.66 nm particle size distribution for 4PADCB micelles at 1 mg mL^–1^ in IPA, confirming that 4PADCB also forms micelles in EtOH.^25^ By employing a cosolvent system (EtOH:CF = 4:1, 1 mg/mL), the particle size decreases to 81.9 nm, indicating that the cosolvent approach partially inhibits micelle formation. Notably, blends of 4PADCB with dispersants NNN-BO or NSN-BO (EtOH:CF = 4:1, 4PADCB: dispersant = 10:1, total concentration = 1.1 mg/mL) exhibit substantially smaller particle sizes of 56.1 and 21.8 nm, respectively. DLS measurements confirm well-defined particle size distributions, validating the successful formation of IAMs and the suppression of aggregation. These findings further highlight the crucial role of dispersants in enhancing the stability and homogeneity of molecular assemblies. Water contact angle measurements were subsequently performed on ITO/4PADCB, both with and without dispersants. Figure 2f reveals that pure 4PADCB has the lowest contact angle of 35.78°, which increases to 38.08° upon incorporating NNN-BO and rises sharply to 44.79° with NSN-BO. These findings confirm that introducing dispersants—featuring structural similarity to 4PADCB along with stronger dipole moments and electron-withdrawing functionalities—favors tighter molecular packing in the SAM, culminating in a denser HSL.

**Figure 2:**
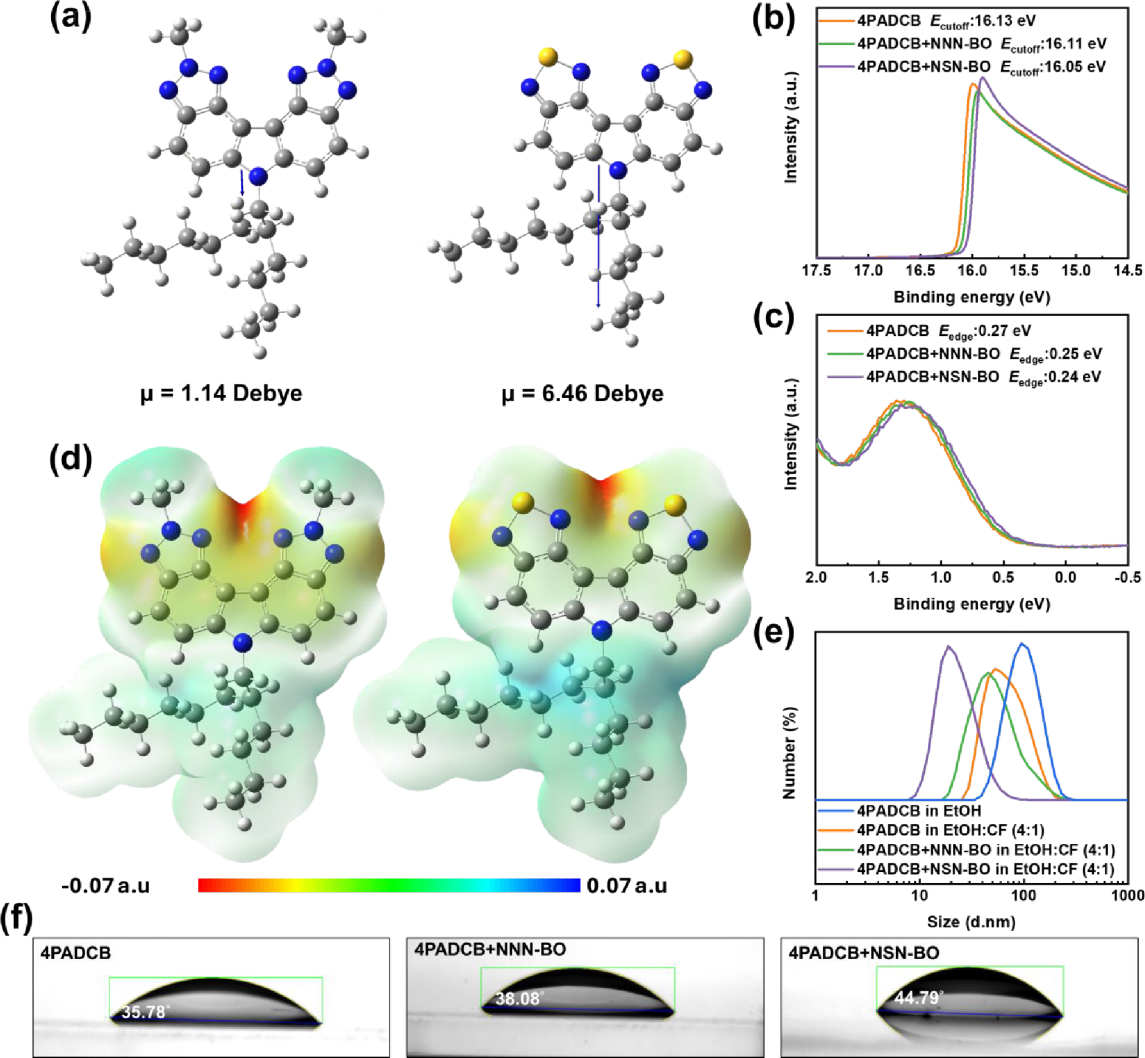
(a) Schematic illustration of the dipole moment vectors in the dispersants NNN-BO and NSN-BO. UPS results for (b) *E*_cutoff_ and (c) *E*_edge_ on ITO surfaces modified with SAMs, used to calculate the work function (WF) via the equation |21.22–(*E*_cutoff_–*E*_edge_)| = WF. (d) ESP distributions of the NNN-BO and NSN-BO molecules. (e) DLS data for 4PADCB (pure or cosolvent) in the presence or absence of dispersants. (f) Water-contact-angle measurements for ITO/4PADCB with and without dispersant-modified SAM layers.

From the above results, we have demonstrated that, as to the main backbone structure, NSN shows a more pronounced advantage than NNN. Accordingly, we next investigated the impact of variations in its side chains. Figure S1 illustrates that the dipole moments of NSN-C4 and NSN-IB are 6.40 and 6.22 D, respectively. From CV and absorption spectroscopy (Figures S2 and S3), the HOMO and LUMO levels of NSN-C4 are determined to be −5.78 and–2.88 eV, while those of NSN-IB are −5.81 and −2.86 eV. In addition, DLS results (Figure S6) indicate particle sizes of 28.0 nm for NSN-C4 and 28.3 nm for NSN-IB, revealing that shorter side chains alter the dispersion behavior of IAM molecules, which may in turn influence the performance of the resulting photovoltaic devices.

### Organic Solar Cells

In the study of OSCs experiments, we adopted the device architecture: MgF_2_/Glass/ITO/SAM or IAM (4PADCB, with or without dispersant)/Active Layer/PDINN/Ag (120 nm). An energy-level diagram is provided in Figure 3b, and the well-known PM6:Y6 served as the active layer (see Figure 3a for chemical structures). The champion *J–V* curves are shown in Figure 3c. In the absence of a dispersant (but with 4PADCB SAMs), the device exhibits an open-circuit voltage (*V*_OC_) of 0.862 V, a *J*_SC_ of 26.82 mA cm^–2^, and FF of 71.20%, yielding a PCE of 16.46%. When incorporating the dispersant NNN-BO, *V*_OC_ and *J*_SC_ remain nearly unchanged, but the FF increases to 72.28%, thus enhancing the PCE to 16.72%. Impressively, introducing NSN-BO preserves *V*_OC_ at 0.862 V yet substantially elevates *J*_SC_ and FF to 27.73 mA cm^–2^ and 75.47%, respectively, boosting the PCE up to 18.04%. The incorporation of IAM in the OSCs (PM6:Y6) leads to a substantial performance enhancement. We attribute this improvement to NNN-BO and NSN-BO effectively suppressing the formation of large micelles, thereby enabling 4PADCB to anchor as a valid hole-selective layer on the ITO surface. This mitigates interfacial charge accumulation and yields an increased FF. Moreover, the larger dipole moment of NSN-BO further facilitates charge extraction by 4PADCB, resulting in an enhanced *J*_SC_. As illustrated in Figure 3d, the incident photon-to-current conversion efficiency (IPCE) spectra of pristine 4PADCB and 4PADCB + NNN-BO are almost identical, both integrating to 24.9 mA cm^–2^. However, with the introduction of NSN-BO, the IPCE clearly improves across the entire spectral range, increasing the integrated current up to 25.7 mA cm^–2^. Similar results were observed in other high-performance active layers, where the PCE of D18:Y6 increased from 17.18 to 18.26%, while that of PM6:L8-BO improved from 18.12% to 19.23% (see Figure 3e, f). Table 1 summarizes these device parameters.

**Figure 3:**
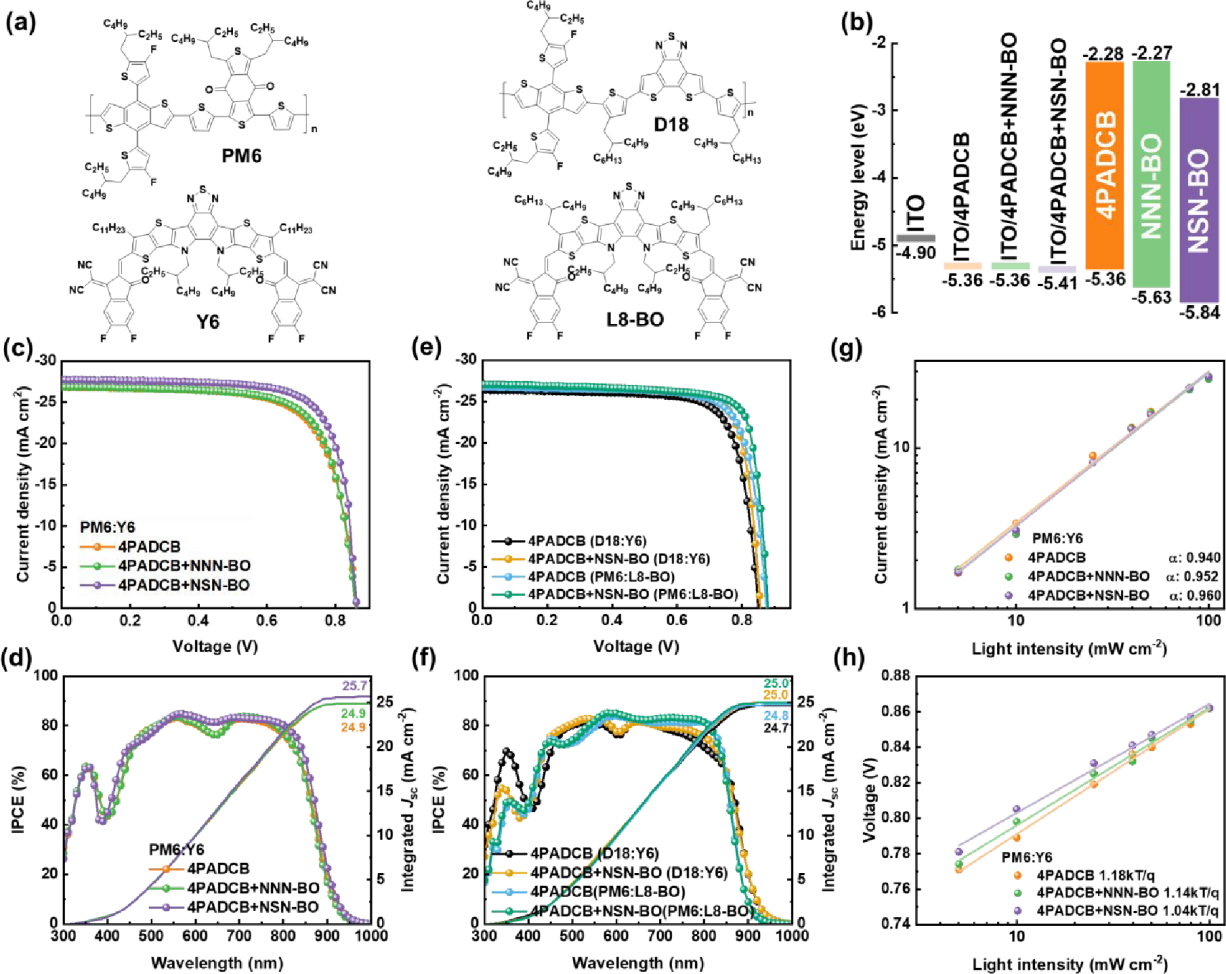
(a) Chemical structures of PM6, D18 polymer and Y6, L8-BO nonfullerene acceptor. (b) Schematic energy level diagram of different compositions in OSCs (c, e) *J*–*V* curves and (d, f) IPCE spectra of the PM6:Y6, D18:Y6 and PM6:L8-BO OSCs. Plots of (g) *J*_SC_ and (h) *V*_OC_ vs light intensities of the PM6:Y6 devices.

**Table 1:** Photovoltaic Parameters of Champion OSCs

	*V*_OC_ [V]	*J*_SC_ [mA/cm^2^]	FF [%]	PCE [%]
4PADCB^a^	0.862	26.82	71.20	16.46
4PADCB + NNN-BO^a^	0.862	26.83	72.28	16.72
4PADCB + NSN-BO^a^	0.862	27.73	75.47	18.04
4PADCB^b^	0.852	26.34	76.54	17.18
4PADCB + NSN-BO^b^	0.854	26.89	79.53	18.26
4PADCB^c^	0.880	26.74	77.05	18.12
4PADCB + NSN-BO^c^	0.882	27.07	80.52	19.23

a PM6:Y6.

b D18:Y6.

c PM6:L8-BO.

When shorter side-chain variants (4PADCB + NSN-C4 and 4PADCB + NSN-IB) were employed with PM6:L8-BO as the active layer, their corresponding device PCE were 19.01 and 18.94%, slightly below the 19.23% achieved by 4PADCB + NSN-BO (see Figure S7). Specifically, devices using NSN-C4 and NSN-IB both achieve a *V*_OC_ of 0.892 V, with *J*_SC_ values of 27.11 and 27.08 mA cm^–2^, respectively. The most pronounced difference lies in the fill factor of 78.60% for NSN-C4 and 78.40% for NSN-IB, which we ascribe to the reduced steric hindrance of their shorter side chains. This weaker-steric bulk diminishes the dispersion capability of the IAM molecules (see DLS results in Figure S6), thereby slightly impairing the uniform anchoring of 4PADCB. Nevertheless, NSN-C4 and NSN-IB still perform comparably to NSN-BO in PM6:L8-BO systems. These findings underscore that, beyond backbone engineering, side-chain selection must be carefully optimized in future IAM designs.

We then carefully investigated the dependence of the *J*_SC_ and *V*_OC_ on incident light intensity to elucidate the PM6:Y6 recombination dynamics. When the slope of *J*_SC_ versus light intensity approaches 1, the probability of bimolecular recombination is minimized. On the other hand, if the slope of *V*_OC_ versus light intensity is close to kT/q, then bimolecular recombination dominates. A significant deviation from this value (often exceeding 1.5 kT/q) points to enhanced trap-assisted recombination.^33–36^ From Figure 3g, the slopes for pure 4PADCB, 4PADCB + NNN-BO, and 4PADCB + NSN-BO are calculated to be 0.940, 0.952, and 0.960, respectively, indicating that the incorporation of dispersants further suppresses bimolecular recombination. Meanwhile, Figure 3h shows the slope of the plot for *V*_OC_ versus light intensity to be 1.18, 1.14, and 1.04 kT/q, respectively, demonstrating that NSN-BO more effectively mitigates trap-assisted recombination, thereby contributing to an increased FF. Transient photovoltage (TPV) and Transient photocurrent (TPC) together provide direct insight into trap-mediated recombination and the effective carrier lifetimes under device operation. As shown in Figure S8, the inclusion of IAM additives markedly extends the TPV-measured carrier lifetime from 5.47 μs for 4PADCB alone to 10.11 μs with 4PADCB + NNN-BO and 12.90 μs with 4PADCB + NSN-BO. This increase indicates that IAMs suppress electron–hole recombination in the bulk, thereby prolonging free-carrier survival. Concurrently, TPC lifetimes decrease from 0.69 μs (4PADCB) to 0.45 μs (NNN-BO) and 0.42 μs (NSN-BO), demonstrating that IAM incorporation effectively reduces interfacial trap densities and accelerates carrier extraction to the electrodes.

Based on the light-intensity dependence of *V*_OC_, TPC, and TPV, we infer that an optimized interface could also reduce defects within the bulk heterojunction (BHJ) in addition to the improvement of SAMs property. To test this hypothesis, we used Grazing-Incidence Wide-Angle X-ray Scattering (GIWAXS) to examine potential structural changes in the active layer caused by SAM modification. The 2D GIWAXS patterns in Figure S9 show that SAMs modified with NSN-BO or NNN-BO drive the (010) π–π diffraction peak of PM6:Y6 to higher *q*_*z*_ values. Subsequent integration in the *q*_*z*_ and *q*_*xy*_ directions (see Figure S10) reveals that, compared with pristine 4PADCB ((010) *d*-spacing = 3.66 Å), the *d*-spacing is reduced to 3.63 and 3.60 Å upon introducing NNN-BO and NSN-BO, respectively. Also, the crystal coherence length (CCL) expands from 18.72 Å (4PADCB) to 18.78 Å (4PADCB + NNN-BO) and 19.21 Å (4PADCB + NSN-BO). Additionally, the (100) *d*-spacing increases from 21.58 Å (4PADCB) to 21.80 Å (4PADCB + NNN-BO) and 21.88 Å (4PADCB + NSN-BO). NNN-BO and NSN-BO may also partially diffuse into the organic layer, thereby helping the BHJ improve the long-range ordering. This enhanced structural organization facilitates more efficient carrier extraction and transport.^37–41^ A summary of the GIWAXS results is provided in Table S1.

We then employ femtosecond transient absorption spectroscopy (fs-TAS) to examine how SAMs modified by dispersants influence carrier dynamics in the active layer. In this approach, the sample architecture consists of MgF_2_/glass/ITO/SAM or IAM/PM6:Y6, with photoexcitation (850–1000 nm) from the glass side and probing in the 600–1550 nm range. As shown in Figure 4a–c, the spectra exhibit similar characteristic signals. The negative signal at 600–650 nm corresponds to ground-state bleaching (GSB) of PM6, while from approximately 1 ps onward, the positive signal at 670–750 nm represents charge separation (CS) in the PM6:Y6 blend. The negative signal spanning 700–850 nm arises from GSB of Y6, whereas the positive signal at 850–1000 nm is attributed to the localized exciton (LE) state of Y6. Finally, the positive feature near 1500 nm is linked to the delocalized single exciton (DSE) of Y6.^42–47^ As depicted in Figure S11, curve fitting of the Y6 LE signal yields lifetimes of 67.4, 119.8, and 140.6 ps for 4PADCB, 4PADCB + NNN-BO, and 4PADCB + NSN-BO, respectively. A longer LE lifetime can be ascribed to a more highly ordered BHJ structure, which reduces bimolecular recombination and increases the LE population. A similar trend is observed for the DSE lifetime (4.7, 5.3, and 5.8 ps, respectively see Figure S12). By contrast, the Y6 GSB lifetimes trend in the opposite direction—35.4 ps (pristine), 27.7 ps (NNN-BO), and 27.4 ps (NSN-BO), as shown in Figure 4d. This decay primarily reflects hole transfer from Y6 to PM6, where a faster decay suggests a higher hole-transfer efficiency, thereby reducing the geminate recombination. Further insights are provided by the fitting of PM6 GSB kinetics (Figure 4e), which reveal both rise and decay time constants. Consistent with the Y6 GSB findings, the rise and decay times are 70.5 and 506.9 ps for pure 4PADCB, 49.6 and 443.1 ps for 4PADCB + NNN-BO, and 13.2 and 355.6 ps for 4PADCB + NSN-BO. Shorter rise times mean holes are more efficiently received by PM6 within the BHJ, while faster decay indicates faster transfer of holes to the HSL, thereby reducing nongeminate recombination. Lastly, Figure 4f shows the fitting results of the CS signals, which follow a trend consistent with the previous analyses. For 4PADCB, the rise and decay times are 5.3 and 513.2 ps, respectively. In 4PADCB + NNN-BO, these intervals become 5.1 and 583.0 ps, whereas in 4PADCB + NSN-BO, the rise shortens further to 3.8 ps, and the decay extends to 825.6 ps. A shorter rise time indicates enhanced exciton dissociation efficiency in the BHJ, implying that the interaction between NSN-BO and 4PADCB effectively facilitates exciton splitting within the BHJ. A longer decay suggests the generation of more free carriers, thereby contributing to increased *J*_SC_ in OSCs. In summary, the fs-TAS results confirm that NSN-BO not only inhibits micelle formation and enhances hole extraction, it also penetrates the active layer to improve crystal *d*-spacing and CCL, resulting in beneficial effects on exciton and charge separation processes.

**Figure 4:**
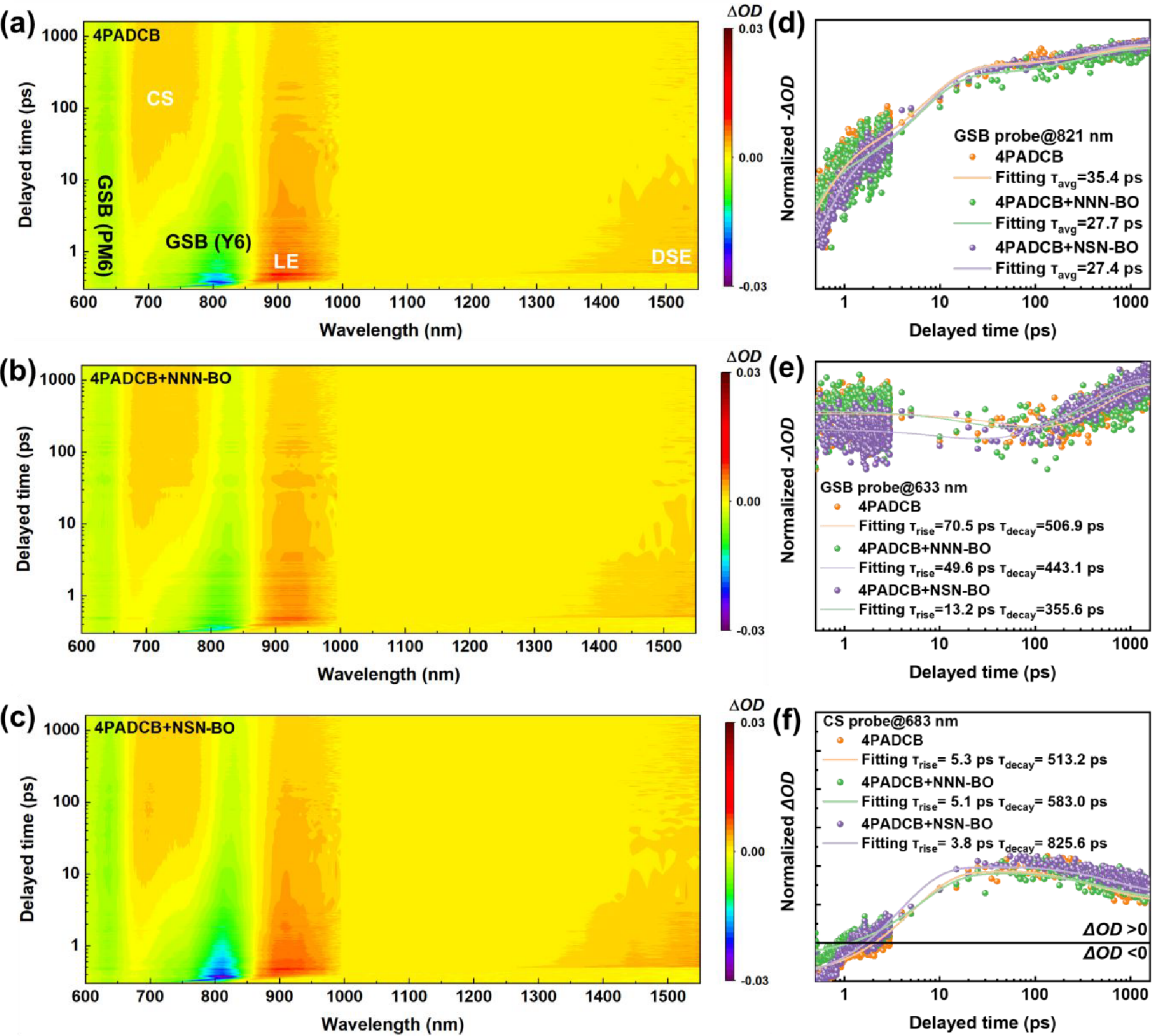
(a–c) 2D fs-TAS plots of the ITO/4PADCB with or without dispersants/PM6:Y6. The rise and decay lifetime of ITO/4PADCB with or without dispersants/PM6:Y6 films under GSB signal (d) at 821 nm for and (e) at 633 nm and CS signal at (f) 683 nm.

### Perovskite Solar Cells

The dispersant-modified SAMs are also highly beneficial for PSCs. The water contact angle results (Figure 2f) lead us to speculate that the dispersant may promote the formation of larger perovskite grain sizes.^25,48,49^ In this study, the perovskite films were deposited onto the SAM-modified substrates and examined by scanning electron microscopy (SEM) in plan-view mode to verify this hypothesis. As shown in Figure 5a, the perovskite film on unmodified 4PADCB appears relatively rough. It contains tiny pinholes, along with and loosely formed grain boundaries, features that increase nonradiative recombination (*k*_nr_), thereby reducing *V*_OC_. In comparison, as shown in Figure 5b, the perovskite film on 4PADCB + NNN-BO exhibits a smoother morphology and almost no pinholes. Furthermore, Figure 5c shows that films deposited on 4PADCB + NSN-BO obtained larger grain sizes, indicating more effective suppression of *k*_nr_.^50–54^ Although 4PADCB + NSN-BO films still exhibit a small number of pinholes, these surface defects can be repaired by a passivation layer. However, defects within the bulk of the perovskite rely on the intrinsic film formation process for mitigation. SEM cross-sectional analysis (see Figure S13) reveals that pure 4PADCB films contain numerous internal grain boundaries, which are prone to bimolecular recombination. In contrast, IAM-processed perovskite films exhibit a more continuous and densely packed grain structure, thus contributing to an extended carrier lifetime and, consequently, enhanced device performance.

**Figure 5:**
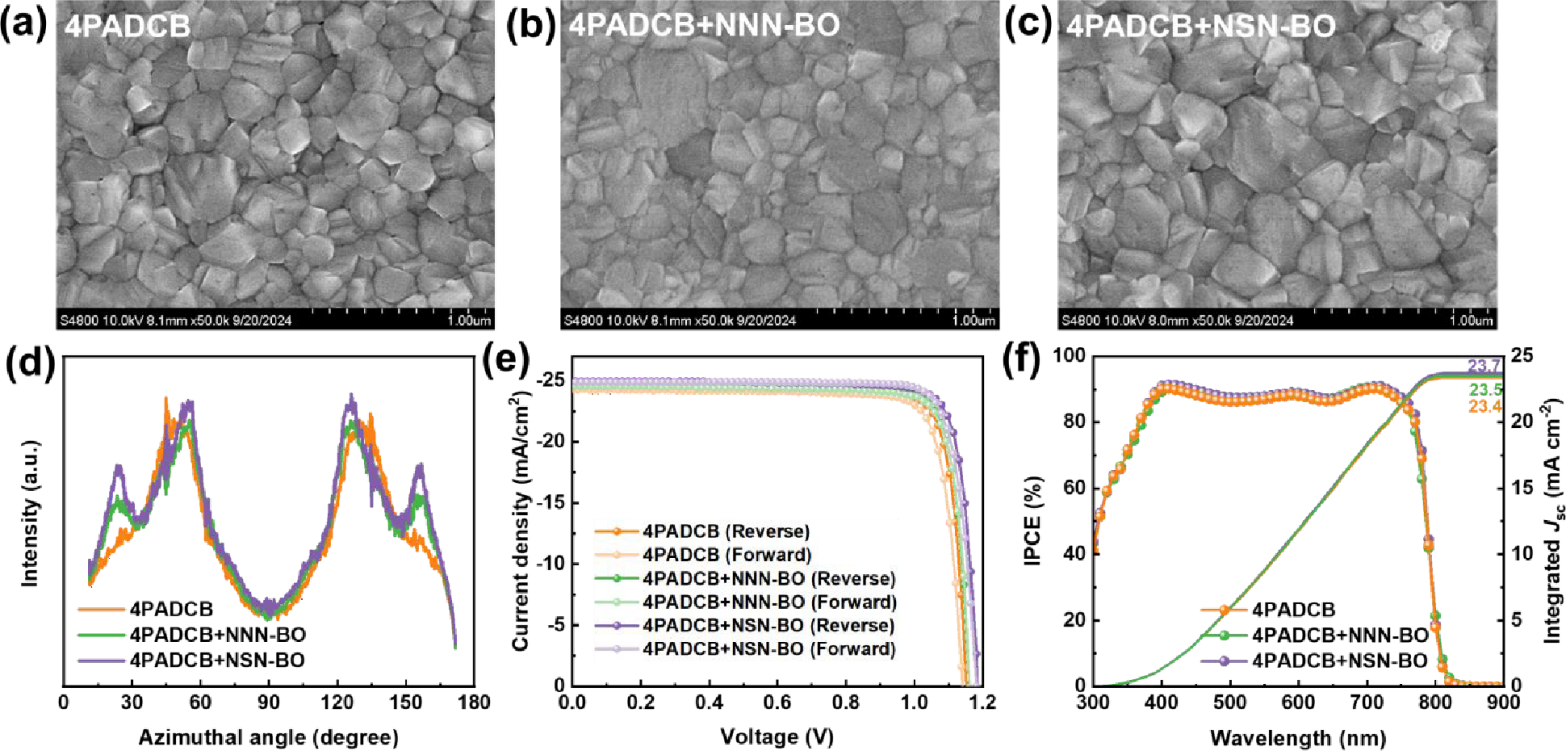
SEM top-view of perovskite films deposited on different substrates and contact angles of perovskite precursor droplets at different interfaces, namely (a) 4PADCB, (b) 4PADCB + NNN-BO, and (c) 4PADCB + NSN-BO. (d) Radially integrated intensity patterns of the ITO/4PADCB or 4PADCB+dispersants/Perovskite films along the (100) plane from the GIWAXS plots. (e) *J–V* characteristics and (f) IPCE spectra of the PSCs.

To further evaluate how the dispersant might affect the perovskite’s composition, we employed XPS to analyze the elemental makeup of the films. From the XPS spectra shown in Figure S14 for the film prepared with and without adding dispersant, no distinct chemical shifts are observed for Cs, C, N, Pb, and I within the perovskite (ABX_3_) framework. We then performed GIWAXS to determine whether the dispersant influences the crystallographic orientation. Figures 5d and S15 shows that perovskite films on NNN-BO and NSN-BO modified SAMs exhibit additional crystal orientations. Furthermore, integration of the (100) peak shows that, in addition to the enhanced crystalline orientation, the main diffraction peak shifts from 49.4° (4PADCB) to 54.6° (4PADCB + NNN-BO) and 54.8° (4PADCB + NSN-BO) with an increase in intensity. These results indicate that the IAM can effectively improve the crystallinity of perovskite films and further increase *V*_OC_ and FF in the PSC devices.^55–58^

Subsequently, we fabricated PSCs using the device architecture MgF_2_/Glass/ITO/NiO_*x*_/4PADCB (with or without dispersants)/Perovskite/Passivation Layer/C_60_/BCP/Ag. As shown by the *J–V* curves in Figure 5e, the champion device employing 4PADCB without dispersants achieves a *V*_OC_ of 1.155 V, a *J*_SC_ of 24.59 mA cm^–2^, an FF of 83.96%, a PCE of 23.84%, and H-index of 3.35. For 4PADCB + NNN-BO, the best-performing device attains *V*_OC_ = 1.162 V, *J*_SC_ = 24.68 mA cm^–2^, FF = 84.26%, PCE = 24.17%, and H-index = 0.79. Impressively, for 4PADCB + NSN-BO the device performance achieves *V*_OC_ = 1.182 V, *J*_SC_ = 24.95 mA cm^–2^, FF = 84.82%, PCE = 25.01%, and H-index = 0.79. A summary of these champion PSCs is provided in Table 2. Moreover, the IPCE spectra (Figure 5f) reveal that both NNN-BO and NSN-BO modifications enhance photon response across the entire wavelength range, confirming that IAM boosts perovskite grain quality and further elevates overall PSCs performance. Likewise, in PSCs, the short-side-chain dispersants NSN-C4 and NSN-BI increase the PCE to 24.95% and 24.94%, respectively, values nearly match the best performance achieved by NSN-BO (see Figure S16). These observations parallel those in OSCs: although the conjugated backbone governs the primary functionality of IAM molecules, the side-chain architecture serves as one of the key determinants of IAM relation to SAM dispersion. Hence, both backbone and side-chain designs warrant meticulous consideration for optimal device performance.

**Table 2:** Photovoltaic Parameters of Champion PSCs^a^

	*V*_OC_ [V]	*J*_SC_ [mA/cm^2^]	FF [%]	PCE [%]	H-index	HC [fs]	PB [ps]	τ_1_ [ns]	τ_2_ [μs]
4PADCB (Reverse)	1.155	24.59	83.96	23.84	3.35	361	492.3	33.0 (62.9%)	5.93 (37.1%)
4PADCB (Forward)	1.138	24.30	83.32	23.04					
4PADCB + NNN-BO (Reverse)	1.162	24.68	84.26	24.17	0.79	337	573.1	31.8 (62.3%)	6.55 (37.7%)
4PADCB + NNN-BO (Forward)	1.162	24.52	84.16	23.98					
4PADCB + NSN-BO (Reverse)	1.182	24.95	84.82	25.01	0.80	331	640.3	29.8 (60.6%)	8.24 (39.4%)
4PADCB + NSN-BO (Forward)	1.182	24.89	84.33	24.81					

a Then TA and TRPL Decay of ITO/4PADCB with or without dispersants/perovskite.

To probe in more detail how the dispersants affect trap density within PSCs, we used hole-only devices (ITO/4PADCB+ dispersants/perovskite/PM6/MoO_3_/Ag) to measure trap-filled limit voltage (*V*_TFL_) based on the space-charge-limited current (SCLC) model. As a result, we derived the trap density (*N*_t_).^59–61^ As depicted in Figure S17, pristine 4PADCB yields *V*_TFL_ = 0.867 V and *N*_t_ = 1.01 × 10^16^ cm^–3^, which decreases to 0.652 V and 7.58 × 10^15^ cm^–3^ upon adding NNN-BO, and further decreases to 0.615 V and 7.15 × 10^15^ cm^–3^ with NSN-BO. These findings demonstrate that the dispersant-based optimization of HSL effectively reduces trap states and defects in the perovskite film. Because high-performance solar cells typically show excellent electroluminescence, we tested the PSCs in light-emitting diode (LED) mode and used a conversion formula (see Supporting Information) to obtain the nonradiative voltage loss (Δ*V*_OC_^nonrad^). Additionally, we measured the full width at half-maximum (fwhm) of the electroluminescence (EL) spectra, where a narrower fwhm indicates more concentrated band-to-band energy distribution, thereby reducing vibronic broadening effect. As shown in Figure S18, at *J*_SC_ = 24.59 mA cm^–2^, devices with pristine 4PADCB exhibit an external quantum efficiency (EQE) of 3.79%, Δ*V*_OC_^nonrad^ = 84.1 meV, and fwhm = 48.9 nm. Incorporating NNN-BO raises the EQE to 4.42%, with Δ*V*_OC_^nonrad^ = 80.1 meV and fwhm = 47.5 nm at *J*_SC_ = 24.68 mA cm^–2^. Lastly, devices incorporating NSN-BO exhibit the highest EQE (5.52%), the lowest Δ*V*_OC_^nonrad^ (74.4 meV), and the narrowest fwhm (47.0 nm) at *J*_SC_ = 24.95 mA cm^–2^. Lastly, electrochemical impedance spectroscopy (EIS) reveals that incorporating dispersants significantly lowers the device’s interfacial resistance: from 35.5 Ω for the unmodified device to 29.4 Ω (NNN-BO) and 15.4 Ω (NSN-BO). Meanwhile, the enhanced perovskite grain structure, facilitated by the increased contact angle, raises the recombination resistance from 1905.0 Ω (pristine) to 8402.8 Ω (NNN-BO) and 10942.1 Ω (NSN-BO) (see Figure S19). Taken together, these results clearly indicate that IAM lowers trap densities and suppresses nonradiative recombination, thereby increasing both *V*_OC_ and FF. We also performed TPV and TPC analyses on the perovskite devices (Figure S20). In agreement with the OSC results, IAM-treated PSCs exhibit longer TPV lifetimes and shorter TPC lifetimes. These consistent trends further support that IAM additives serve as a universal interfacial modifier, enhancing both organic and perovskite solar cells.

We further employed fs-TAS to investigate perovskite samples comprising MgF_2_/glass/ITO/NiO_*x*_/4PADCB (with or without dispersant)/perovskite, excited from the glass side in the 550–600 nm range (pump) and probed within 600–1000 nm. As illustrated in Figure 6a–c, all samples exhibit similar spectral features: the 700–770 nm region corresponds to the photobleaching (PB) of the perovskite, while a positive photoinduced absorption (PIA) signal at 780–830 nm is associated with high-energy hot carriers (HC). To gain deeper insights, we analyzed the transient absorption data from 0.1 to 1000 ps (Figure 6d–f) and calculated the fwhm of the PB signal at 10 ps for 4PADCB, 4PADCB + NNN-BO, and 4PADCB + NSN-BO as 145, 143, and 127 meV, respectively. A narrower PB fwhm suggests fewer background carriers and a more favorable band-to-band alignment,^6,62,63^ which is consistent with the EL findings (vide supra). Figure 6g depicts the hot carrier cooling dynamics, and the corresponding fits indicate that the dispersants do not substantially influence the cooling rate. In contrast, Figure 6h shows that the PB decay time is prolonged by dispersant incorporation, which is from 492.3 ps for pristine 4PADCB to 573.1 ps with NNN-BO, and further extending to 640 ps with NSN-BO. This lengthened decay reflects increased exciton dissociation and free carrier generation, implying enhanced crystallinity in the perovskite films. Finally, time-resolved photoluminescence (TRPL) spectroscopy (Figure 6i) reveals two decay constants: τ_1_, attributed to charge transfer, where a shorter value indicates faster hole transfer to the HSL; and τ_2_, linked to bimolecular recombination, where a longer value suggests a lower recombination rate for free carriers. Specifically, τ_1_ and τ_2_ for pure 4PADCB are 33.0 ns and 5.93 μs, respectively. With NNN-BO, these values become 31.8 ns and 6.55 μs, and for NSN-BO, τ_1_ is further reduced to 29.8 ns while τ_2_ markedly increases to 8.24 μs. From the steady-state photoluminescence (PL) spectra shown in Figure S21, it is evident that perovskite films deposited on 4PADCB + NSN-BO and 4PADCB + NNN-BO exhibit higher emission intensities than those 4PADCB without dispersants. In summary, both fs-TAS and TRPL results consistently demonstrate that dispersant incorporation not only improves the hole transfer capability of HSL but also enhances the crystallinity of the perovskite film, elevating free carrier concentrations and reducing bimolecular recombination. All fs-TAS and TRPL data are summarized in Table 2.

**Figure 6:**
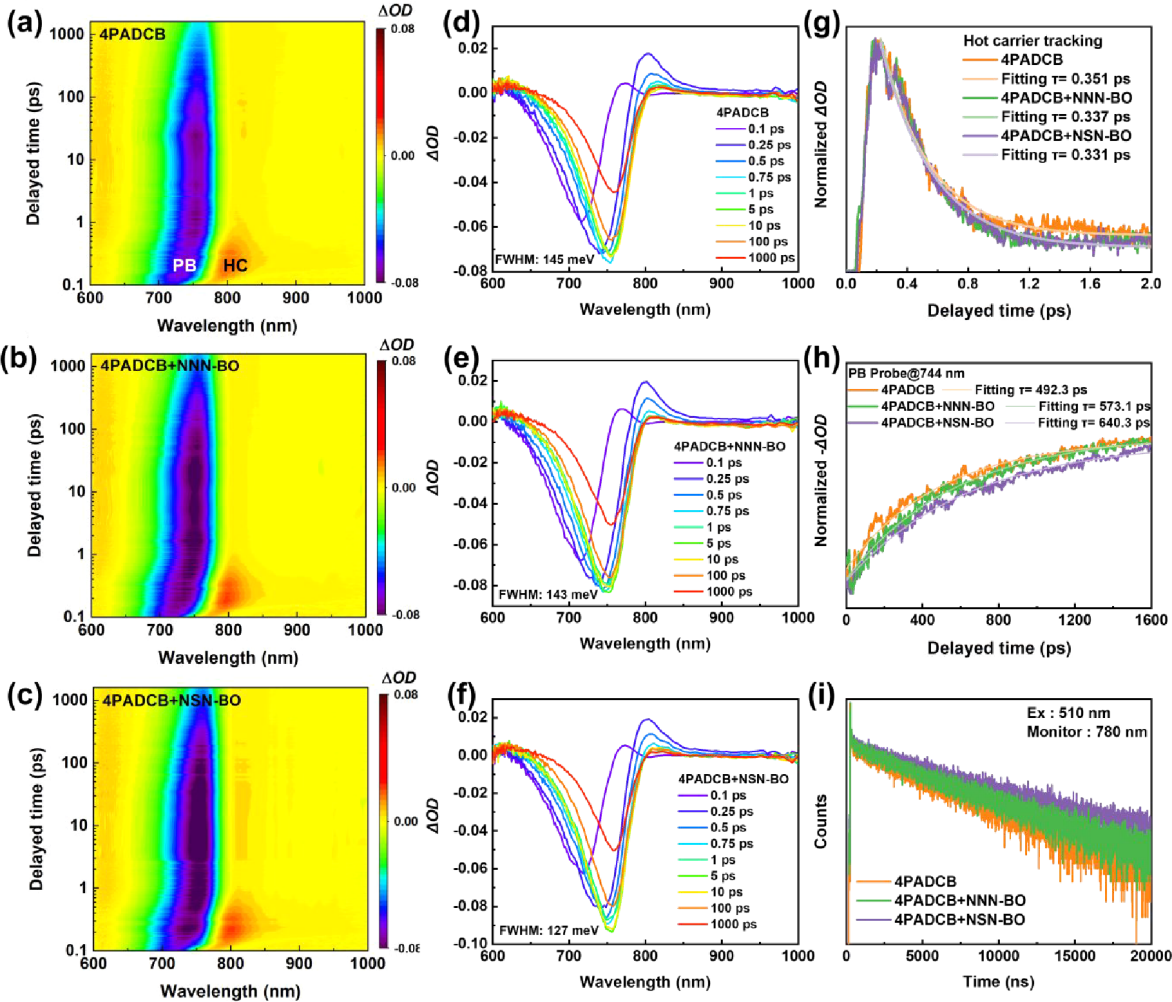
(a–c) 2D fs-TAS plots of the ITO/4PADCB with or without dispersants/perovskite. (d–f) fs-TA spectra of the ITO/4PADCB with or without dispersants/perovskite under glass side excitation. The lifetime of the ITO/4PADCB with or without dispersants/perovskite under (g) hot carrier tracking and (h) photobleaching. (i) Time-resolved photoluminescence spectra of ITO/4PADCB with or without dispersants/perovskite samples excitation with 510 nm and monitored at 780 nm.

Importantly, we evaluated the ambient stability (25 °C, relative humidity of 25 ± 5%) and thermal stability (65 °C under nitrogen) of OSCs and PSCs deposited on 4PADCB, 4PADCB + NNN-BO, and 4PADCB + NSN-BO. As shown in Figure S22, the PSCs incorporating dispersants exhibit markedly improved resistance to both atmospheric exposure and elevated temperature; the same trend is observed for the OSCs. Furthermore, for PSCs and OSCs fabricated and optimization process under various conditions, 16 devices were produced and subjected to reproducibility tests, with the statistical data presented in Figures S23–S32. Overall, introducing IAM effectively enhances the performance and stability of next-generation photovoltaic devices, demonstrating strong potential for practical applications.

## Conclusions

In summary, we have successfully designed and synthesized two types of solid dispersants, NNN-BO and NSN-BO, whose chemical backbones closely resemble that of the host SAM, 4PADCB, thereby enabling the formation of IAMs (Scheme 1). We propose that a well-designed IAM should meet two key criteria: (1) Effective push–pull interactions through strong intermolecular interactions between the dispersants (NNN-BO and NSN-BO) and the host SAM (4PADCB). (2) Enhanced hole-transfer efficiency via dispersants possessing higher dipole moments than the host SAM. Specifically, NSN-BO, with its notably high dipole moment of 6.48 D, not only impedes micelle formation but also considerably improves hole transport.

A proof-of-concept was performed by introducing NNN-BO and NSN-BO in PM6:Y6 OSC, improving the PCE from 16.46 to 16.72% (NNN-BO) and 18.04% (NSN-BO). Furthermore, applying the IAM strategy in PSCs improved the PCE from 23.84 to 24.17% (NNN-BO) and 25.01% (NSN-BO), highlighting the key role of the dispersant dipole moment in device efficiency. Also, systematic study on the influence of side-chain length was carried out by comparative study of NSN-C4, NSN-IB and NSN-BO in PM6:L8-BO-based OSCs, with PCEs reaching 18.94, 19.01, and 19.23%, and in PSCs with PCEs of 24.94, 24.95 and 25.01%, respectively. Taken together, our results imply that side-chain length represents a secondary design parameter influencing IAM efficacy: longer side chains confer greater steric hindrance, which enhances dispersion within the host-SAM and ultimately supports superior device operation. Importantly, stability under ambient and thermal conditions was significantly improved in all IAM-integrated devices. We then elucidate the underlying IAM enhancement mechanism through fs-TAS dynamics in two systems (OSC and PSC), confirming the critical role of these dispersants in higher dipole moments, which can greatly shorten the hole transport time, thereby promoting efficient charge extraction and optimizing film morphology. One of the key merits of IAM molecules is their potential to serve as a universal secondary component for hole-selective layer in both organic and perovskite solar cells, thus affording a more systematic and unified strategy for material design. It is worth noting that the IAM concept presented here is just a prototype. From a chemical perspective, the design and synthesis of suitable dispersants for specific SAM molecules should be a facile and practical strategy worth promoting.

## Supplementary Material



## Notes

Experimental section includes reagents and materials, detailed measurements, computational details, crystal preparation, device fabrication, and characterization; ^1^H NMR spectra,^13^C NMR spectra, and mass spectra for all compounds; and additional references (PDF)
