# Supplementary material for: Interspersed Assembled Monolayers Enhance Hole Transport in High-Efficiency Organic and Perovskite Solar Cells
Source: J Am Chem Soc. 2025 Jun 26;147(27):23683–95. doi: 10.1021/jacs.5c05341 (PMC12257515; doi:10.1021/jacs.5c05341)
Supplement: Supplementary file 1 [file ja5c05341_si_001.pdf]

Supporting Information

Interspersed Assembled Monolayers Enhance Hole Transport in High-Efficiency Organic and Perovskite Solar Cells

Chieh-Ming Hung[1,+], Jing-Han Shi[1,+], Hsiao-Chun Tsai[1], Chi-Ping Lin[1], Bo-Han Chen[2], Shang-Da Yang[2], Pi-Tai Chou[1,*]

[1] C.-M. Hung, J.-H. Shi, H.-C. Tsai, C.-P. Lin, P.-T. Chou

Department of Chemistry, Center for Emerging Materials and Advanced Devices, National Taiwan University, Taipei, 106319 Taiwan

[2] B.-H. Chen, S.-D. Yang

Institute of Photonics Technologies, National Tsing Hua University, Hsinchu 300044, Taiwan

[+] These authors contributed equally to this work.

*e-mail: chop@ntu.edu.tw

Table of Contents

1. Experiment Section………………………………………………………. S3
   Chemicals………………………………………………………………… S3
   Device Fabrication of Organic Solar Cells………………………………. S3
   Perovskite Thin Film Fabrication………………………………………… S4
   Device Fabrication of Inverted Perovskite Solar Cells…………………… S4
   Characterizations…………………………………………………………. S5
   Setup of Light Source and Transient Absorption Spectroscopy…………. S6
2. Supporting Figures, and Tables…………………………………………... S8
3 Synthesis and Characterization of Compounds…………………………... S29
4 X-ray Crystallography……………………………………………………. S59
5. References………………………………………………………………… S64

Chemicals:

Cesium chloride (CsCl, ultra dry, 99.999%), formamidinium iodide (FAI, 99.99%), and methylammonium bromide (MABr, 99.99%) were purchased from Dyesol. Phenethylammonium chloride (PEACl, 99.9%), lead iodide (PbI_2, 99.99%), and lead bromide ($PbBr_2$, 99.99%) were obtained from TCI. Nickel(II) oxide nanoparticle 5 mg/mL solution (IPA:H_2O = 1:3) and (4-(7*H*-dibenzo[*c,g*]carbazol-7-yl)butyl)phosphonic acid (4PADCB, 99%) were sourced from Lumtec. poly[(2,6-(4,8-bis(5-(2-ethylhexyl-3-fluoro)thiophen-2-yl)-benzo[1,2-*b*:4,5-*b*']dithiophene))-alt-(5,5-(1',3'-di-2-thienyl-5',7'-bis(2-ethylhexyl)benzo[1',2'-*c*:4',5'-*c*']dithiophene-4,8-dione)] (PM6), Poly[(2,6-(4,8-bis(5-(2-ethylhexyl-3-fluoro)thiophen-2-yl)-benzo[1,2-*b*:4,5-*b*']dithiophene))-alt-5,5'-(5,8-bis(4-(2-butyloctyl)thiophen-2-yl)dithieno[3',2':3,4;2'',3'':5,6]benzo[1,2-*c*][1,2,5]thiadiazole)] (D18), 2,2'-((2*Z*,2'*Z*)-((12,13-bis(2-ethylhexyl)-3,9-diundecyl-12,13-dihydro-[1,2,5]thiadiazolo[3,4-e]thieno[2'',3'':4',5']thieno[2',3':4,5]pyrrolo[3,2-g]thieno[2',3':4,5]thieno[3,2-*b*]indole-2,10-diyl)bis(methanylylidene))bis(5,6-difluoro-3-oxo-2,3-dihydro-1*H*-indene-2,1-diylidene))dimalononitrile (Y6, 99%), 2,2'-((2*Z*,2'*Z*)-((12,13-bis(2-ethylhexyl)-3,9-(2-butyloctyl)-12,13-dihydro-[1,2,5]thiadiazolo[3,4-e]thieno[2'',3'':4',5']thieno[2',3':4,5]pyrrolo[3,2-g]thieno[2',3':4,5]thieno[3,2-*b*]indole-2,10-diyl)bis(methanylylidene))bis(5,6-difluoro-3-oxo-2,3-dihydro-1*H*-indene-2,1-diylidene))dimalononitrile (L8-BO, 99%) and N,N'-Bis{3-[3-(Dimethylamino)propylamino]propyl}perylene-3,4,9,10-tetracarboxylic diimide (PDINN) were sourced from Solarmer. C_{60} (99%) and bathocuproine (BCP, 99.9%) were purchased from Sigma Aldrich. All liquid solvents, including *N*,*N*-dimethylformamide (DMF, anhydrous, 99.9%), dimethyl sulfoxide (DMSO, 99.9%), isopropanol (IPA, anhydrous, 99.9%), chlorobenzene (CB, anhydrous, 99.9%), chloroform (CF, anhydrous, 99.5%), ethanol (EtOH, anhydrous, 99.5%), and methanol (MeOH, anhydrous, 99.5%) were also obtained from Sigma Aldrich.

Device Fabrication of Organic Solar Cells:

The inverted perovskite solar cells were fabricated with the structure MgF_2/ITO/ /4PADCB+NNN-BO or NSN-series (10:1)/Active layer /PDINN/Ag. Patterned ITO-coated glass substrates (10 Ω per square) were successively cleaned by ultrasonication in 1% neutral detergent in water, deionized water, acetone, and isopropanol for 20 min each, followed by drying under a stream of dry nitrogen. The self-assembled monolayer (SAM) solution of 4PADCB: dispersants (10:1) with a total concentration of 1.1 mg/mL in EtOH:CF (4:1) was spin-coated (take 100 μL) at 0 rpm for 15 seconds then 3000 rpm for 30 seconds, followed by annealing at 120 °C for 10 min. Sequentially, the active layer solution of PM6:Y6 (1:1.2 in chloroform, total concentration: 17.6 mg/mL and 50

℃ stir for 2 hr) was dynamic spin-coated (take 18 μL) at 3000 rpm for 30 s then annealing at 100 ℃ for 10 min. or D18:Y6 (1:1.4 in chloroform, total concentration:9.6 mg/ml and 80 ℃ stir for 2 hr) was dynamic spin-coated (take 18 μL) at 2500 rpm for 30 s. PM6:L8-BO (1:1.2 in chloroform, total concentration: 16.5 mg/ml and 45 ℃ stir for 2 hr) was dynamic spin-coated (take 18 μL) at 4500 rpm for 30 s. After that, PDINN methanol solution with a concentration of 1.0 mg mL^{-1} was dynamic spin-coated on the active layer at 3000 rpm for 30 s. Finally, 120 nm silver electrode were sequentially evaporated under high vacuum (< 1 × 10^{-6} torr). The effective area of one cell was 0.1 cm^2. The opening area of the mask is 0.04 cm^2. Please note that the performance of OSCs depends on the polymer donor, and different suppliers may provide polymer donors with varying formulations, leading to differences in device performance.

Perovskite Thin Film Fabrication:

To fabricate the perovskite thin film, the precursor solution was prepared by dissolving 228.76 mg of FAI, 677.67 mg of PbI_2, 23.57 mg of CsCl, and 7.84 mg of MABr in 0.85 mL of DMF and 0.15 mL of DMSO. An aliquot of 85 μL of this precursor solution was then spin-coated onto the substrate at 5000 rpm for 30 s. During the spin-coating process, 250 μL of CB was dripped onto the film as an anti-solvent 15 s before the end of the spin-coating procedure. Finally, the substrate was annealed at 110 °C for 20 min to form the perovskite film.

Device Fabrication of Inverted Perovskite Solar Cells:

The inverted perovskite solar cells were fabricated with the structure MgF_2/ITO/NiO_x/4PADCB+NNN-BO or NSN-series (10:1)/perovskite/PEACl/C_{60}/BC-P/Ag. Patterned ITO-coated glass substrates (10 Ω per square) were successively cleaned by ultrasonication in 1% neutral detergent in water, deionized water, acetone, and isopropanol for 20 min each, followed by drying under a stream of dry nitrogen. First, a 300 nm MgF_2 film was vacuum-deposited on the glass side at high vacuum (< 1 × 10^{-6} torr). Then, DI water was dropped onto the ITO side and spin-coated at 5000 rpm for 30 s. The substrates (ITO side) were then subjected to UV−ozone treatment for 20 min. The nickel oxide (NiO_x) solution was filtered through a 0.22 μm PVDF membrane and spin-coated onto ITO at 3000 rpm for 30 s, followed by annealing at 150 °C for 30 min, then transferred into an N_2-filled glove box (< 0.1 ppm O_2 and H_2O). The self-assembled monolayer (SAM) solution of 4PADCB: dispersants (10:1) with a total concentration of 0.55 mg/mL in EtOH:CF (4:1) was spin-coated (take 100 μL) at 0 rpm for 15 s then 3000 rpm for 30 s, followed by annealing at 120 °C for 10 min. Perovskite thin films were constructed using the same method described in the Perovskite Thin Film Fabrication section. After that, the passivation layer, after

perovskite deposition, 1.25 mg/mL PEACl in IPA:DMF (99:1) was dynamic spin-coated (take 30 μL) at 5000 rpm for 30 s and annealed at 110 °C for 5 min. After the film cooled down to room temperature, Finally, a 25 nm C_{60}, 4 nm BCP layer and a 120 nm silver electrode were sequentially evaporated under high vacuum (< 1×10^{-6} torr). The effective area of one cell was 0.1 cm^2. The opening area of the mask is 0.04 cm^2.

Characterization:

The *J–V* curves were measured using a Newport (Sol3A Class AAA Solar Simulator) AM 1.5 G light source operating at 100 mW/cm^2 and independently verified with a 300-W AM1.5 G source also operating at 100 mW/cm^2. The light intensity was determined using a mono-silicon detector with a KG-5 visible color filter, calibrated by the National Renewable Energy Laboratory (NREL) to minimize spectral mismatch. The monochromatic incident photon-to-electron conversion efficiency (IPCE) spectra were measured using a lock-in amplifier with a current preamplifier under short-circuit conditions, supplied by PV Measurement. Devices were illuminated with monochromatic light from a xenon lamp passing through a monochromator, typically at an intensity of 30 μW. A calibrated mono-silicon diode with a known spectral response was used as a reference. The chemical composition and interactions of the perovskite films were confirmed using X-ray photoelectron spectroscopy (XPS) measurements (ULVAC-PHI, Japan). Scanning electron microscopy (SEM) images were captured by Hitachi S4800. Grazing incidence wide-angle X-ray scattering (GIWAXS) patterns were obtained at beamline BL13A1 of the National Synchrotron Radiation Research Center (NSRRC) in Taiwan. The scattering patterns were collected using a Mar165 CCD with a diameter of 40 mm. The scattering vector, $q = 4\pi/\lambda \sin\theta$, and the scattering angles in these patterns were calibrated using silver behenate. Absorption spectra were obtained with a Hitachi UH-5700 spectrophotometer. Steady-state photoluminescence (PL) spectra and time-resolved studies were performed using a time-correlated single photon counting (TCSPC) system (FLS 980, Edinburgh). Electrochemical impedance spectroscopy (EIS) resistance analysis was conducted using a Metrohm Autolab in an ambient environment at 0.9 V under dark conditions and analyzed with NOVA 2.0 software. The trap-filled limit (V_{TFL}) was measured under dark conditions using a Keithley 2400 Source Meter. Electroluminescence spectra were characterized using the LQ-50X system (Enlitech, Taiwan), which includes a PTFE integrating sphere, a Multi-Channel Photon Detector (MCD), and two spectrometers for collecting emission photons and conducting subsequent spectral analyses. The MCD enhances sensitivity, allowing for effective detection in low-light conditions. The system can measure a broad wavelength range from 300 to 1700 nm using Si and InGaAs detectors, calibrated against a NIST-traceable standard lamp. The V_{OC} loss

from nonradiative recombination (ΔV_{OC}^{nrad}) can be calculated from the external quantum efficiency (EQE_{EL}) at the same operation current density as PSC's J_{SC} with equation ΔV_{OC}^{nrad} =(-k_BT/e) × ln (1/EQE_{EL}) where k_BT/e is equal to 0.0257 eV. Cyclic voltammetry (CV) analyses were conducted to verify the intrinsic electronic properties of zwitterion derivatives. An Ag/Ag^+ (0.01 M $AgNO_3$) electrode was used as the reference electrode. The oxidation potentials were measured using a platinum electrode in tetrahydrofuran as the working electrode, with 0.1 M $[NBu_4]^+PF_6^-$ as the electrolyte and platinum wire as the counter electrode. The potentials were further referenced to the ferrocenium/ferrocene (Fc^+/Fc) couple (E_{OX}^{Fc} = 0.16 V). The HOMO energy levels (E_{HOMO}) were calculated using the equation $E_{HOMO} = -(E_{OX} - E_{FC} + 4.8)$ (eV). Theoretical calculations all the calculations of the model compounds studied in this work were performed using the Gaussian 09 software package. Ground state geometry optimizations of 4PADCB, NNN-BO, and NSN are calculated by DFT at the B3LYP/6-31G (d,p) basis set. The visualization of the molecular orbitals and ESP distribution was performed using GaussView 6.0.16.

Setup of Light Source and Transient Absorption Spectroscopy:

The detailed schematic setups of our light source and transient absorption spectroscopy are illustrated in our previous study.[1] The details are summarized as follows. The measurements were conducted using a commercial Yb:KGW laser system (Pharos, Light Conversion) with a central wavelength of 1030 nm, an average power of 2.5 W, a repetition rate of 3.125 kHz, a pulse energy of 800 μJ, and a pulse duration of 190 fs. Two identical pulses were generated using a low-GDD 50/50 beam splitter and then passed through our designed nonlinear compressor, employing a technique known as multiple plate compression (MPC).[2,3] A high-pass filter with a cut-off wavelength of 980 nm was applied for this experiment. Pulse compression was achieved with 8 chirped mirror bounces (Ultrafast Innovation) to remove the material dispersion introduced by the optics before the sample. The pump pulse was modulated by a laser-triggered mechanical chopper at half the laser repetition rate (1.5625 kHz). A broadband half-wave plate and wire-grid polarizer were employed to precisely control the excitation power and prevent any nonlinear effects. The delay time of the probe pulse relative to the pump pulse was adjusted using a linear translation stage (DL325, Newport) with a delay range of up to approximately 2.2 ns. The pump and probe beams were focused on the sample in a noncollinear manner with a cross-angle of 5°. Different focusing conditions were chosen for the pump and probe pulses to ensure that the focused pump spot size (~67.7 μm) was slightly larger than the focused probe spot size (~27.3 μm), ensuring uniform excitation of the probed region. After passing through the sample, the transmitted probe pulse was spatially separated and guided into our

designed spectrometer. The spectrometer included a high-speed linear array camera (Glaz Linescan-I-Gen2, Synertronic with S12198-512Q CMOS, Hamamatsu) to capture each probe pulse. Since the pump pulses were modulated at half the repetition rate, the spectral difference between every two probe shots (one exposed to the pump and the other not) provided the ΔT/T signal.

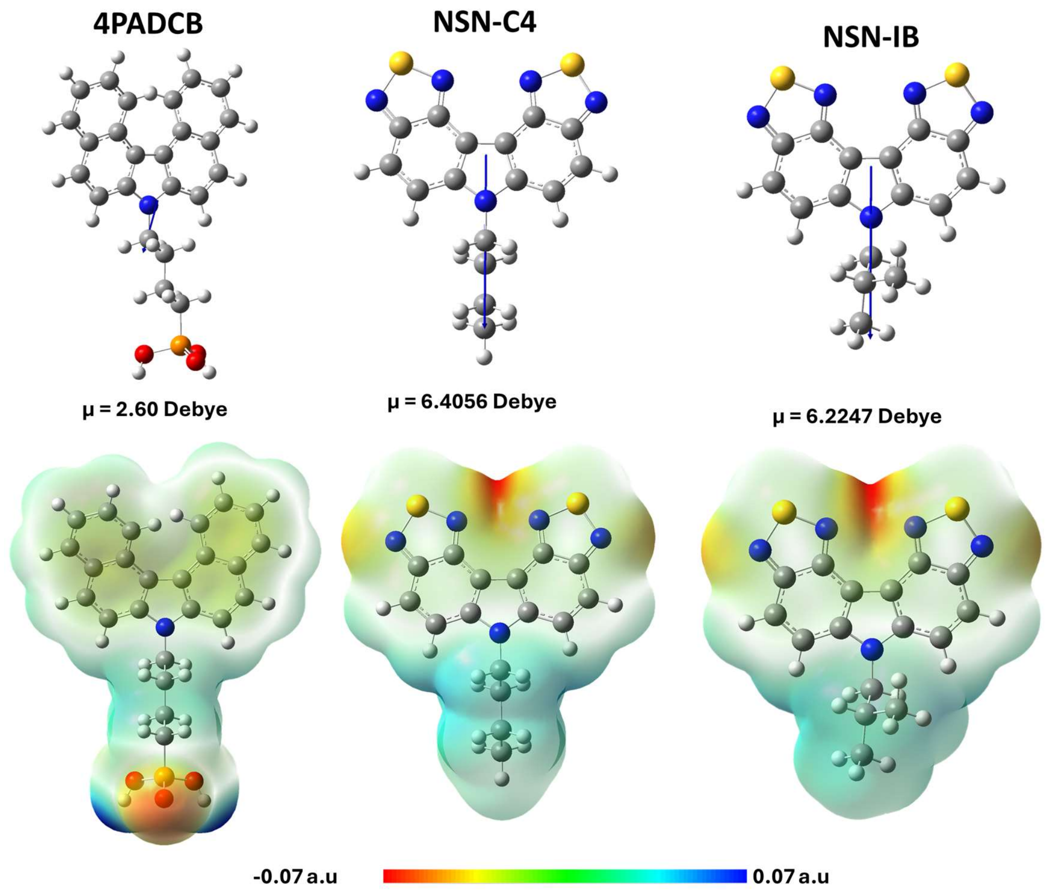

Figure S1. Schematic illustration of the dipole moment and ESP vectors in the 4PADCB, NSN-C4 and NSN-IB.

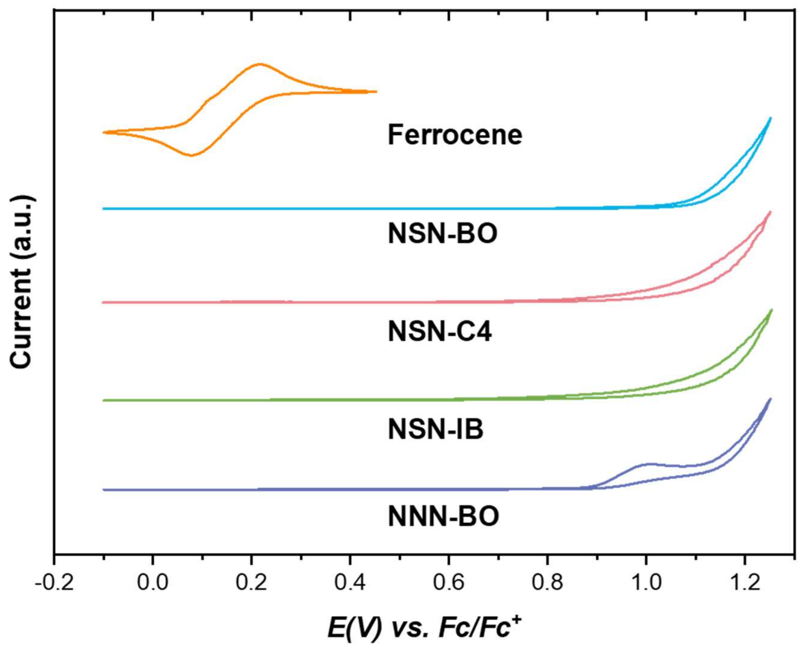

Figure S2. CVs of NNN-BO and NSN-series compounds in THF/0.1 M TBAPF$_6$ under nitrogen.

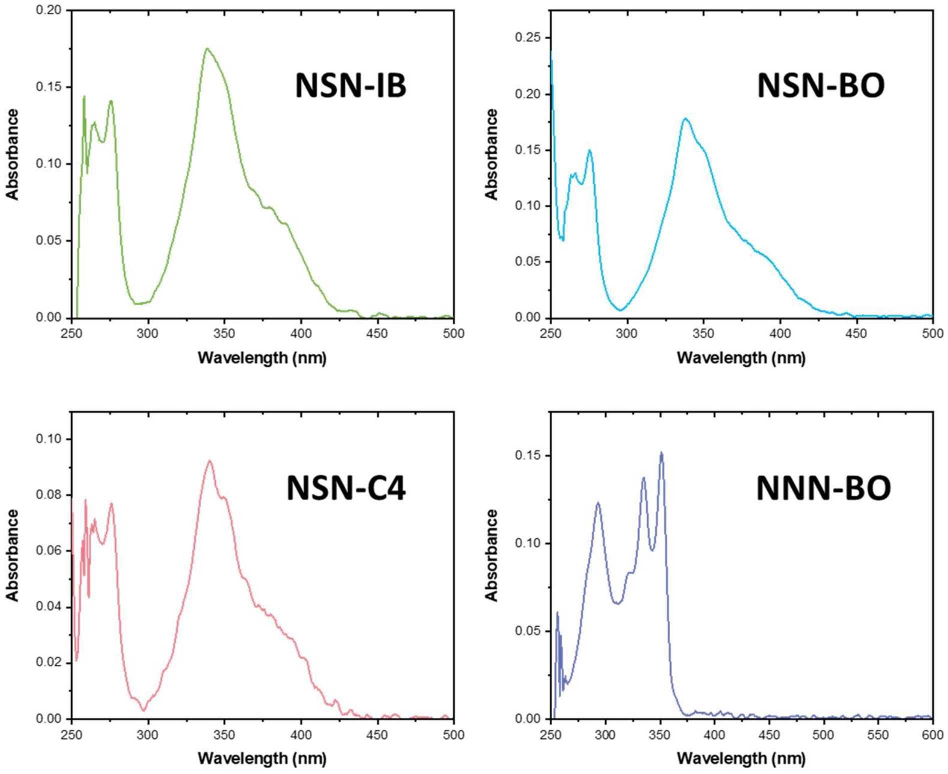

Figure S3. UV-visible absorption spectra of the NNN-BO and NSN-series in THF.

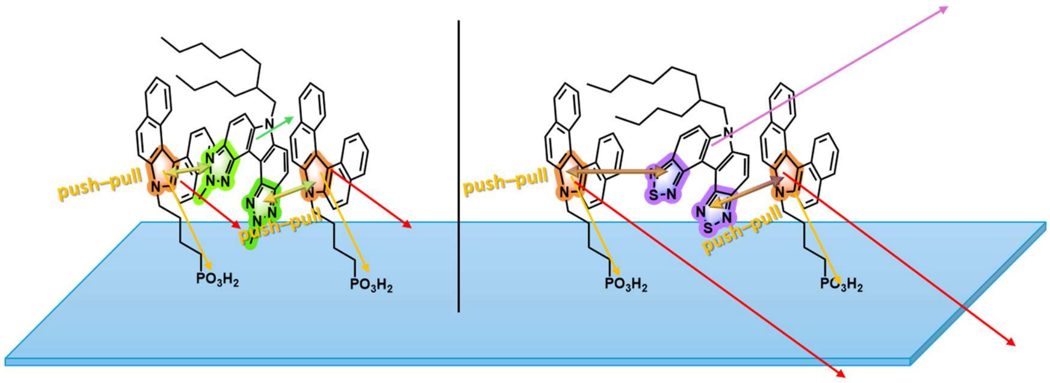

Figure S4. The schematic illustration of the intermolecular push-pull interactions and vector accumulation of dipole moments between the dispersant and 4PADCB. The orange, green, purple and red arrows are respectively: 4PADCB, NNN-BO, NSN-BO, and the accumulated dipole moment vector.

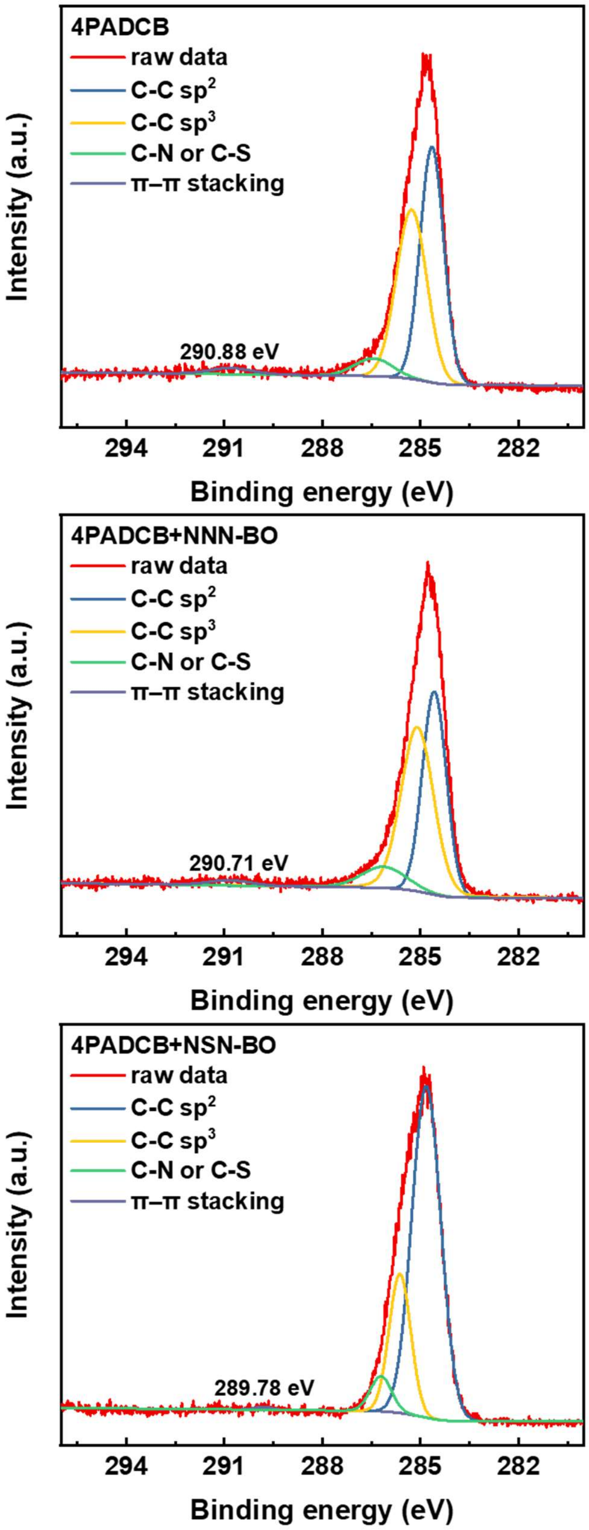

Figure S5. XPS spectra of the core-level C 1s element of 4PADCB, 4PADCB+NNN-BO, and 4PADCB+NSN-BO.

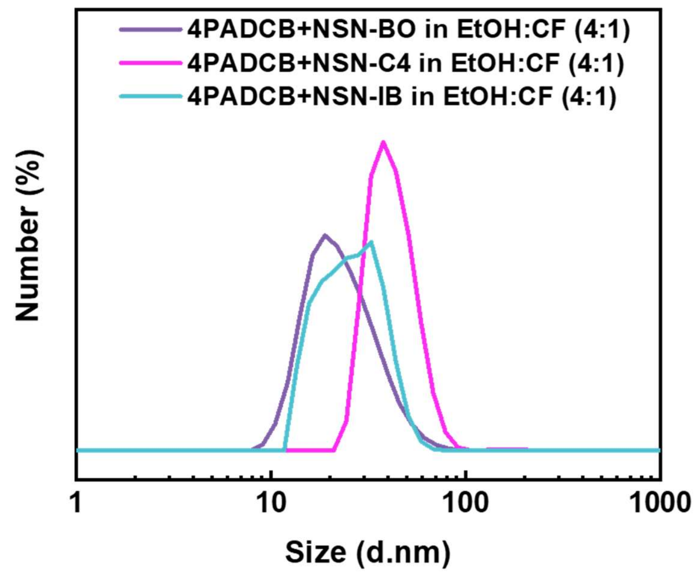

Figure S6. DLS data for 4PADCB in the presence of dispersants NSN-C4 and NSN-IB.

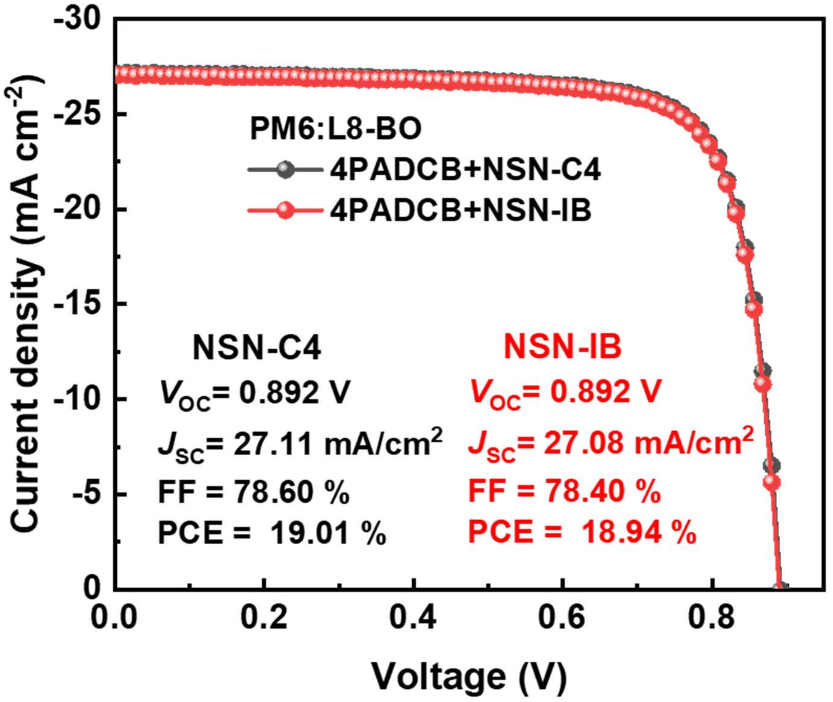

Figure S7. *J-V* curves and photovoltaic parameters of 4PADCB+NSN-C4 or NSN-IB with PM6:L8-BO as active layer OSCs.

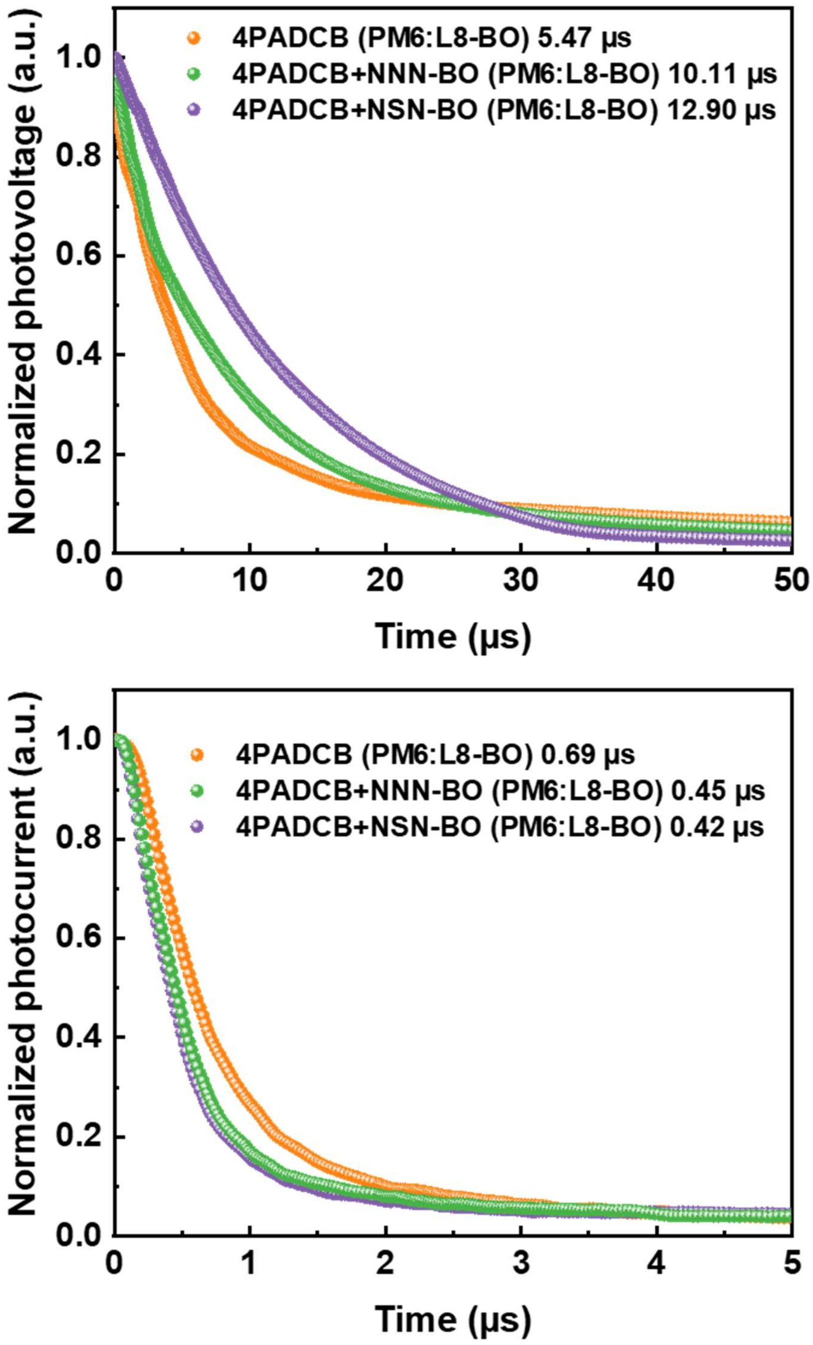

Figure S8. OSCs (PM6:L8-BO) with or without IAM in terms of transient photovoltage measurements and transient photocurrent measurements.

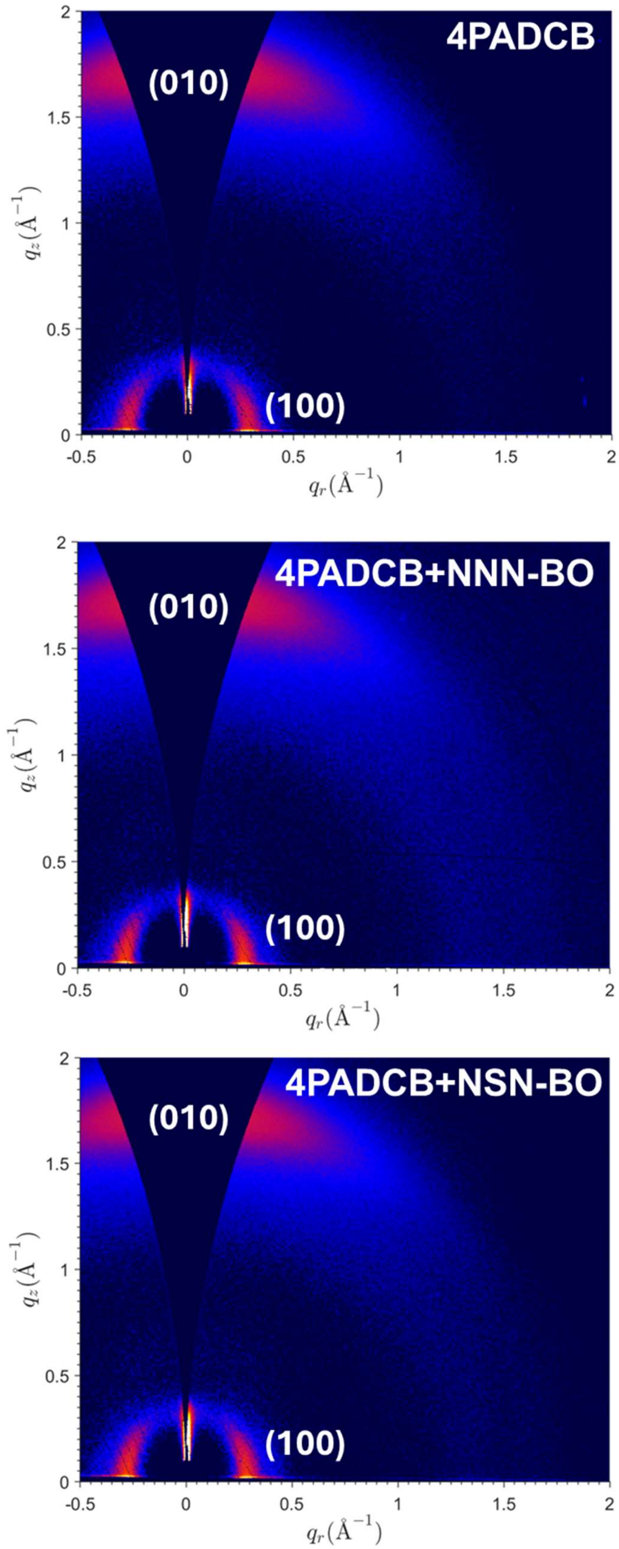

Figure S9. 2D-GIWAXS patterns for ITO/4PADCB with or without dispersants/PM6:Y6.

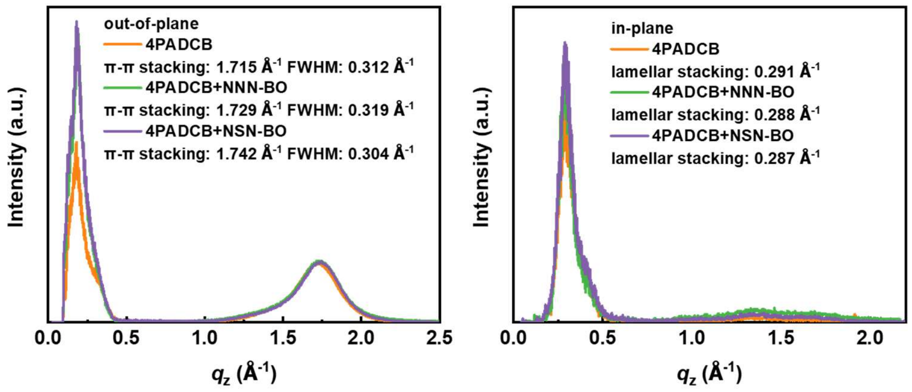

Figure S10. The out-of-plane and in-plane profiles of GIWAXS patterns for ITO/4PADCB with or without dispersants/PM6:Y6.

Table S1. Summary of Packing Parameters Derived from GIWAXS Measurements.

	(010) d-spacing	(010) CCL	(100) d-spacing
4PADCB	3.66	18.72	21.58
4PADCB+NNN-BO	3.63	18.78	21.80
4PADCB+NSN-BO	3.60	19.21	21.88

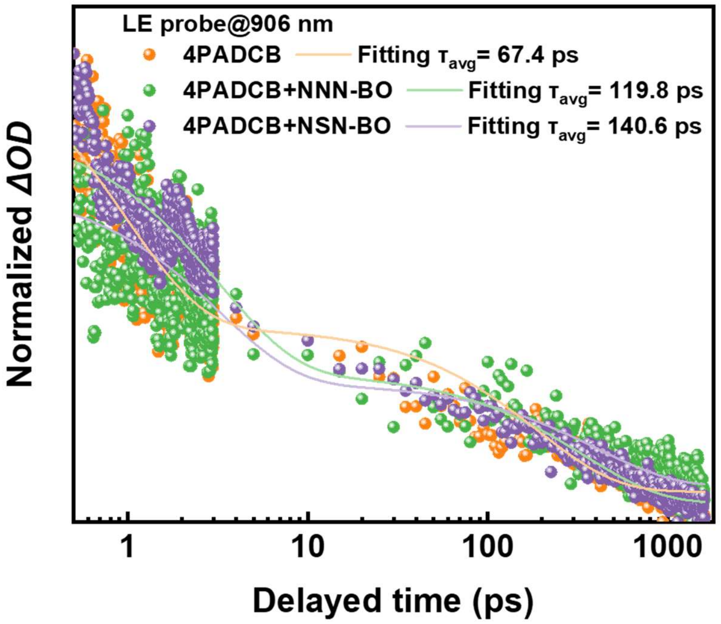

Figure S11. The decay lifetime of ITO/4PADCB with or without dispersants/PM6:Y6 films under LE signal at 906 nm.

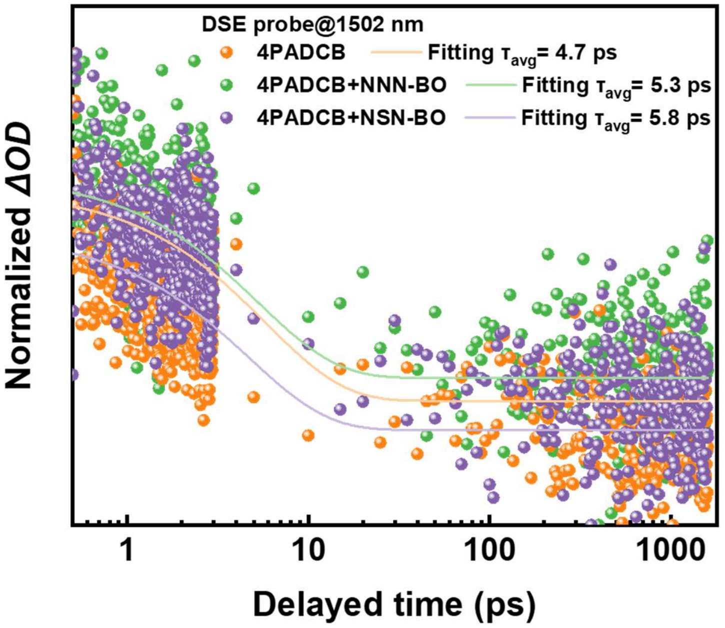

Figure S12. The decay lifetime of ITO/4PADCB with or without dispersants/PM6:Y6 films under DSE signal at 1502 nm.

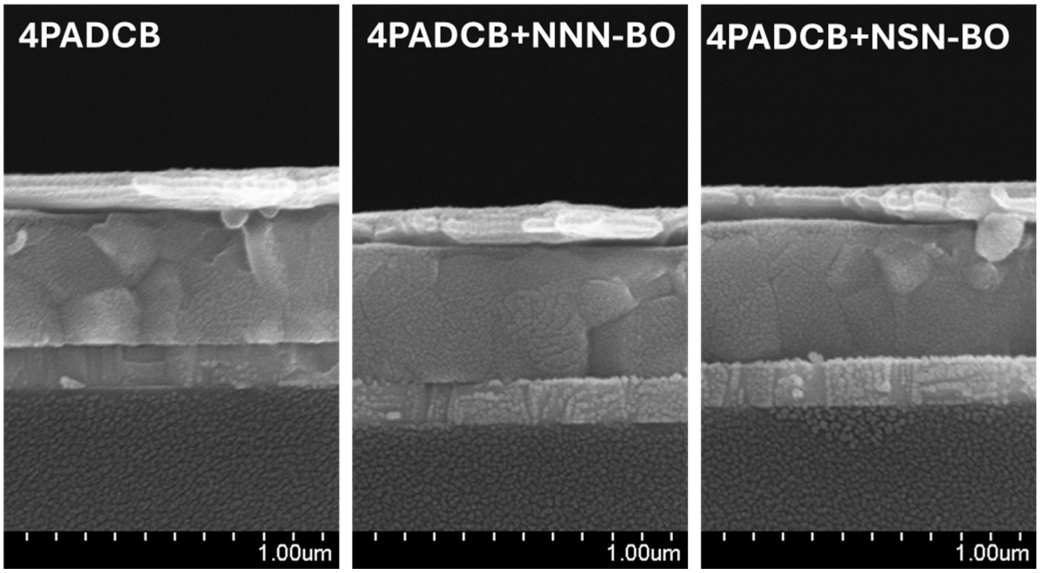

Figure S13. SEM cross-sectional images of perovskite solar cells based on the SAM with or without IAM.

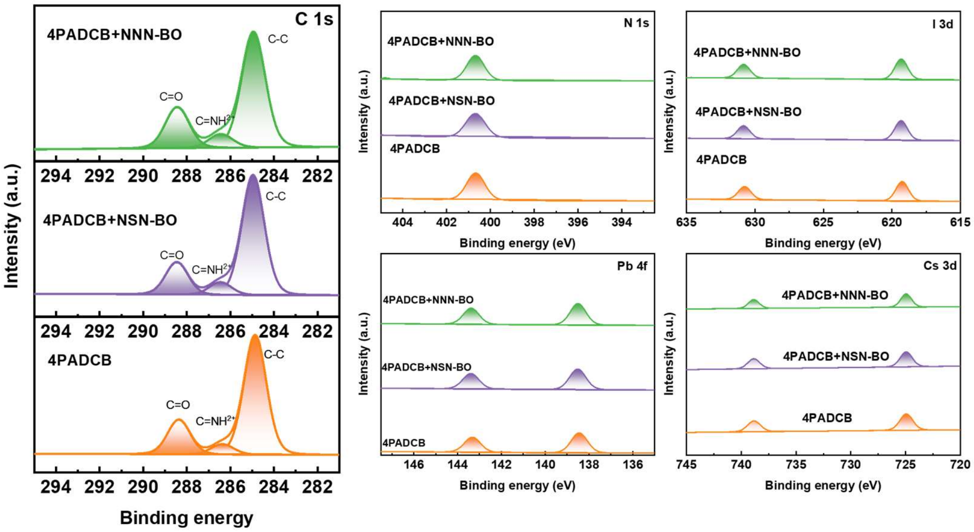

Figure S14. XPS spectra were measured on the ITO/NiO_x/4PADCB with or without dispersants/Perovskite.

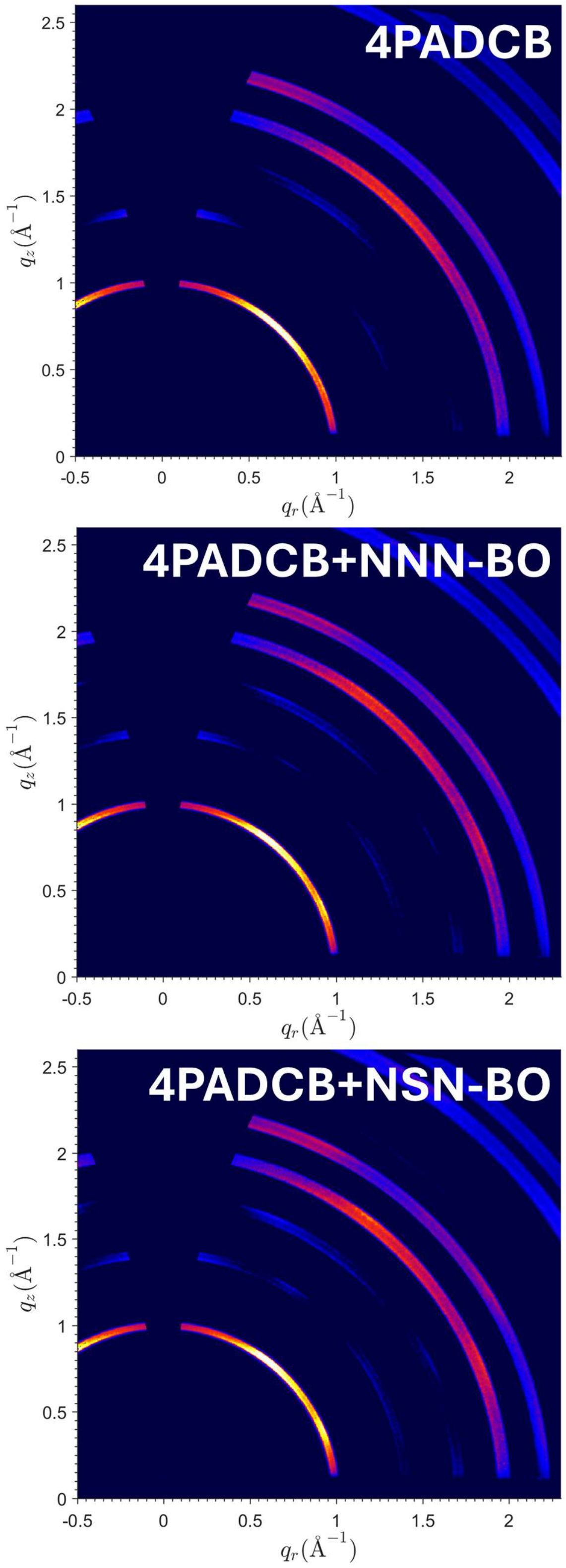

Figure S15. 2D-GIWAXS patterns for ITO/ NiO_x/4PADCB with or without dispersants/ Perovskite.

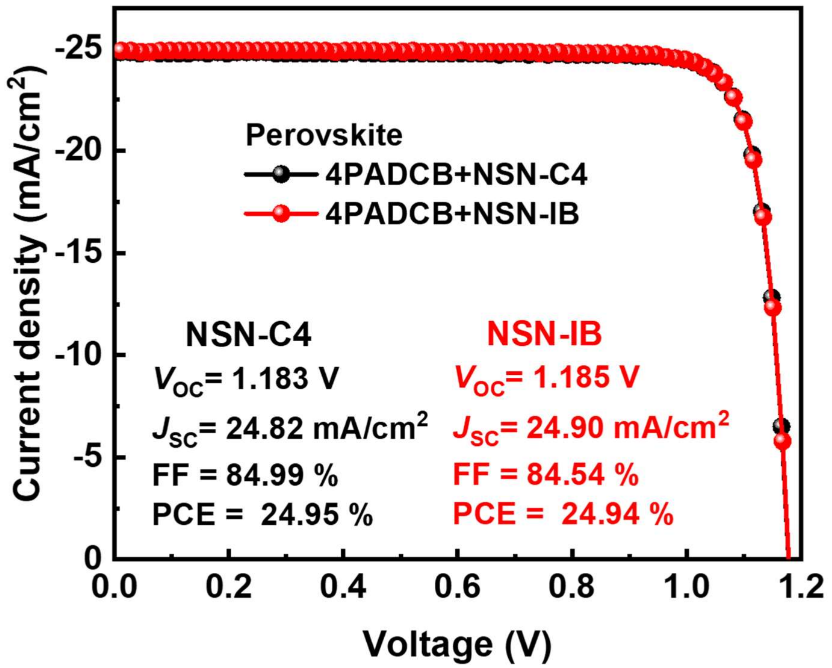

Figure S16. *J-V* curves and photovoltaic parameters of 4PADCB+NSN-C4 or NSN-IB with PSCs.

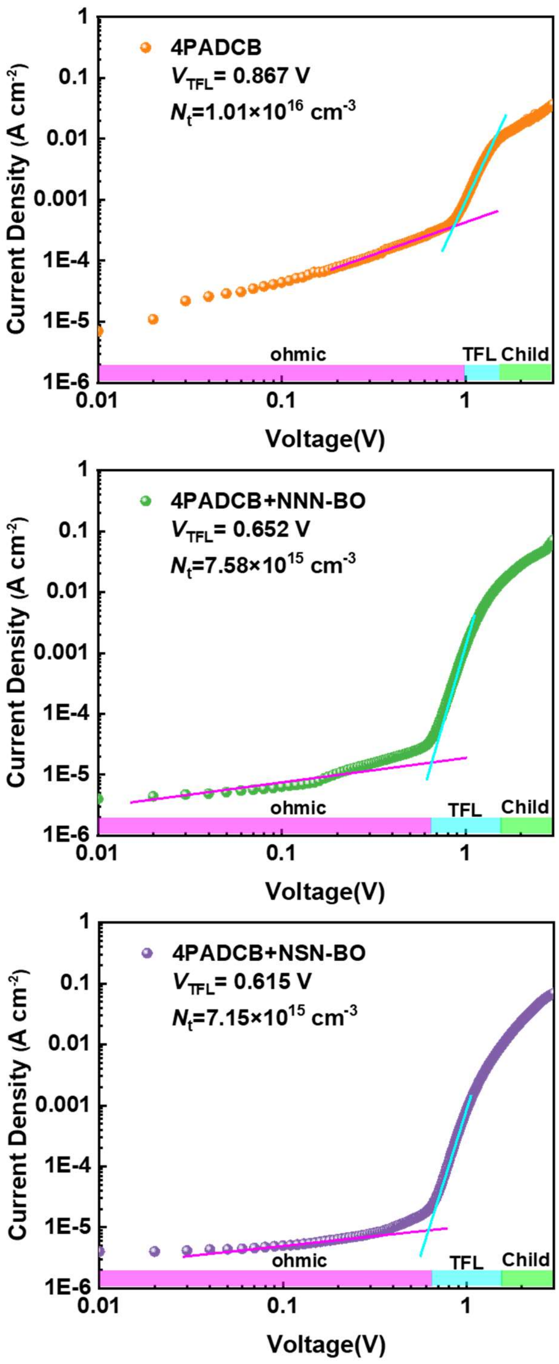

Figure S17. Hole-only devices for the ITO/ NiO_x/4PADCB with or without dispersants/ Perovskite/PM6/MoO_3/Ag.

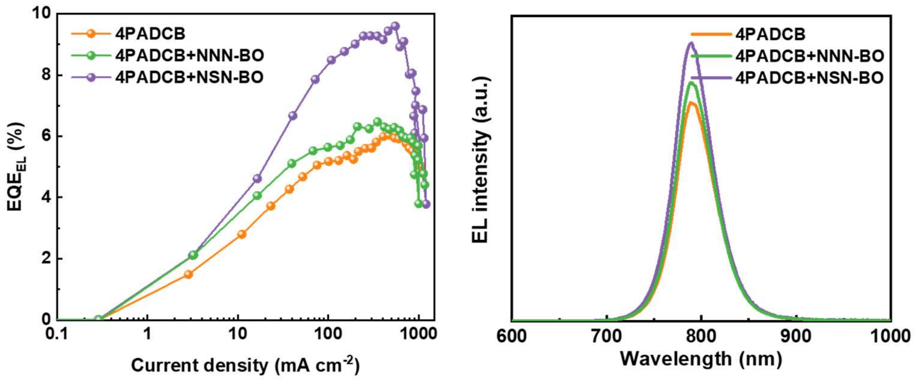

Figure S18. The External Quantum Efficiency Electroluminescence (EQE_{EL}) and Electroluminescence (EL) spectra measurements of 4PDACB and 4PDACB+NNN-BO or NSN-BO-treated PSCs.

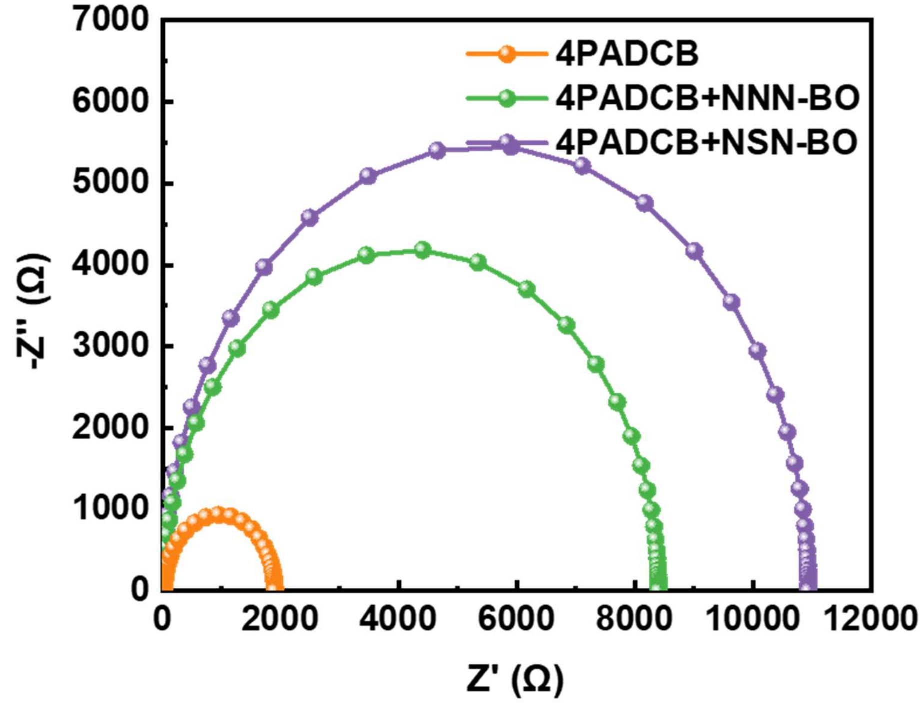

Figure S19. EIS plot of PSCs based on 4PDACB and 4PDACB+NNN-BO or NSN-BO as HSL.

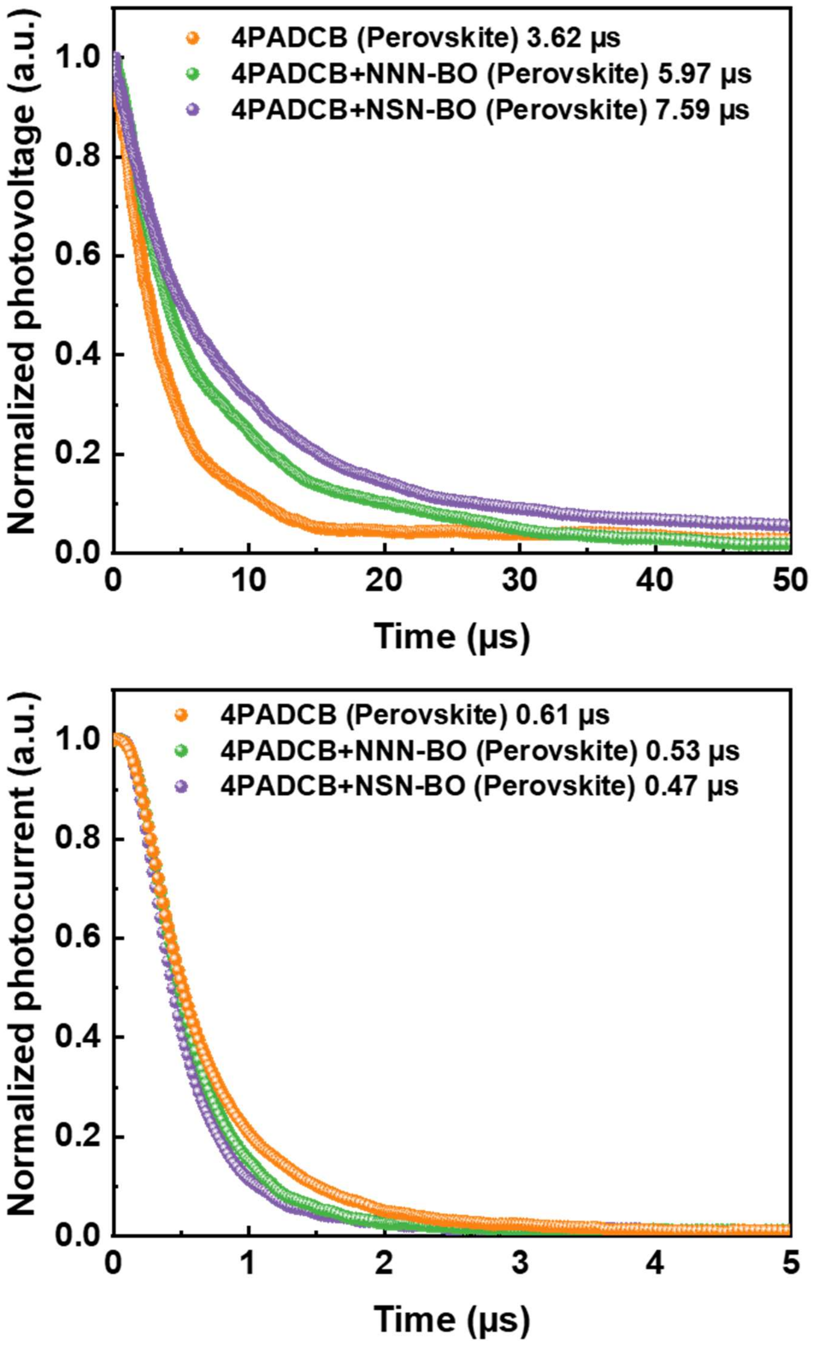

Figure S20. PSCs with or without IAM in terms of transient photovoltage measurements and transient photocurrent measurements.

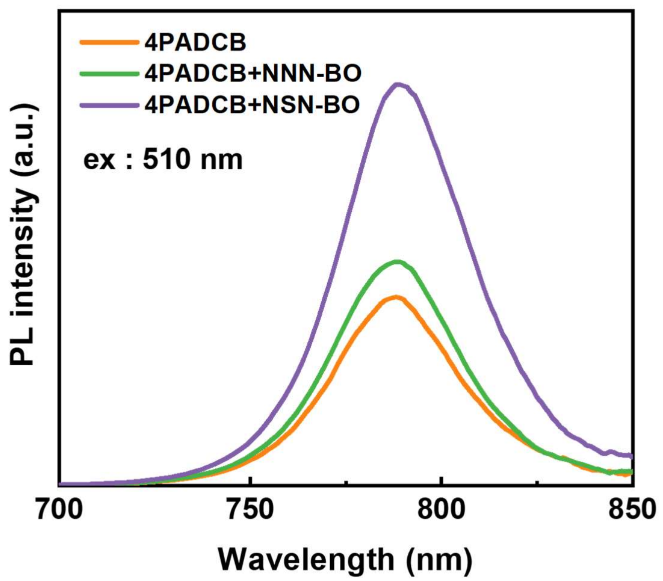

Figure S21. Steady-state PL spectra of PSCs based on 4PDACB and 4PDACB+NNN-BO or NSN-BO as HSL.

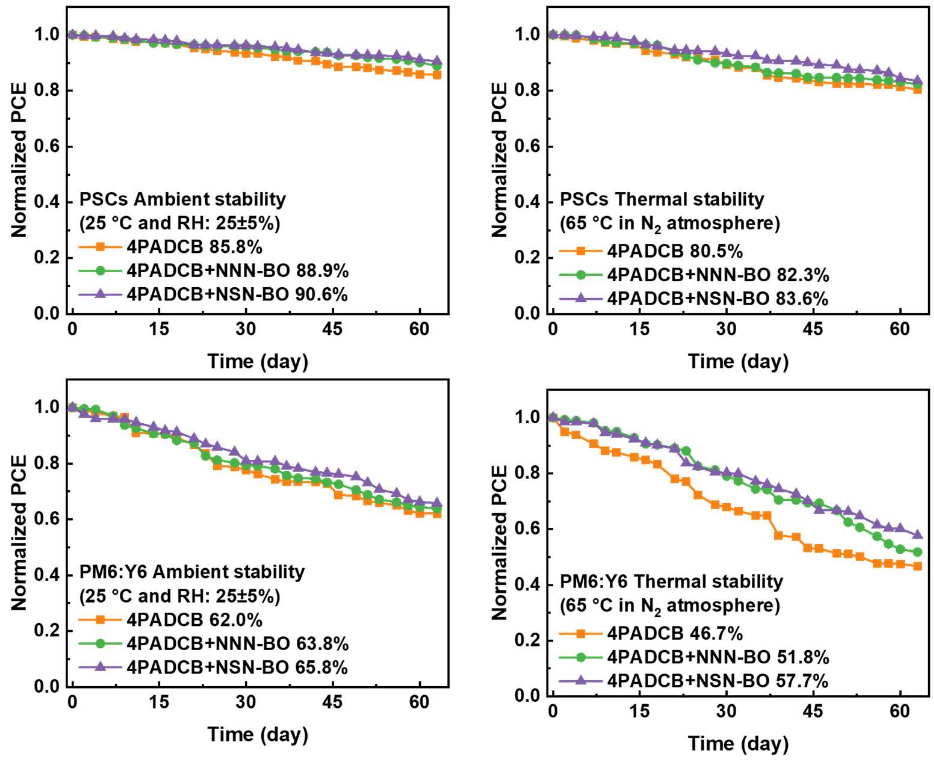

Figure S22. Moisture stability measured under the relative humidity of 25% ± 5%. Thermal stability assessed at 65 °C in an N_2-filled glove box.

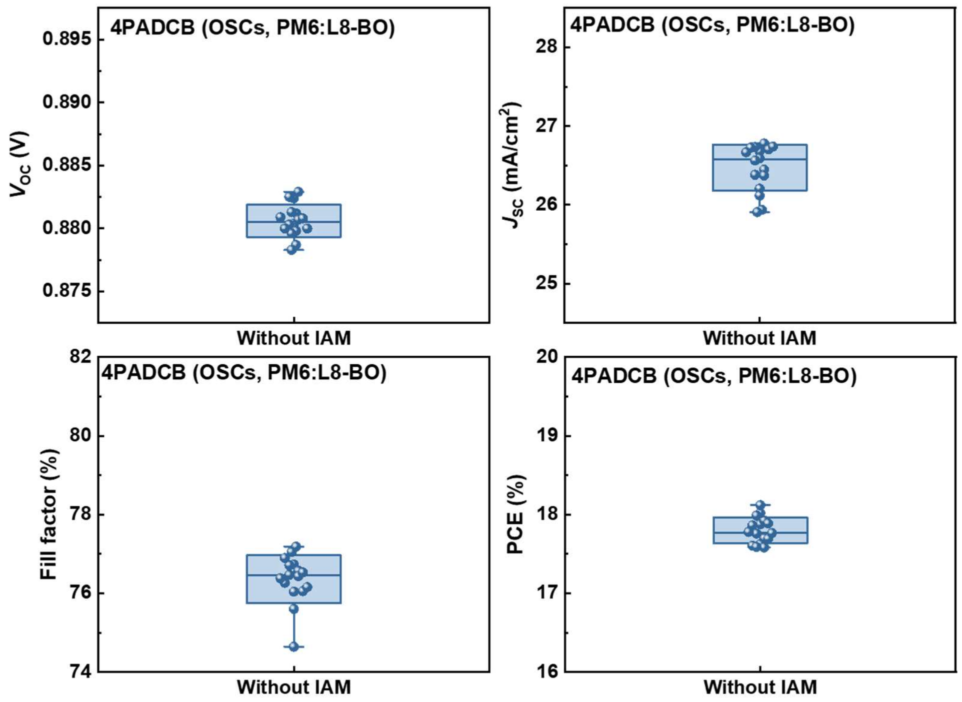

Figure S23. Statistical distribution of photovoltaic parameters for 16 OSCs with 4PADCB as SAM.

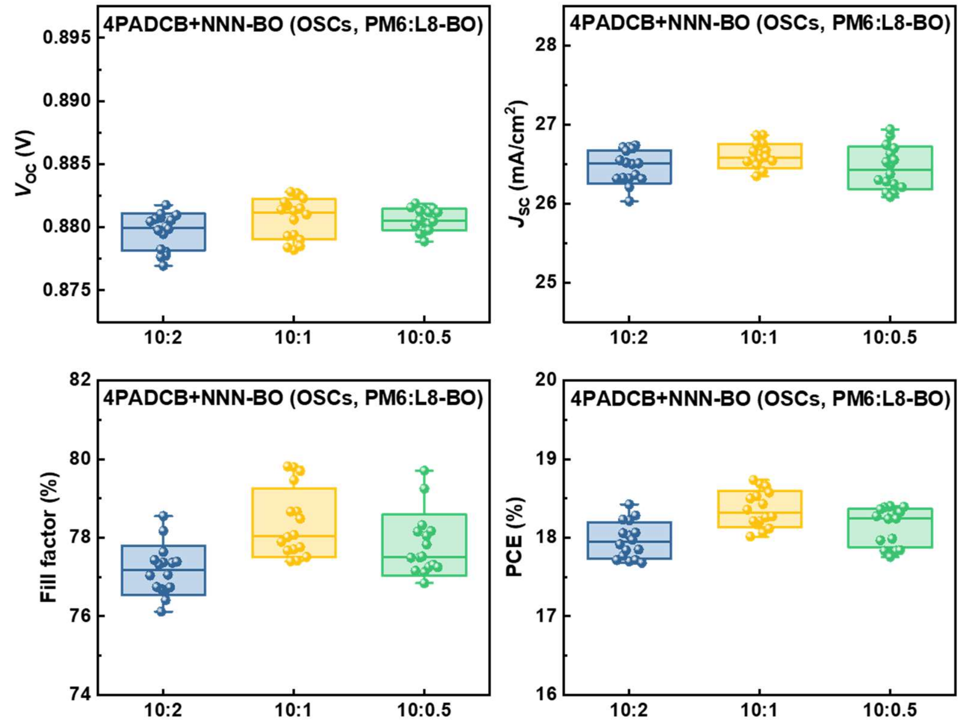

Figure S24. Statistical distribution of photovoltaic parameters for 16 OSCs with different ratios of 4PADCB+NNN-BO as SAM.

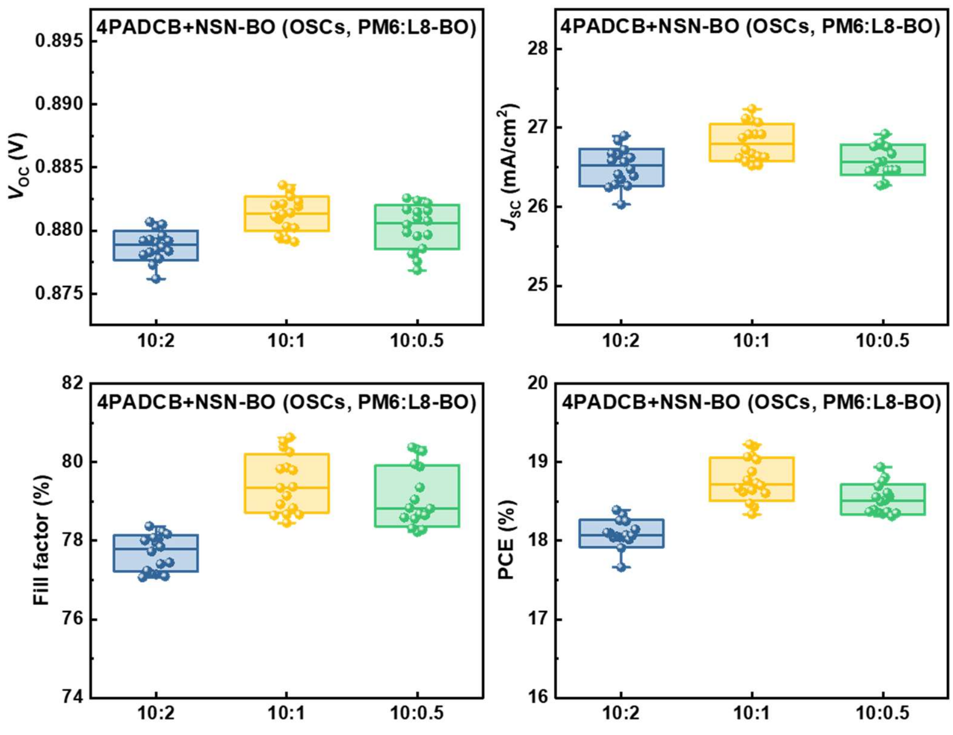

Figure S25. Statistical distribution of photovoltaic parameters for 16 OSCs with different ratios of 4PADCB+NSN-BO as SAM.

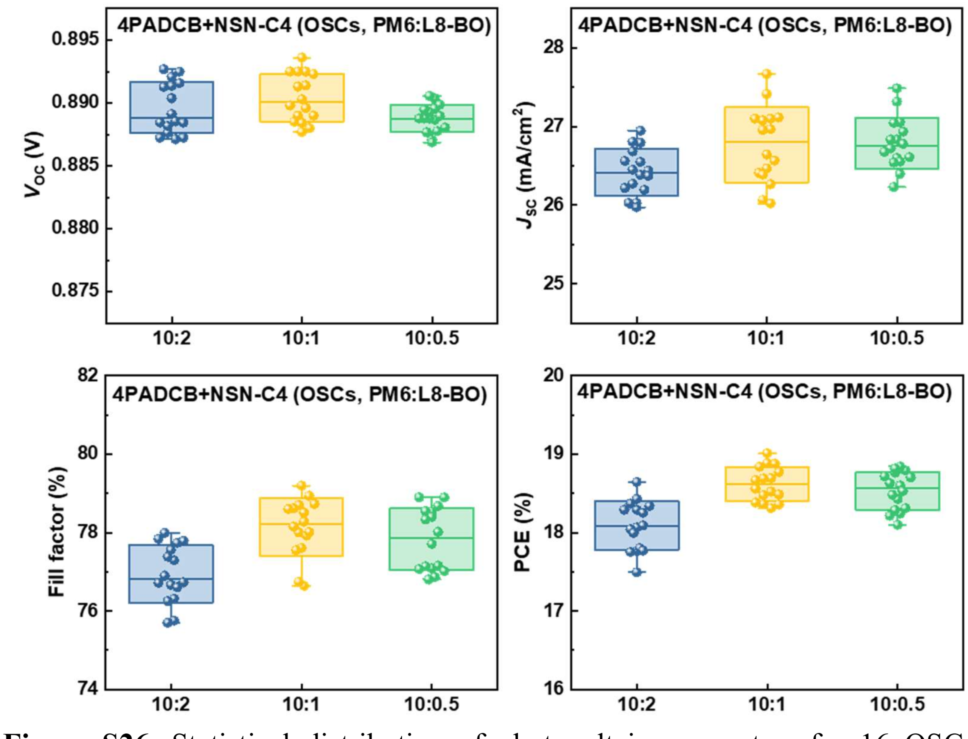

Figure S26. Statistical distribution of photovoltaic parameters for 16 OSCs with different ratios of 4PADCB+NSN-C4 as SAM.

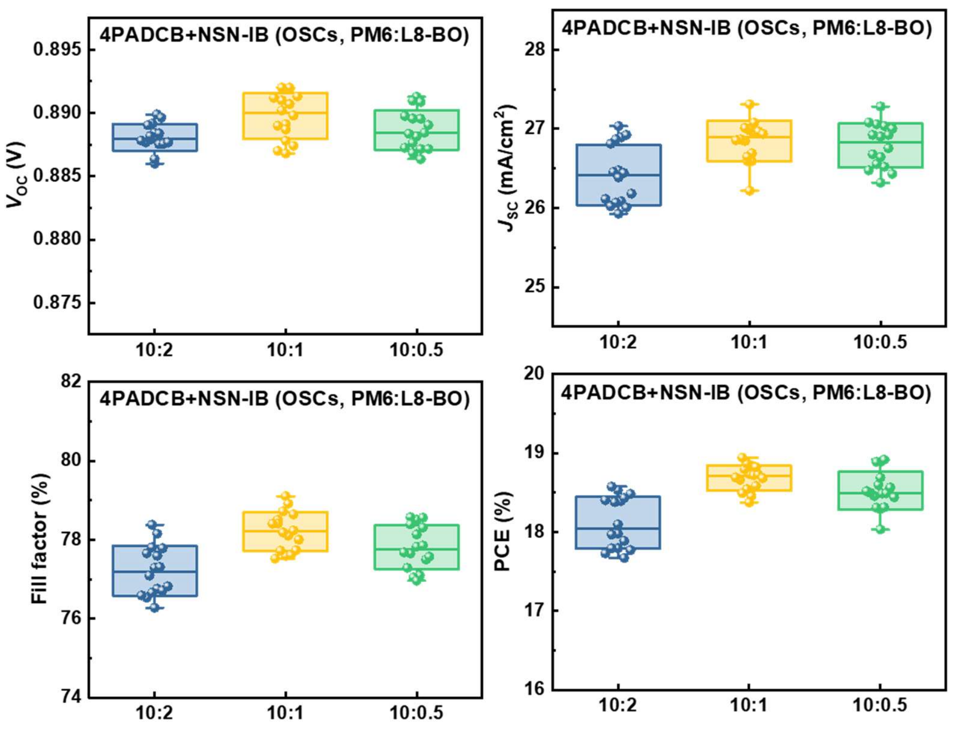

Figure S27. Statistical distribution of photovoltaic parameters for 16 OSCs with different ratios of 4PADCB+NSN-IB as SAM.

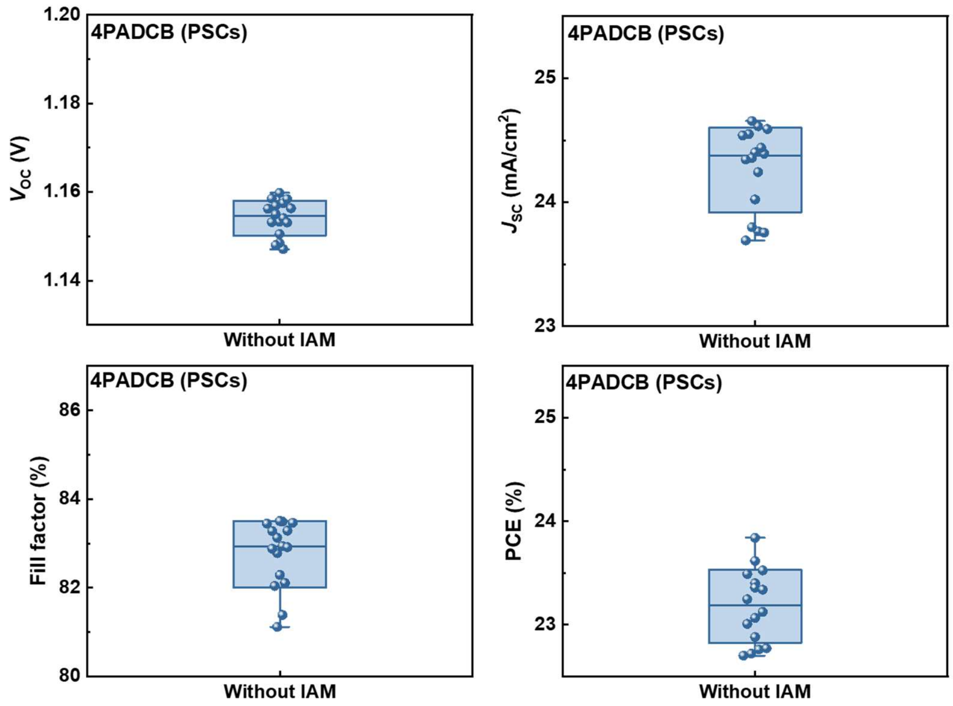

Figure S28. Statistical distribution of photovoltaic parameters for 16 PSCs with 4PADCB as SAM.

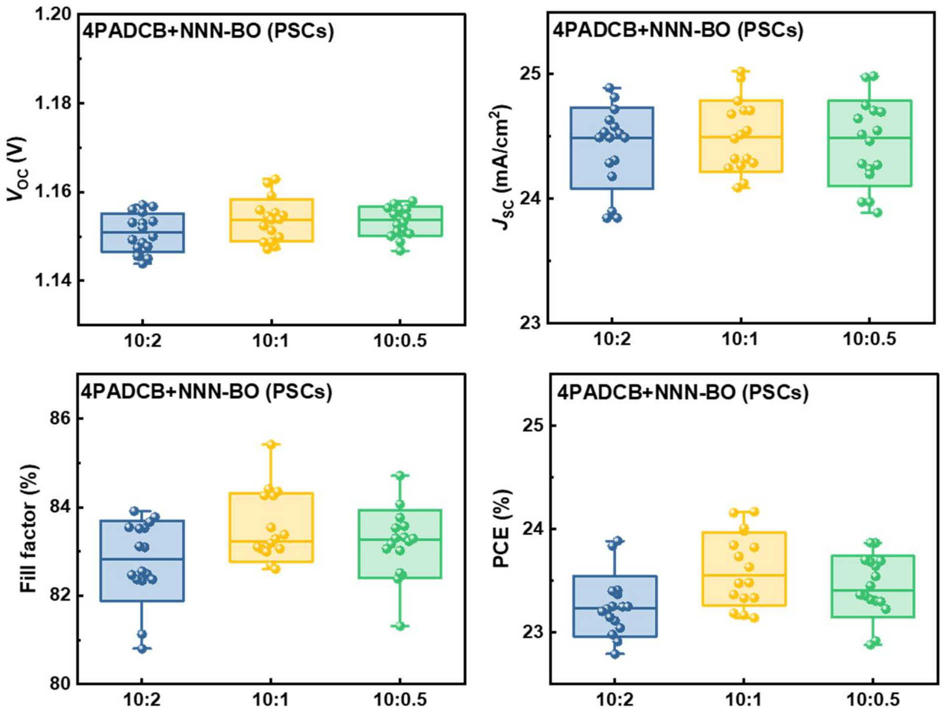

Figure S29. Statistical distribution of photovoltaic parameters for 16 PSCs with different ratios of 4PADCB+NNN-BO as SAM.

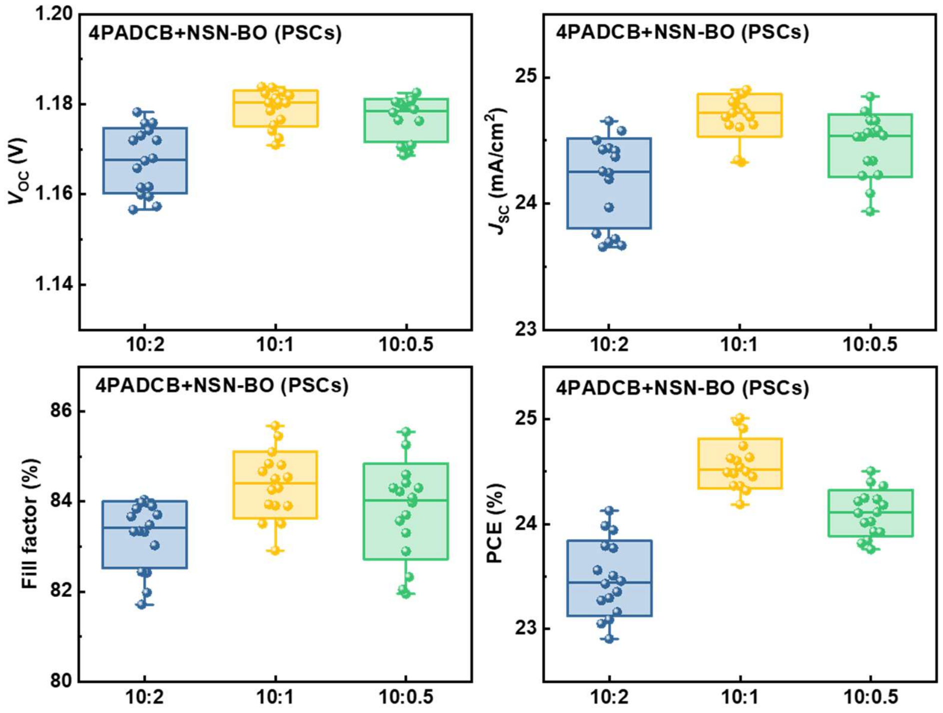

Figure S30. Statistical distribution of photovoltaic parameters for 16 PSCs with different ratios of 4PADCB+NSN-BO as SAM.

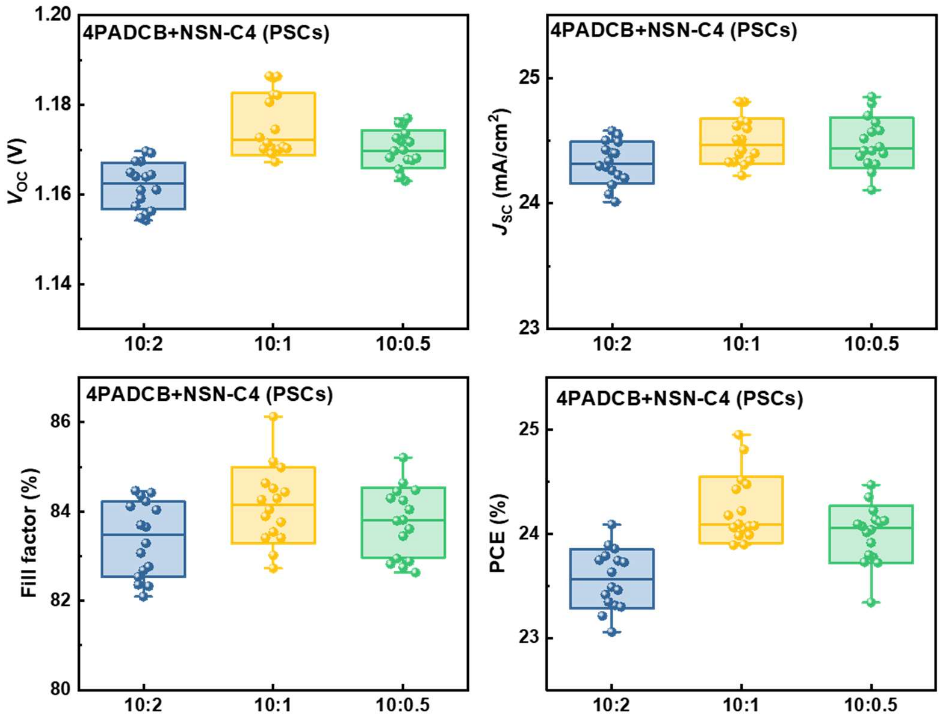

Figure S31. Statistical distribution of photovoltaic parameters for 16 PSCs with different ratios of 4PADCB+NSN-C4 as SAM.

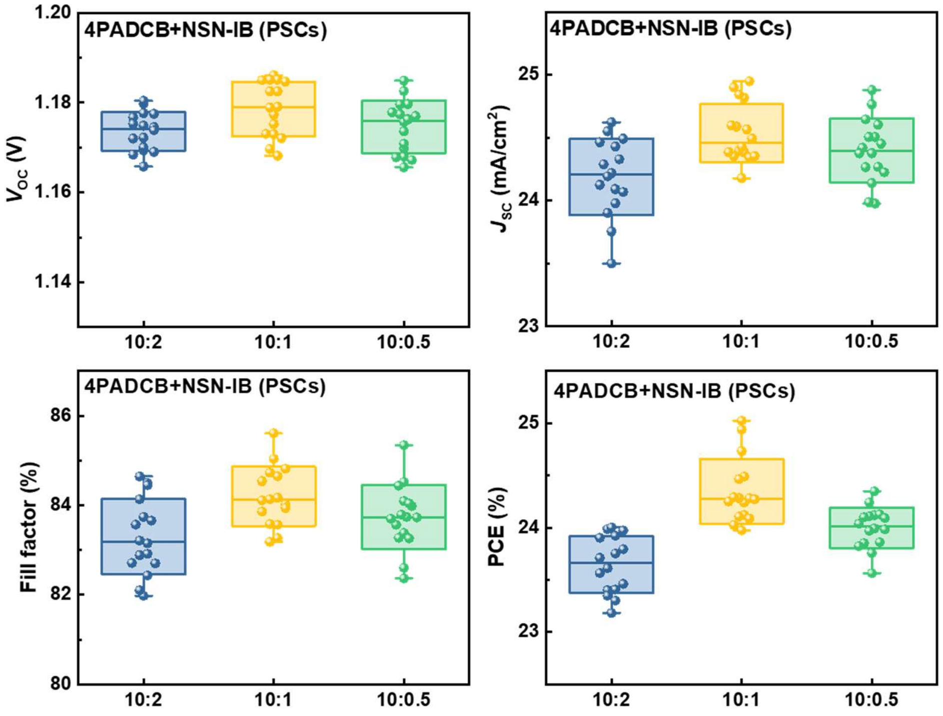

Figure S32. Statistical distribution of photovoltaic parameters for 16 PSCs with different ratios of 4PADCB+NSN-IB as SAM.

Synthesis and Characterization of Compounds:

General Information: Chemicals and solvents were purchased from Sigma-Aldrich, Acros Organics, Combi-Blocks, AK scientific and Alfa Aesar. All commercial reagents were used without purification unless mentioned otherwise. Anhydrous solvents were either obtained by distillation under dry nitrogen gas after drying with CaH_2 or directly purchased as anhydrous. The thin-layer chromatography (TLC) was performed on precoated Merck silica gel 60 F254 alumina plates (0.25 mm), and the spots were visualized by UV lamps (254 nm / 365 nm). Purification with column chromatography was carried out using Geduran® silica gel 60 (0.040-0.063 mm) from Merck. ^{1}H and ^{13}C NMR spectra were recorded on a Bruker AVIII HD 400 spectrometer in $CDCl_3$ and DMSO-d_6. Chemical shifts (δ) were reported in parts per million (ppm) and calibrated using the solvent as the internal standard for ^{1}H ($CHCl_3$: 7.26 ppm, DMSO: 2.50 ppm) and ^{13}C NMR ($CDCl_3$: 77.16 ppm, DMSO-d_6: 39.52 ppm). Coupling constants (*J*) were reported in hertz (Hz), and the splitting patterns were reported as s (singlet), bs (broad singlet), d (doublet), t (triplet), sep (septet), or m (multiplet). High-resolution mass spectra (HRMS) were recorded with a Bruker micrOTOF-QII by electrospray ionization – time of flight (ESI-TOF) experiments at the NTU Consortia of Key Technologies.

4,7-Dibromo-5-nitrobenzo[*c*][1,2,5]thiadiazole (1)

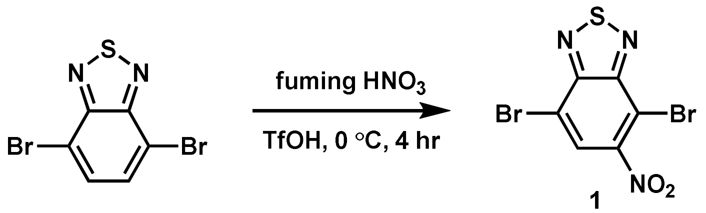

1

Fuming nitric acid (1.42 mL, 34.0 mmol) was added dropwise to trifluoromethanesulfonicacid (15.0 mL, 170.0 mmol) in a 100 mL round bottom flask at 0 °C. The mixture was stirred at 0 °C for 10 min. 4,7-dibromobenzo[*c*][1,2,5]thiadiazole (5.00 g, 17.0 mmol) was added in portions over 30 min and stirred at 0 °C for 2 hours. After completion (determined by TLC monitor), the reaction mixture was poured into ice water (200 mL) slowly. The crude product was filtered, washed with water, and dried. The yellow crude product was purified by flash column chromatography (silica gel, 10% ethyl acetate/hexane) to afford compound **1** as a pale-yellow solid (6.46 g, 93%).

^{1}H NMR ($CDCl_3$, 400 MHz): δ 8.26 (s, 1H).

^{13}C NMR ($CDCl_3$, 101 MHz): δ 152.97, 152.70, 149.91, 127.42, 114.72, 109.11.

HRMS (ESI-TOF): m/z for $C_6Br_2N_3O_2S^-$ $[M-H]^-$, calc'd: 335.8083, found: 335.8074.

5,5'-Dinitro-4,4'-bibenzo[*c*][1,2,5]thiadiazole (2)

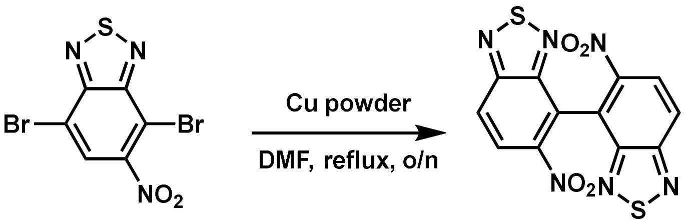

1 2

Compound **1** (5.00 g, 14.75 mmol) and copper powder (2.34 g, 36.87 mmol) were premixed in a 250 mL round bottom flask under N_2 atmosphere. 100 mL of *N*,*N*-dimethylformamide (DMF), degassed with N_2 for 30 min before use, was injected into the reaction flask. The reaction mixture was stirred under reflux overnight. After completion (determined by TLC monitor), the reaction mixture was cooled to room temperature, diluted with dichloromethane (DCM) and extracted with water. The organic layer was dried over Na_2SO_4 and filtered, and the solvent was removed under reduced pressure. The brown crude product was purified by column chromatography (silica gel, 10% ethyl acetate/hexane) to afford compound **2** as a white solid (1.58 g, 59%).

^{1}H NMR (CDCl$_3$, 400 MHz): δ 8.57 (d, J = 9.6 Hz, 2H), 8.30 (d, J = 9.6 Hz, 2H).
^{13}C NMR (CDCl$_3$, 101 MHz): δ 155.47, 152.74, 146.57, 124.69, 124.59, 122.67.
^{1}H NMR (DMSO-*d*$_6$, 400 MHz): δ 8.62 (d, J = 9.6 Hz, 2H), 8.55 (d, J = 9.6 Hz, 2H).
^{13}C NMR (DMSO-*d*$_6$, 101 MHz): δ 154.79, 151.76, 146.62, 124.83, 123.14, 123.07.
HRMS (ESI-TOF): m/z for $C_{12}H_5N_6O_4S_2{}^+$ $[M+H]^+$, calc'd: 360.9808, found: 360.3221.

[4,4'-Bibenzo[*c*][1,2,5]thiadiazole]-5,5'-diamine (3)

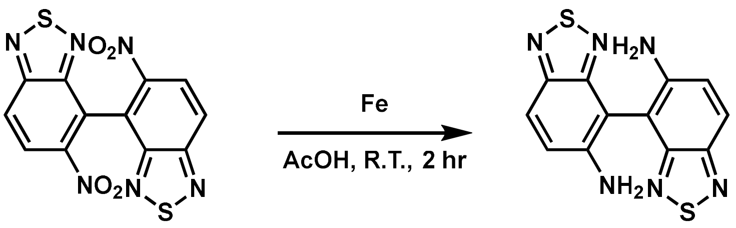

2 3

A 100 mL round bottom flask was charged with compound **2** (0.45 g, 1.27 mmol),

acetic acid (15 mL), and Fe powder (0.85 g, 15.3 mmol). The reaction was stirred at room temperature for 2 hours. After completion (determined by TLC monitor), the reaction mixture was poured into ice water (100 mL) slowly. The crude product was filtered, washed with water, and dried. The yellow crude product was purified by column chromatography (silica gel, ethyl acetate) to afford compound **3** as a white solid (0.38 g, 99%).

^{1}H NMR (CDCl$_3$, 400 MHz): δ 7.92 (d, J = 9.3 Hz, 2H), 7.30 (d, J = 9.3 Hz, 2H), 4.15 (s, 4H).

^{13}C NMR (CDCl$_3$, 101 MHz): δ 155.72, 150.73, 145.81, 124.87, 122.31, 103.63.

^{1}H NMR (DMSO-d_6, 400 MHz): δ 7.82 (d, J = 9.3 Hz, 2H), 7.42 (d, J = 9.3 Hz, 2H), 5.53 (s, 4H).

^{13}C NMR (DMSO-d_6, 101 MHz): δ 156.14, 150.11, 147.29, 126.37, 120.53, 101.77.

HRMS (ESI-TOF): m/z for $C_{12}H_9N_6S_2^+$ [M+H]$^+$, calc'd: 301.0325, found: 301.0325.

6*H*-Bis([1,2,5]thiadiazolo)[3,4-*c*:3',4'-*g*]carbazole (4)

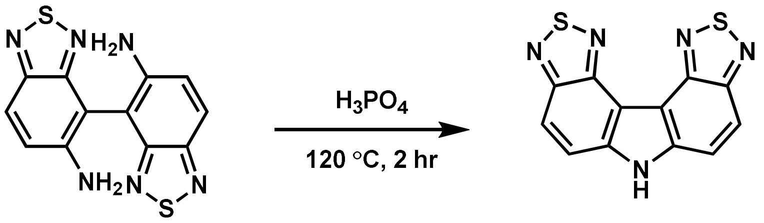

3

4

Into a 100 mL round bottom flask was added compound **3** (0.38 g, 1.28 mmol) and phosphoric acid (15.0 mL). The reaction mixture was stirred at 120 °C for 2 hours. After completion (determined by TLC monitor), the reaction mixture was poured into ice water (100 mL) slowly. The mixture was filtered to collected the filter cake. The brown crude product was washed with DCM/methanol/hexane to afford compound **4** as an orange solid (0.31 g, 83%).

^{1}H NMR (DMSO-d_6, 400 MHz): δ 13.00 (s, 1H), 8.05 (d, J = 9.3 Hz, 2H), 7.97 (d, J = 9.3 Hz, 2H).

^{13}C NMR (DMSO-d_6, 101 MHz): δ 152.65, 149.71, 137.01, 119.51, 117.89, 111.21.

HRMS (ESI-TOF): m/z for $C_{12}H_5N_5NaS_2\ ^+$ [M+Na]$^+$, calc'd: 305.9879, found: 305.9863.

6-(2-Butyloctyl)-6*H*-bis([1,2,5]thiadiazolo)[3,4-*c*:3',4'-*g*]carbazole (NSN-BO)

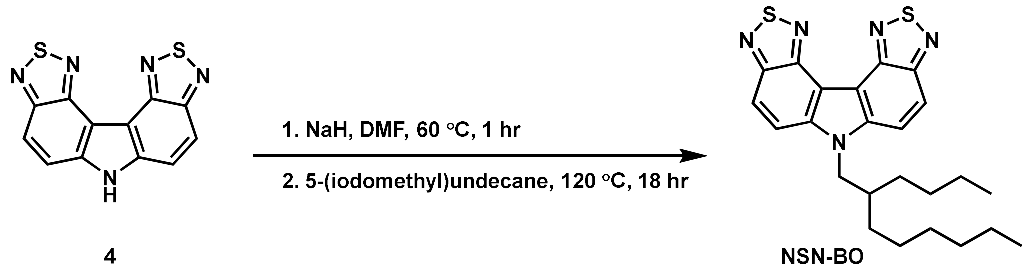

Into an oven-dried 10 mL two-neck round bottom flask was added compound **4** (50.2 mg, 0.18 mmol) and anhydrous DMF (3 mL) under N_2 atmosphere. The reaction mixture was cooled at 0 °C for 10 min, then sodium hydride (7.1 mg, 0.18 mmol) was added slowly. The reaction mixture then allowed to warm to 60 °C for 1 hour. 5-(iodomethyl)undecane (159.8 mg, 0.54 mmol) was then added, and the reaction was heated at 120 °C for 18 hours. The reaction mixture was cooled to room temperature, diluted with DCM, and extracted with water. The organic layer was dried over Na_2SO_4 and filtered, and the solvent was removed under reduced pressure. The brown crude product was purified by column chromatography (silica gel, 0.5% methanol/DCM) to afford **NSN-BO** as a yellow solid (69.1 mg, 75%).

^{1}H NMR ($CDCl_3$, 400 MHz): δ 7.98 (d, J = 9.4 Hz, 2H), 7.81 (d, J = 9.5 Hz, 2H), 4.42 (d, J = 7.6 Hz, 2H), 2.14 – 2.05 (m, 1H), 1.44 – 1.32 (m, 4H), 1.29 – 1.11 (m, 12H), 0.81 (dt, J = 8.2, 7.0 Hz, 6H).

^{13}C NMR ($CDCl_3$, 101 MHz): δ 152.95, 150.35, 137.69, 118.35, 116.39, 112.08, 49.00, 39.42, 31.79, 31.63, 31.51, 29.45, 28.73, 26.52, 22.89, 22.50, 13.95, 13.90.

^{1}H NMR (DMSO-d_6, 400 MHz): δ 8.30 (d, J = 9.5 Hz, 2H), 8.09 (d, J = 9.5 Hz, 2H), 4.70 (d, J = 7.7 Hz, 2H), 2.06 (s, 1H), 1.41 – 1.04 (m, 16H), 0.75 (t, J = 7.1 Hz, 3H), 0.67 (t, J = 6.7 Hz, 3H).

^{13}C NMR (DMSO-d_6, 101 MHz): δ 152.70, 149.67, 137.70, 118.23, 117.93, 111.00, 38.31, 30.91, 30.83, 30.71, 28.87, 27.95, 25.66, 22.41, 21.86, 13.75.

HRMS (ESI-TOF): m/z for $C_{24}H_{30}N_5S_2^+$ $[M+H]^+$, calc'd: 452.1937, found: 452.1918.

6-Butyl-6*H*-bis([1,2,5]thiadiazolo)[3,4-*c*:3',4'-*g*]carbazole (NSN-C4)

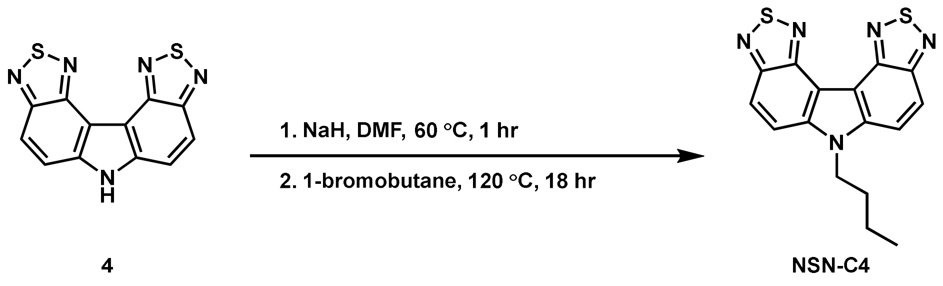

Into an oven-dried 10 mL two-neck round bottom flask was added compound **4** (49.8 mg, 0.18 mmol) and anhydrous DMF (3 mL) under N_2 atmosphere. The reaction mixture was cooled at 0 ˚C for 10 min, then sodium hydride (7.2 mg, 0.18 mmol) was added slowly. The reaction mixture then allowed to warm to 60 °C for 1 hour. 1-bromobutane (0.06 mL, 0.53 mmol) was then added, and the reaction was allowed to heat at 120 °C for 18 hours. The reaction mixture was cooled to room temperature, diluted with DCM, and extracted with water. The organic layer was dried over Na_2SO_4 and filtered, and the solvent was removed under reduced pressure. The brown crude product was purified by column chromatography (silica gel, 0.5% methanol/DCM) to afford **NSN-C4** as a yellow solid (40.1 mg, 68%).

^{1}H NMR ($CDCl_3$, 400 MHz): δ 8.01 (d, J = 9.4 Hz, 2H), 7.87 (d, J = 9.4 Hz, 2H), 4.60 (t, J = 7.3 Hz, 2H), 2.03 – 1.91 (m, 2H), 1.52 – 1.38 (m, 2H), 0.99 (t, J = 7.4 Hz, 3H).

^{13}C NMR ($CDCl_3$, 101 MHz): δ 152.95, 150.35, 137.28, 118.46, 116.12, 112.11, 44.36, 32.55, 20.36, 13.75.

HRMS (ESI-TOF): m/z for $C_{16}H_{14}N_5S_2^+$ $[M+H]^+$, calc'd: 340.0685, found: 340.0686.

6-Isobutyl-6*H*-bis([1,2,5]thiadiazolo)[3,4-*c*:3',4'-*g*]carbazole (NSN-IB)

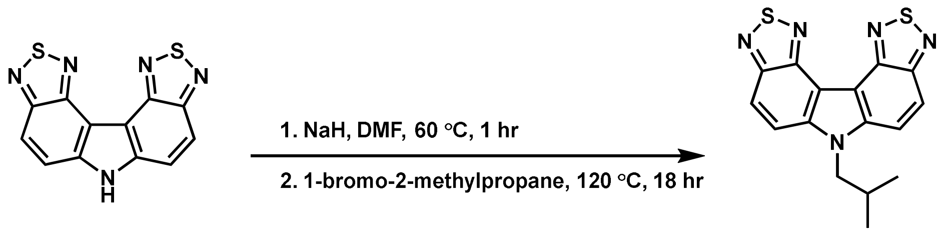

Into an oven-dried 10 mL two-neck round bottom flask was added compound **4** (50.4 mg, 0.18 mmol) and anhydrous DMF (3 mL) under N_2 atmosphere. The reaction mixture was cooled at 0 °C for 10 min, then sodium hydride (7.0 mg, 0.18 mmol) was added slowly. The reaction mixture then allowed to warm to 60 °C for 1 hour. 1-bromo-2-methylpropane (0.06 mL, 0.53 mmol) was then added, and the reaction was heated at 120 °C for 18 hours. The reaction mixture was cooled to room temperature, diluted with DCM, and extracted with water. The organic layer was dried over Na_2SO_4 and filtered, and the solvent was removed under reduced pressure. The brown crude product was purified by column chromatography (silica gel, 0.5% methanol/DCM) to afford **NSN-IB** as a yellow solid (35.1 mg, 59%).

^{1}H NMR ($CDCl_3$, 400 MHz): δ 8.01 (d, J = 9.5 Hz, 2H), 7.87 (d, J = 9.5 Hz, 2H), 4.41 (d, J = 7.6 Hz, 2H), 2.41 (sep, J = 7.0 Hz, 1H), 1.03 (d, J = 6.6 Hz, 6H).

^{13}C NMR ($CDCl_3$, 101 MHz): δ 152.95, 150.36, 137.64, 118.40, 116.46, 112.10, 51.83, 30.15, 20.39.

HRMS (ESI-TOF): m/z for $C_{16}H_{14}N_5S_2^+$ $[M+H]^+$, calc'd: 340.0685, found: 340.0684.

3,6-Dibromobenzene-1,2-diamine (5)

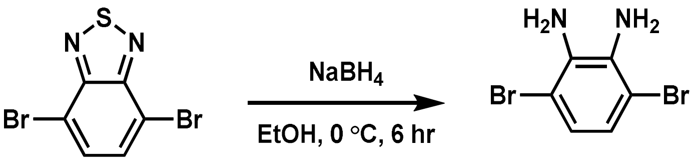

5

Into a 500 mL round bottom flask was added 4,7-dibromobenzo[*c*][1,2,5]thiadiazole (5.89 g, 20.0 mmol) and EtOH (200 mL). The mixture was cooled to 0 °C for 10 min, then sodium borohydride (14.40 g, 380.0 mmol) was added in portions over 15 min and stirred at 0 °C for 6 hours. After completion (determined by TLC monitor), ethanol was mostly removed under reduced pressure, and the reaction mixture was quenched by addition of ice water. The solution was filtered to collect the filter cake. The orange crude product was purified by flash column chromatography (silica gel, 10% ethyl acetate/hexane) to afford compound **5** as a white solid (5.11 g, 95%).

^{1}H NMR ($CDCl_3$, 400 MHz): δ 6.84 (s, 2H), 3.88 – 3.63 (bs, 4H).

^{13}C NMR ($CDCl_3$, 101 MHz): δ 133.71, 123.24, 109.68.

HRMS (ESI-TOF): m/z for $C_6H_7Br_2N_2^+$ $[M+H]^+$, calc'd: 264.8970, found: 264.8964.

4,7-Dibromo-2-methyl-2*H*-benzo[*d*][1,2,3]triazole (6)

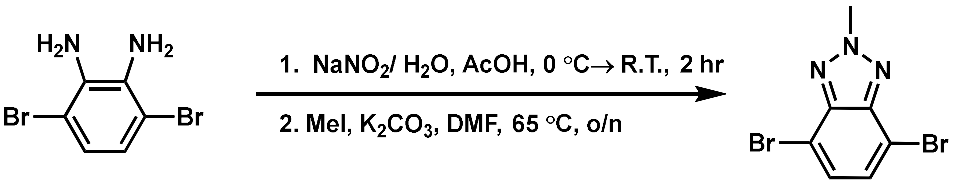

5 6

A 100 mL round bottom flask containing compound **5** (2.33 g, 8.71 mmol) and acetic acid (17 mL) was cooled to 0 °C for 10 min, then a solution of $NaNO_2$ (0.66 g, 0.48 mmol) in 17 mL water was added dropwise. After the addition, the reaction mixture was stirred for 2 hours at room temperature. After completion (determined by TLC monitor), the reaction mixture was cooled to 0 °C and the precipitate was collected by filtration. The orange precipitate was washed with water on the filter paper and dried *in vacuo* to afford an orange crude product. The crude was added into an oven-dried 50

mL two-neck round bottom flask under N_2 atmosphere, along with potassium carbonate (2.33 g, 16.91 mmol) and 17 mL of DMF, degassed with N_2 for 30 min before used. The reaction mixture was stirred at 60 °C for 30 min. Iodomethane (2.11 mL, 33.8 mmol) was then added to a reaction mixture in one portion. The reaction mixture was stirred at 60 °C for overnight. After completion (determined by TLC monitor), the reaction mixture was cooled to room temperature, diluted with DCM, and extracted with water. The organic layer was dried over Na_2SO_4 and filtered, and the solvent was removed under reduced pressure. The orange crude product was purified by column chromatography (silica gel, 40% DCM/hexane) to afford compound **6** as a yellow solid (0.91 g, 37%).

^{1}H NMR ($CDCl_3$, 400 MHz): δ 7.42 (s, 2H), 4.57 (s, 3H).

^{13}C NMR ($CDCl_3$, 101 MHz): δ 143.89, 129.65, 109.79, 43.89.

HRMS (ESI-TOF): m/z for $C_7H_6Br_2N_3^+$ $[M+H]^+$, calc'd: 289.8923, found: 289.8906.

4,7-Dibromo-2-methyl-5-nitro-2*H*-benzo[*d*][1,2,3]triazole (7)

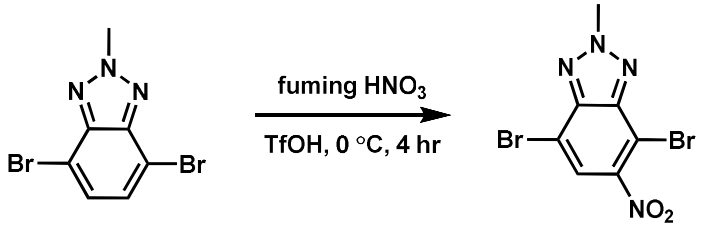

6 7

Into a 50 mL round bottom flask was added compound **6** (1.82 g, 6.25 mmol) and trifluoromethanesulfonicacid (10.0 mL), then the reaction mixture was stirred at 0 ℃ for 10 min. Fuming nitric acid (0.52 mL, 12.5 mmol) was added dropwise, then followed by stirring at 0 °C for another 4 hours. After completion (determined by TLC monitor), the reaction mixture was poured into ice water (100 mL) slowly. The crude product was filtered, washed with water, and dried. The yellow crude product was purified by flash column chromatography (silica gel, 20.0% ethyl acetate/hexane) to afford compound **7** as a pale-yellow solid (2.00 g, 95%).

^{1}H NMR ($CDCl_3$, 400 MHz): δ 8.11 (s, 1H), 4.64 (s, 3H).

^{13}C NMR ($CDCl_3$, 101 MHz): δ 147.33, 144.07, 143.68, 125.42, 110.81, 106.33, 44.57.

HRMS (ESI-TOF): m/z for $C_7H_5Br_2N_4O_2^+$ $[M+H]^+$, calc'd: 334.8774, found: 334.8757.

2,2'-Dimethyl-5,5'-dinitro-2*H*,2'*H*-4,4'-bibenzo[*d*][1,2,3]triazole (8)

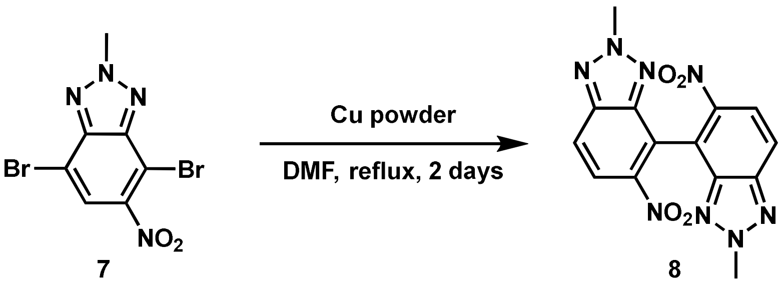

Compound **7** (2.50 g, 7.44 mmol) and copper powder (1.18 g, 18.6 mmol) were premixed in a 100 mL round bottom flask under N_2 atmosphere. 25 mL of DMF, degassed with N_2 for 30 min before use, was injected into it, the reaction was stirred under reflux for 2 days. After completion (determined by TLC monitor), the reaction mixture was cooled to room temperature, diluted with DCM and extracted with water. The organic layer was dried over Na_2SO_4 and filtered, and the solvent was removed under reduced pressure. The brown crude product was purified by column chromatography (silica gel, 50% ethyl acetate/hexane) to afford compound **8** as a white solid (0.84 g, 64%).

^{1}H NMR ($CDCl_3$, 400 MHz): δ 8.35 (d, J = 9.4 Hz, 2H), 8.09 (d, J = 9.3 Hz, 2H), 4.42 (s, 6H).

^{13}C NMR ($CDCl_3$, 101 MHz): δ 145.76, 144.27, 142.90, 122.85, 122.42, 119.27, 44.06.

HRMS (ESI-TOF): m/z for $C_{14}H_{11}N_8O_4^+$ $[M+H]^+$, calc'd: 355.0898, found: 355.0869.

2,2'-Dimethyl-2*H*,2'*H*-[4,4'-bibenzo[*d*][1,2,3]triazole]-5,5'-diamine (9)

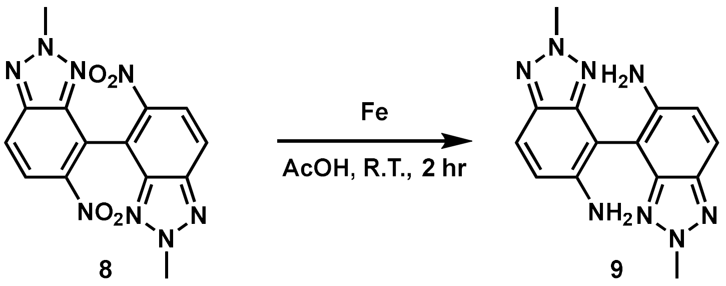

A 100 mL round bottom flask was charge with compound **8** (0.61 g, 1.72 mmol), acetic acid (20 mL), and iron dust (1.15 g, 20.59 mmol). The reaction was stirred at room temperature for 2 hours. After completion (determined by TLC monitor), the

reaction mixture was poured into ice water (100 mL) slowly. The crude product was filtered, washed with water, and dried. The orange crude product was purified by column chromatography (silica gel, 10% methanol/ethyl acetate) to afford compound **9** as a yellow solid (0.49 g, 98%).

^{1}H NMR ($CDCl_3$, 400 MHz): δ 7.73 (d, J = 9.0 Hz, 2H), 7.02 (d, J = 9.0 Hz, 2H), 4.35 (s, 6H), 3.87 (bs, 4H).

^{13}C NMR ($CDCl_3$, 101 MHz): δ 145.04, 142.92, 140.23, 120.91, 118.75, 102.10, 42.92.

^{1}H NMR (DMSO-d_6, 400 MHz): δ 7.64 (d, J = 9.0 Hz, 2H), 7.09 (d, J = 9.0 Hz, 2H), 4.77 (s, 4H), 4.23 (s, 6H).

^{13}C NMR (DMSO-d_6, 101 MHz): δ 145.24, 143.76, 138.86, 120.90, 117.13, 100.73, 42.38.

HRMS (ESI-TOF): m/z for $C_{14}H_{15}N_8^+$ $[M+H]^+$, calc'd: 295.1414, found: 295.1419.

2,10-Dimethyl-6,10-dihydro-2*H*-bis([1,2,3]triazolo)[4,5-*c*:4',5'-*g*]carbazole (10)

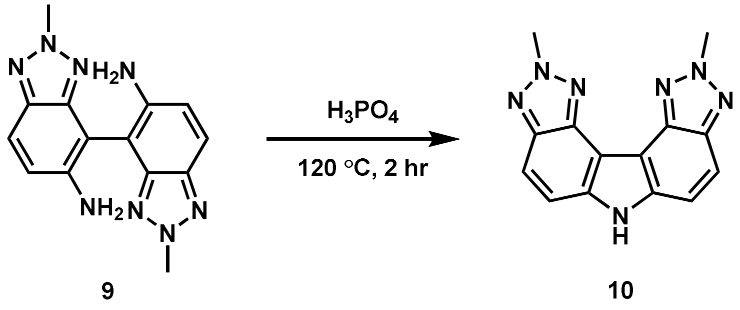

Into a 100 mL round bottom flask was added compound **9** (0.45 g, 1.52 mmol) and phosphoric acid (20.0 mL). The reaction mixture was stirred at 120 °C for 2 hours. After completion (determined by TLC monitor), the reaction mixture was poured into ice water (100 mL) slowly. The mixture was filtered to collect the filter cake. The orange crude product was purified by column chromatography (silica gel, 2% methanol/DCM), followed by recrystallization with DCM/hexane to afford compound **10** as orange needle-shaped crystal (0.20 g, 47%).

^{1}H NMR (DMSO-d_6, 400 MHz): δ 12.45 (s, 1H), 7.83 (d, J = 9.1 Hz, 2H), 7.76 (d, J = 9.1 Hz, 2H), 4.58 (s, 6H).

^{13}C NMR (DMSO-d_6, 101 MHz): δ 140.76, 139.22, 135.71, 115.05, 114.20, 107.57, 43.04.

HRMS (ESI-TOF): m/z for $C_{14}H_{12}N_7^+$ $[M+H]^+$, calc'd: 278.1149, found: 278.1154.

6-(2-Butyloctyl)-2,10-dimethyl-6,10-dihydro-2*H*-bis([1,2,3]triazolo)[4,5-*c*:4',5'-*g*]carbazole (NNN-BO)

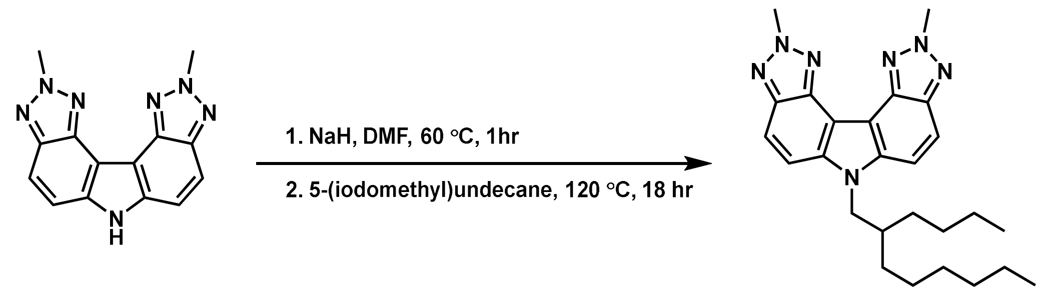

10 **NNN-BO**

Into an oven-dried 10 mL two-neck round bottom flask was added compound **12** (50.0 mg, 0.18 mmol) and anhydrous DMF (3 mL) under N_2 atmosphere. The reaction mixture was cooled at 0 °C for 10 min, then sodium hydride (7.2 mg, 0.18 mmol) was added slowly. The reaction mixture then allowed to warm to 60 °C for 1 hour. 5-(iodomethyl)undecane (160.2 mg, 0.54 mmol) was then added, and the reaction was allowed to heat at 120 °C for 18 hours. The reaction mixture was cooled to room temperature, diluted with DCM, and extracted with water. The organic layer was dried over Na_2SO_4 and filtered, and the solvent was removed under reduced pressure. The brown crude product was purified by column chromatography (silica gel, dichloromethane) to afford **NNN-BO** as a white solid (64.8 mg, 81%).

^{1}H NMR ($CDCl_3$, 400 MHz): δ 7.87 (d, *J* = 9.2 Hz, 2H), 7.65 (d, *J* = 9.3 Hz, 2H), 4.69 (s, 6H), 4.42 (d, *J* = 7.6 Hz, 2H), 2.19 – 2.12 (m, 1H), 1.41 – 1.29 (m, 6H), 1.26 – 1.13 (m, 10H), 0.80 (dt, *J* = 7.1, 1.3 Hz, 6H).

^{13}C NMR ($CDCl_3$, 101 MHz): δ 141.45, 139.90, 136.61, 114.31, 112.85, 108.05, 48.68, 43.25, 39.01, 31.90, 31.63, 31.60, 29.48, 28.76, 26.52, 22.90, 22.51, 13.97, 13.89.

^{1}H NMR (DMSO-*d*$_6$, 400 MHz): δ 7.90 (s, 4H), 4.61 (s, 6H), 2.06 (s, 1H), 1.28 (m, 6H), 1.18 – 1.00 (m, 10H), 0.70 (two overlapping triplets, J = 7.0 Hz, 6H).

^{13}C NMR (DMSO-*d*$_6$, 101 MHz): δ 140.66, 139.08, 136.16, 114.05, 113.42, 107.16, 47.75, 43.02, 38.22, 31.02, 30.95, 30.79, 28.88, 28.06, 25.74, 22.41, 21.91, 13.82, 13.74.

HRMS (ESI-TOF): m/z for $C_{26}H_{36}N_7^+$ $[M+H]^+$, calc'd: 446.3027, found: 446.3022.

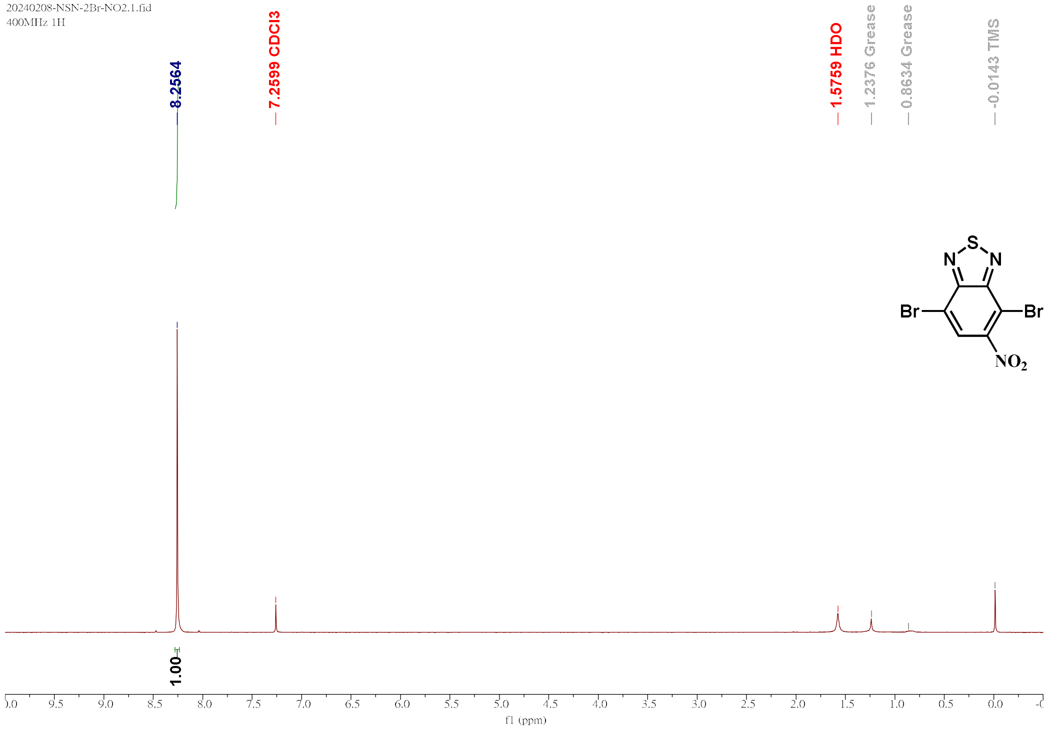

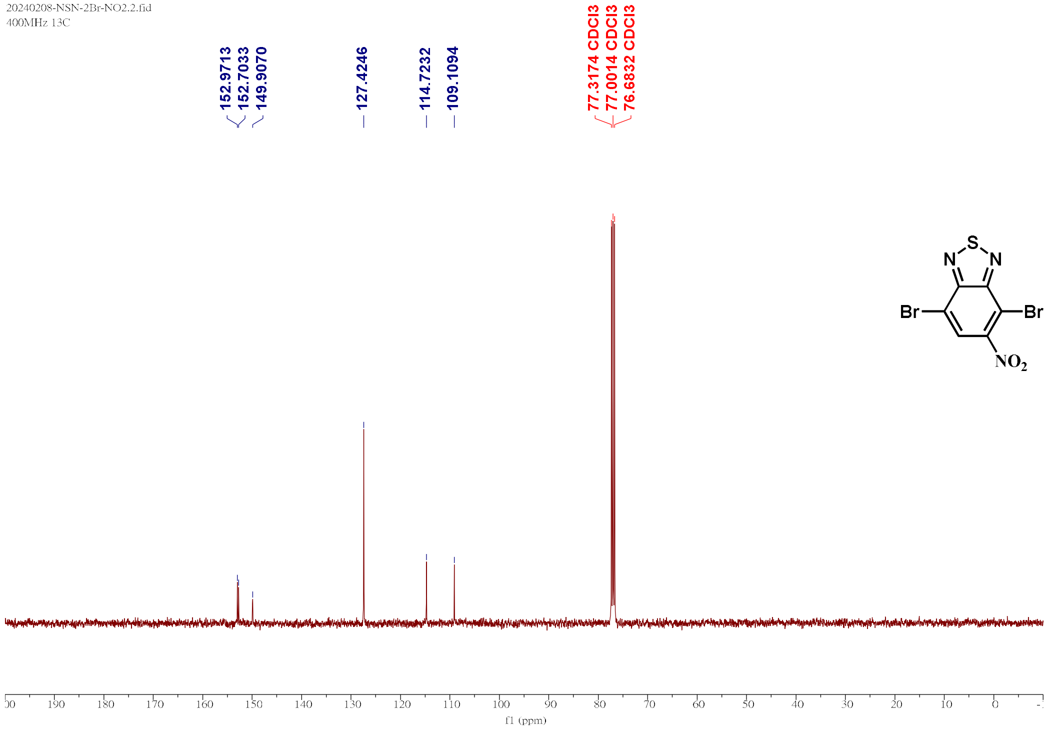

Figure S33. ^{1}H and ^{13}C NMR spectra of compound **1** ($CDCl_3$).

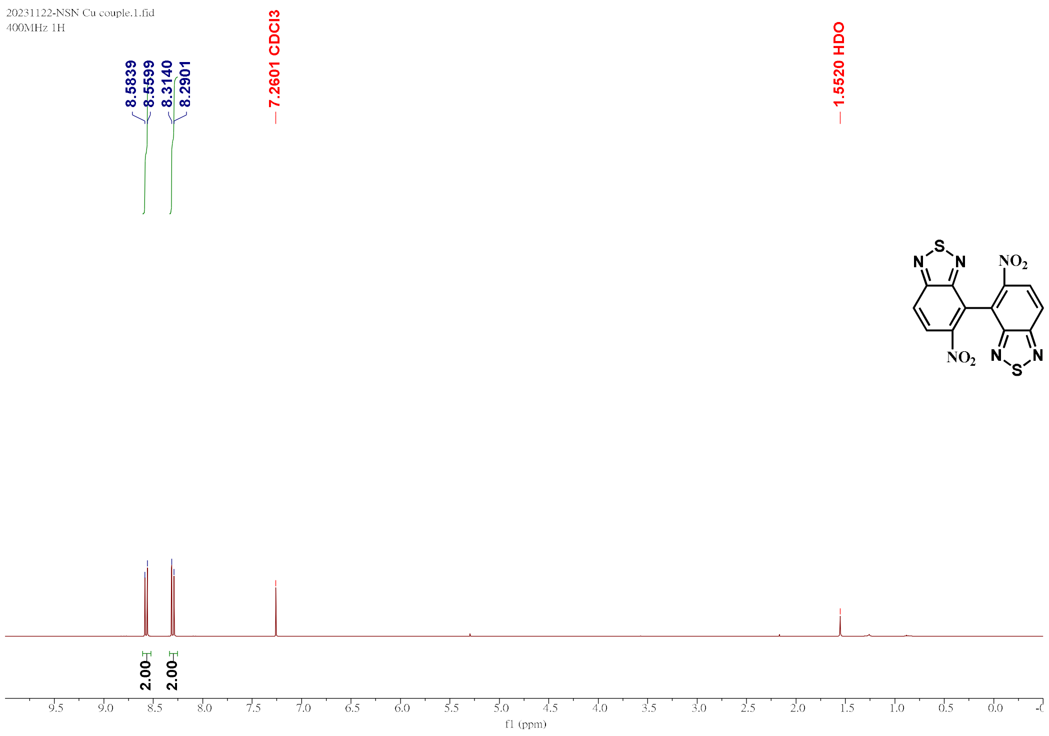

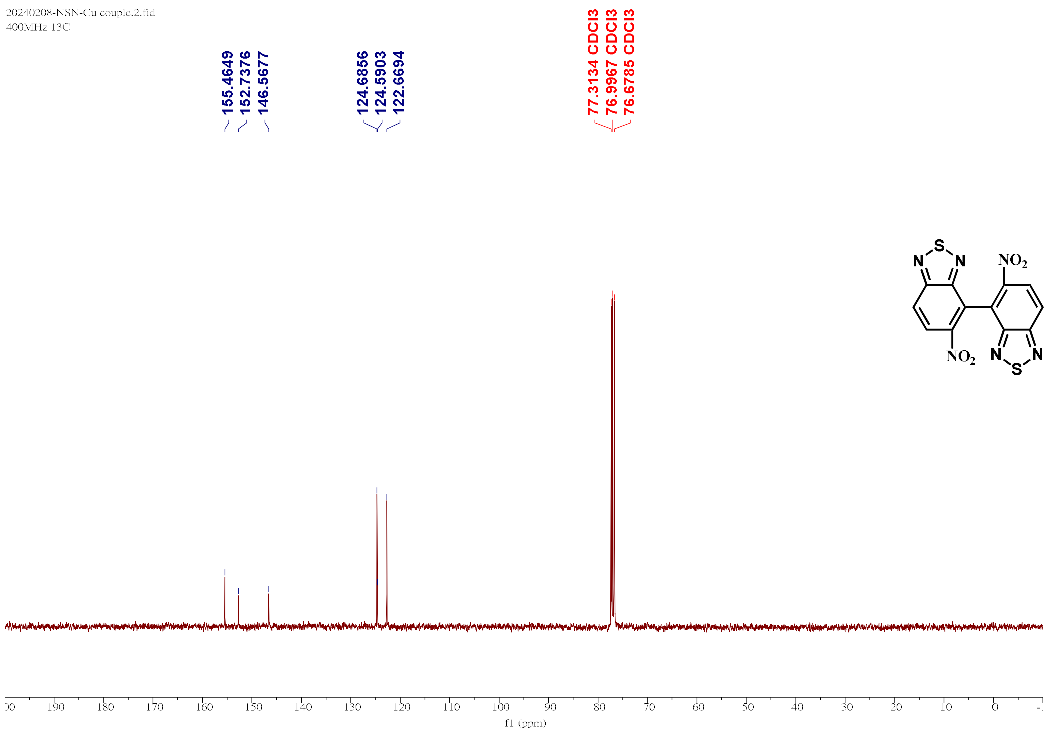

Figure S34. ^{1}H and ^{13}C NMR spectra of compound **2** ($CDCl_3$).

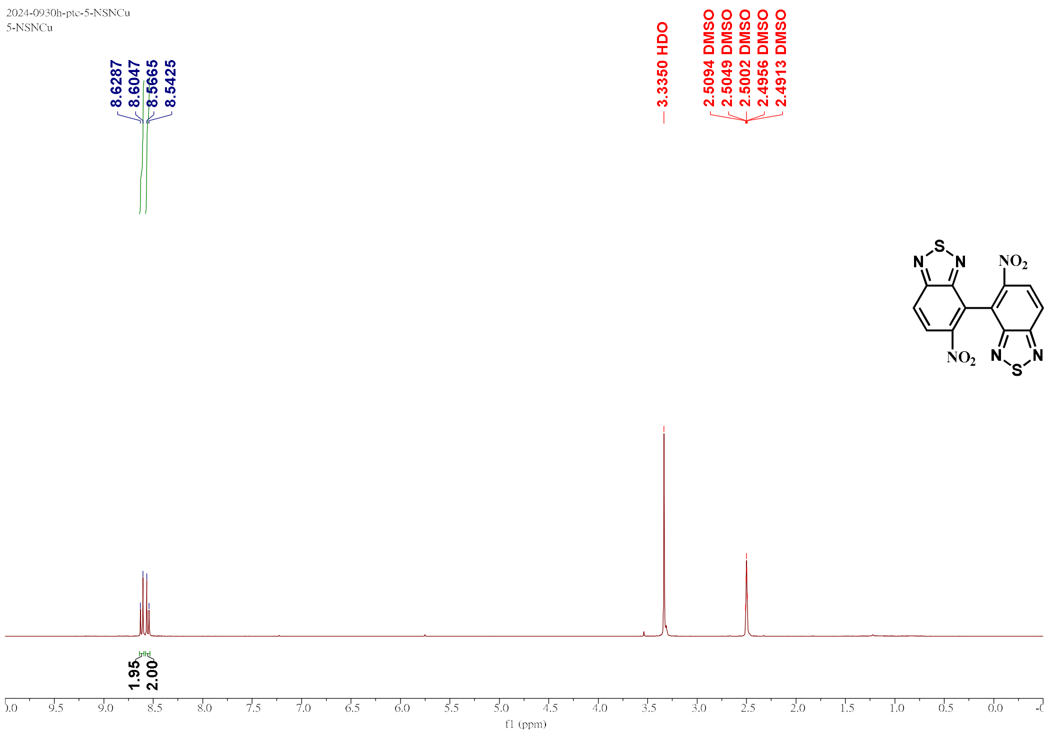

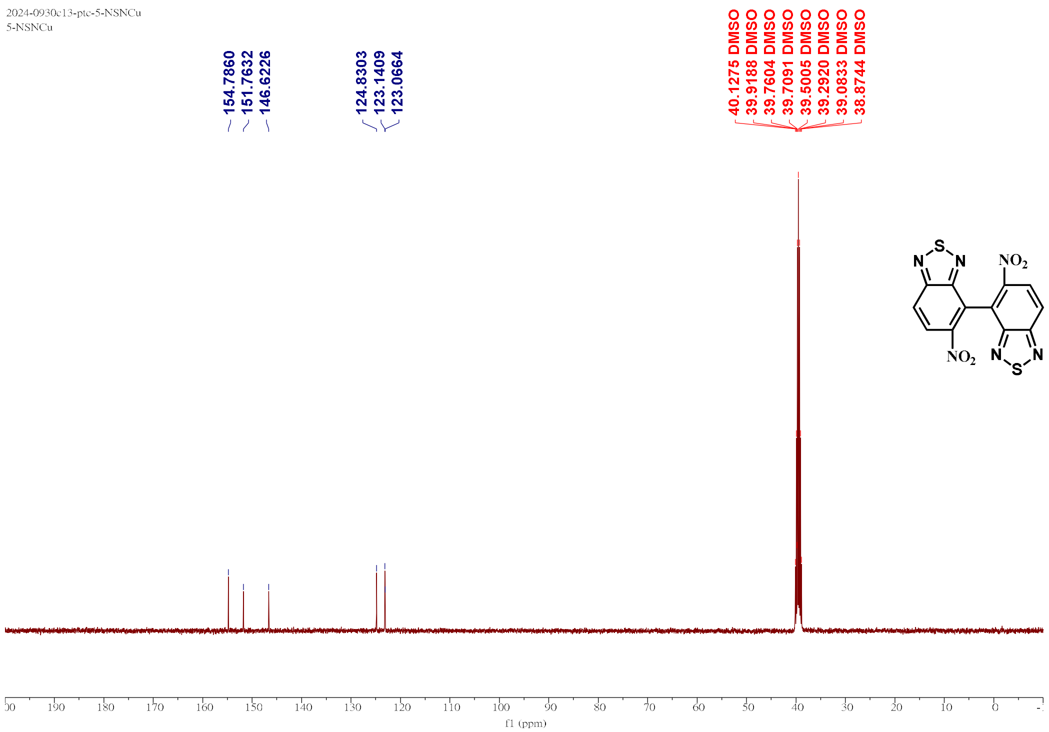

Figure S35. ^{1}H and ^{13}C NMR spectra of compound **2** (DMSO-*d*$_6$).

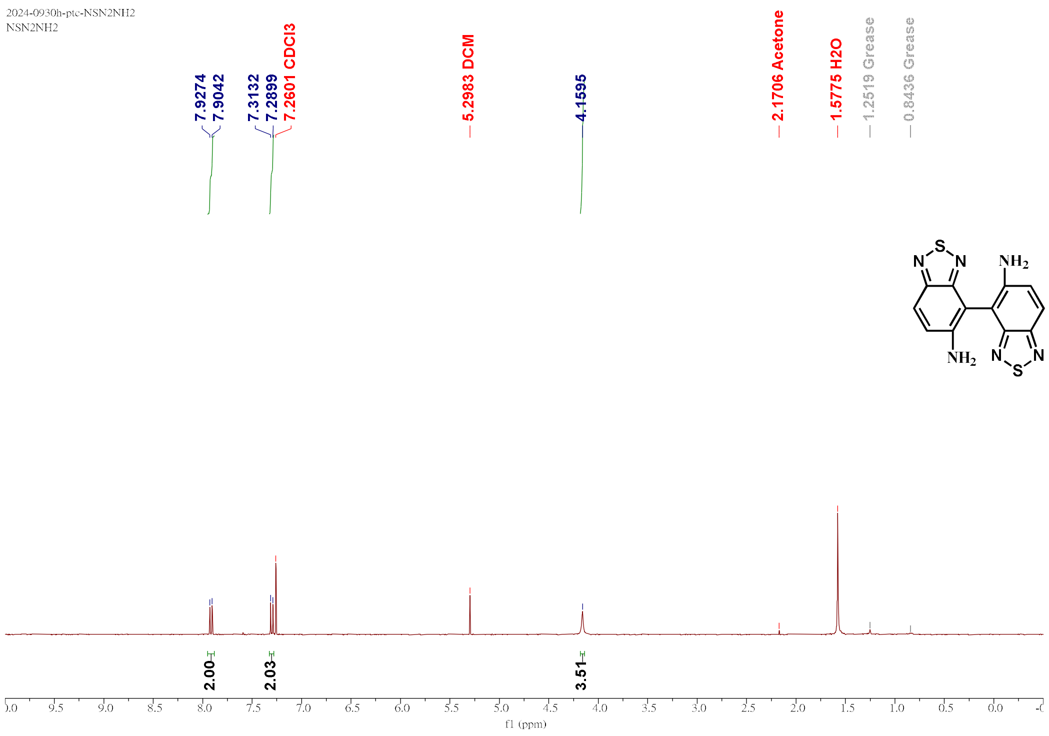

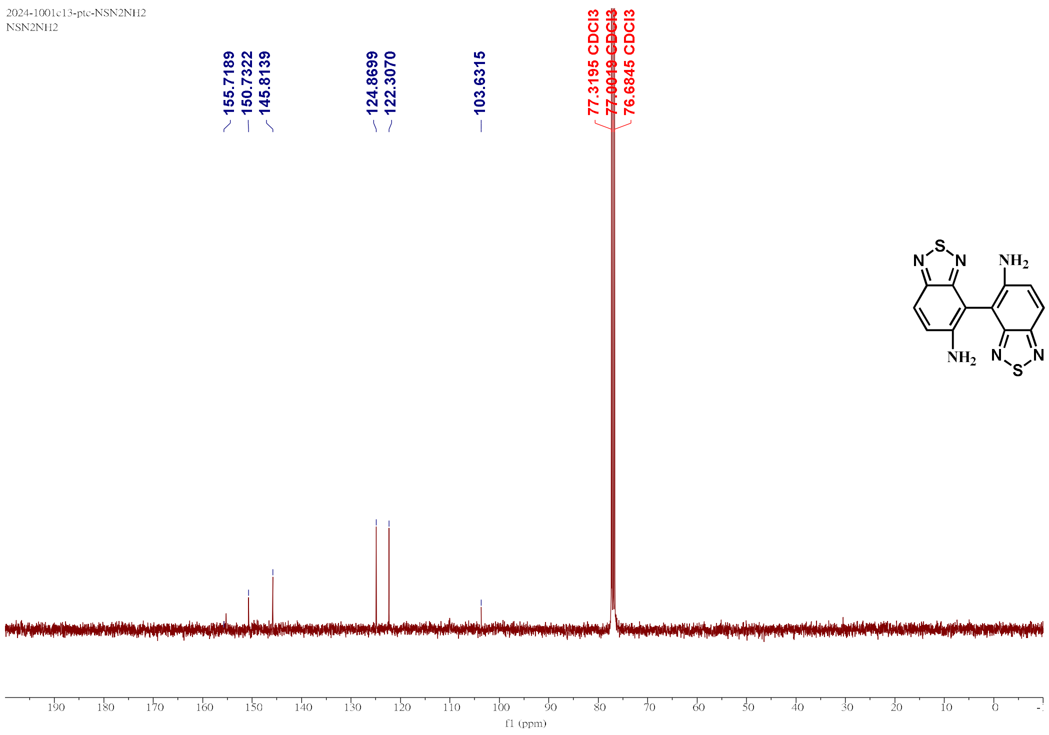

Figure S36. ^{1}H and ^{13}C NMR spectra of compound **3** ($CDCl_3$).

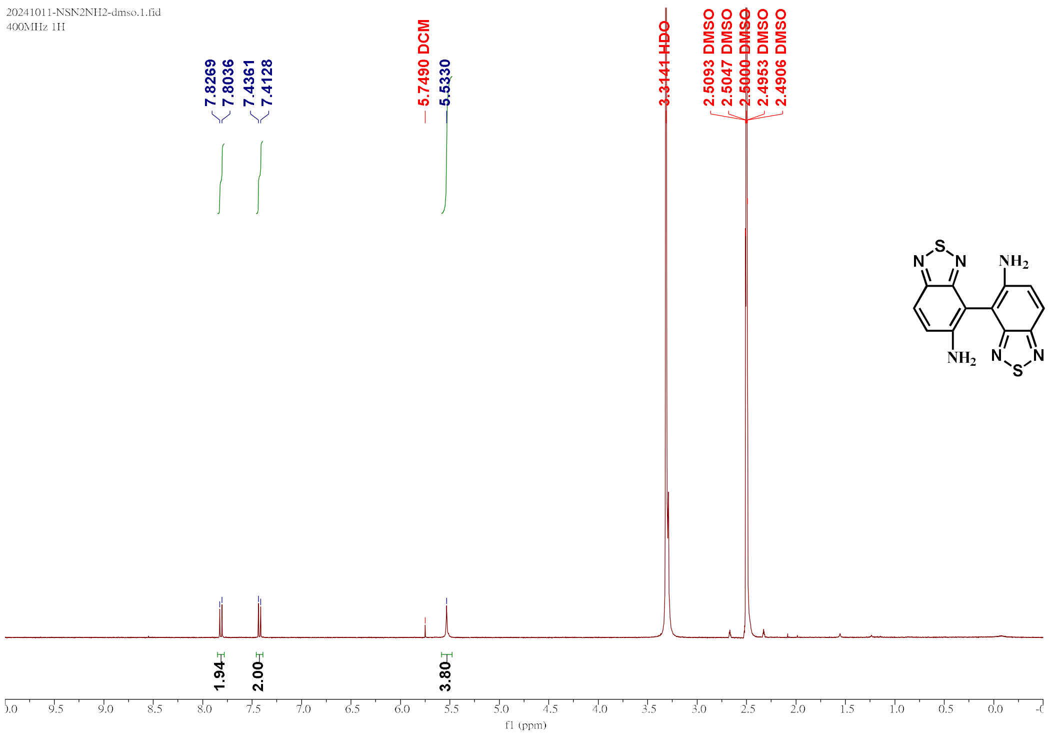

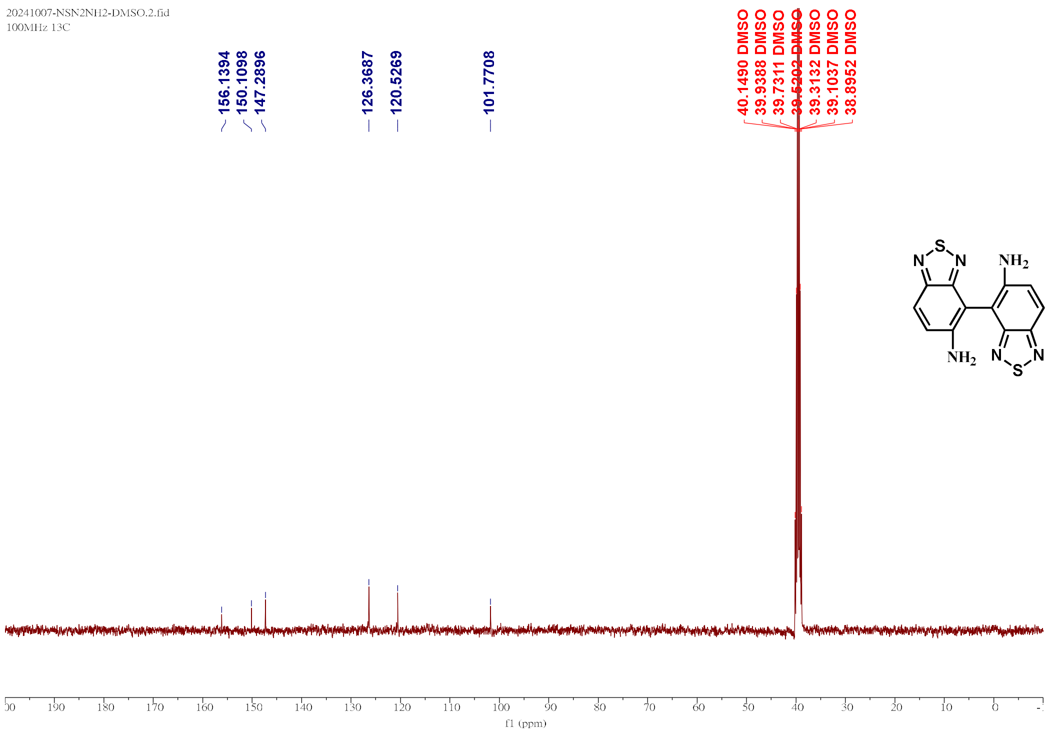

Figure S37. ^{1}H and ^{13}C NMR spectra of compound **3** (DMSO-d_6).

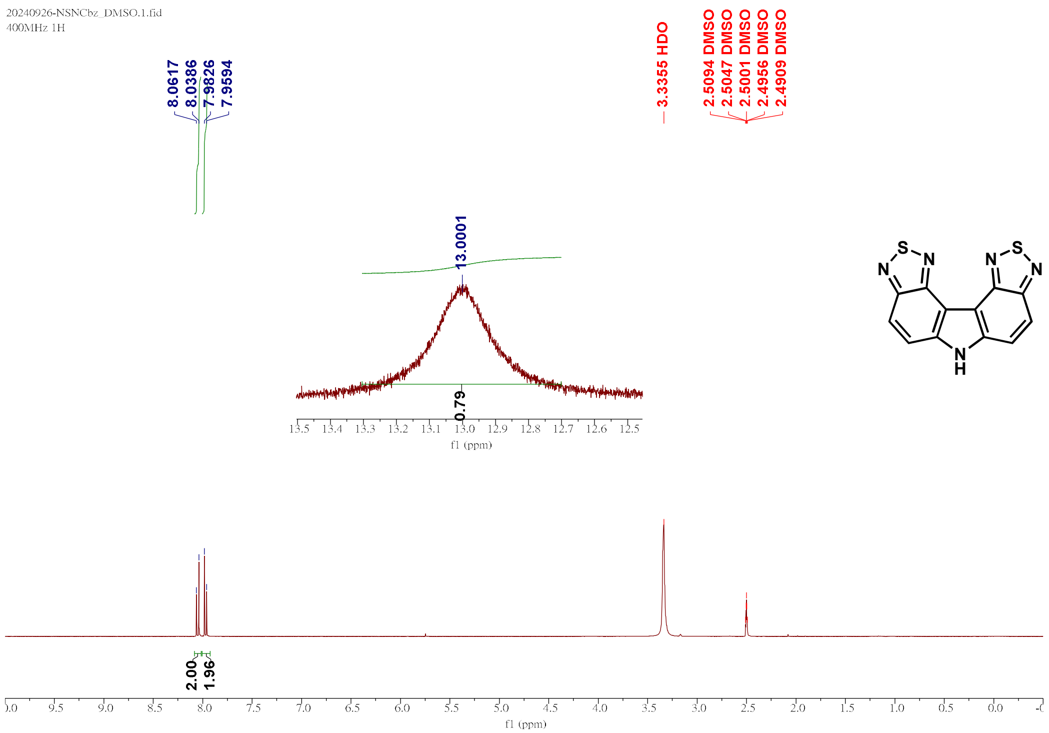

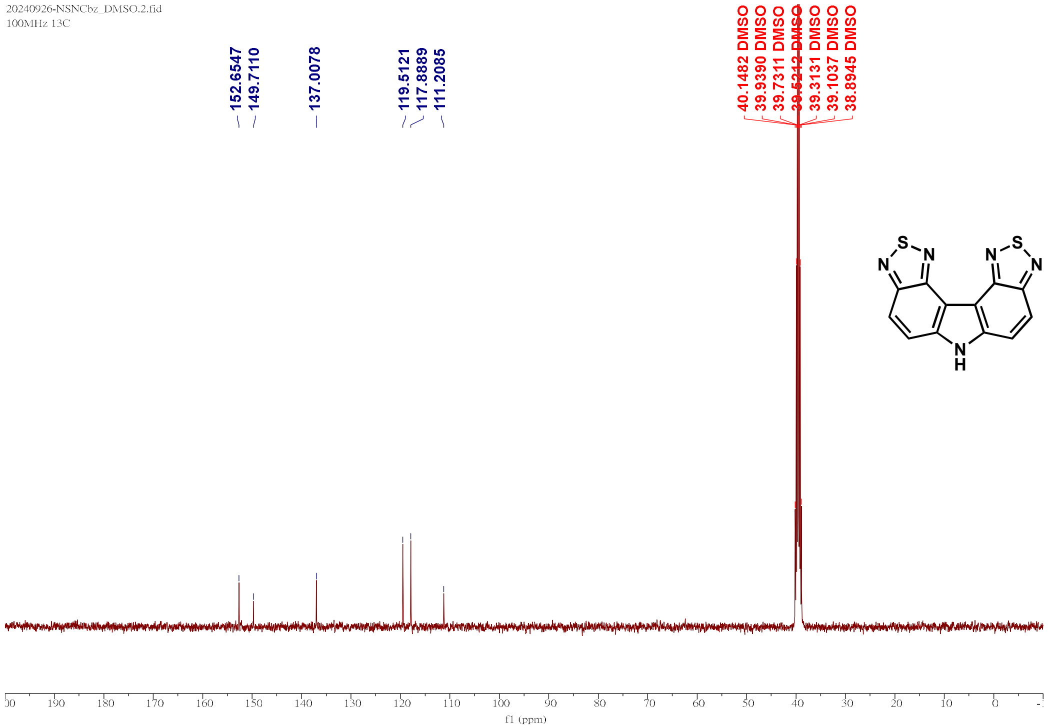

Figure S38. ^{1}H and ^{13}C NMR spectra of compound **4** (DMSO-d_6).

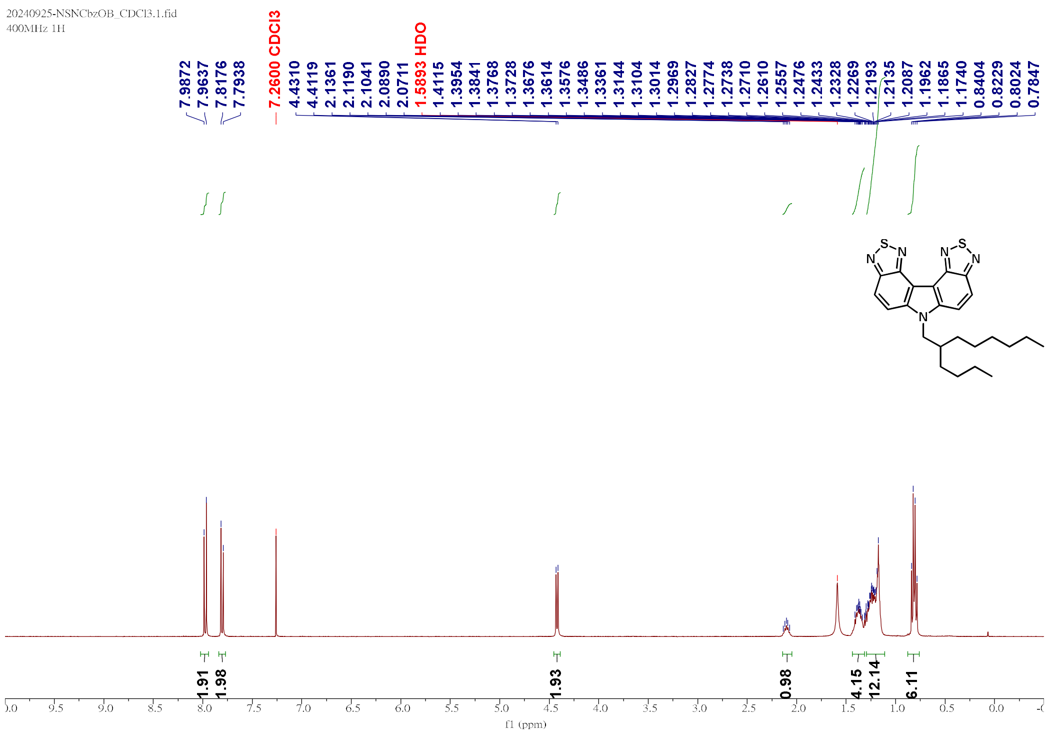

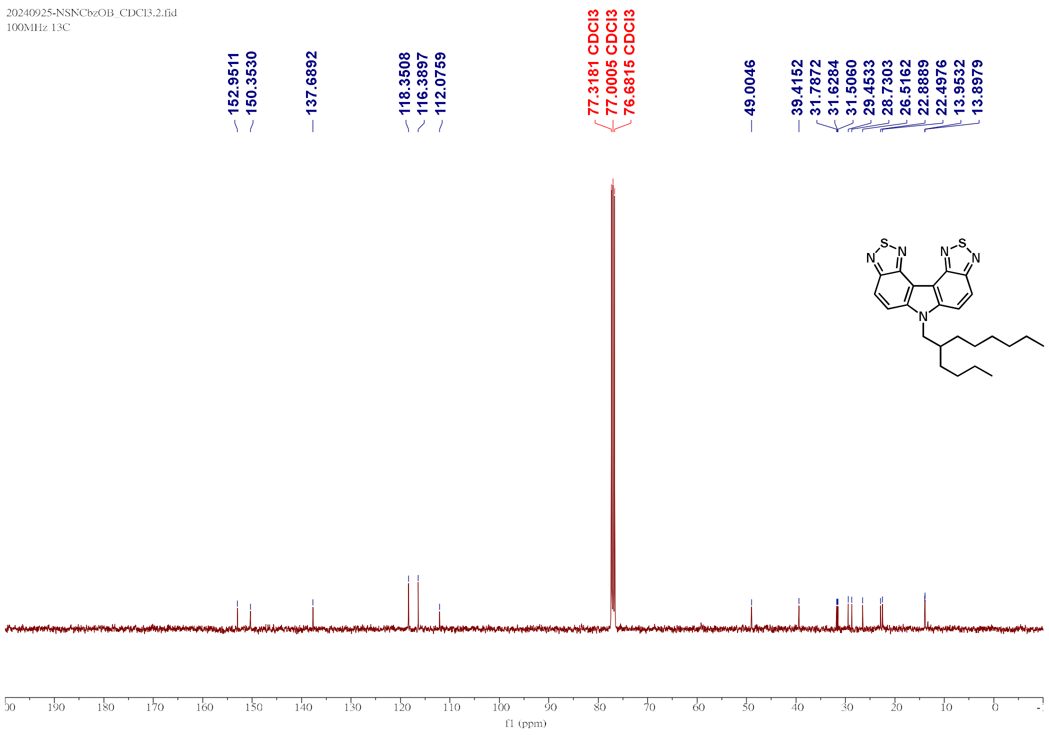

Figure S39. ^{1}H and ^{13}C NMR spectra of **NSN-BO** ($CDCl_3$).

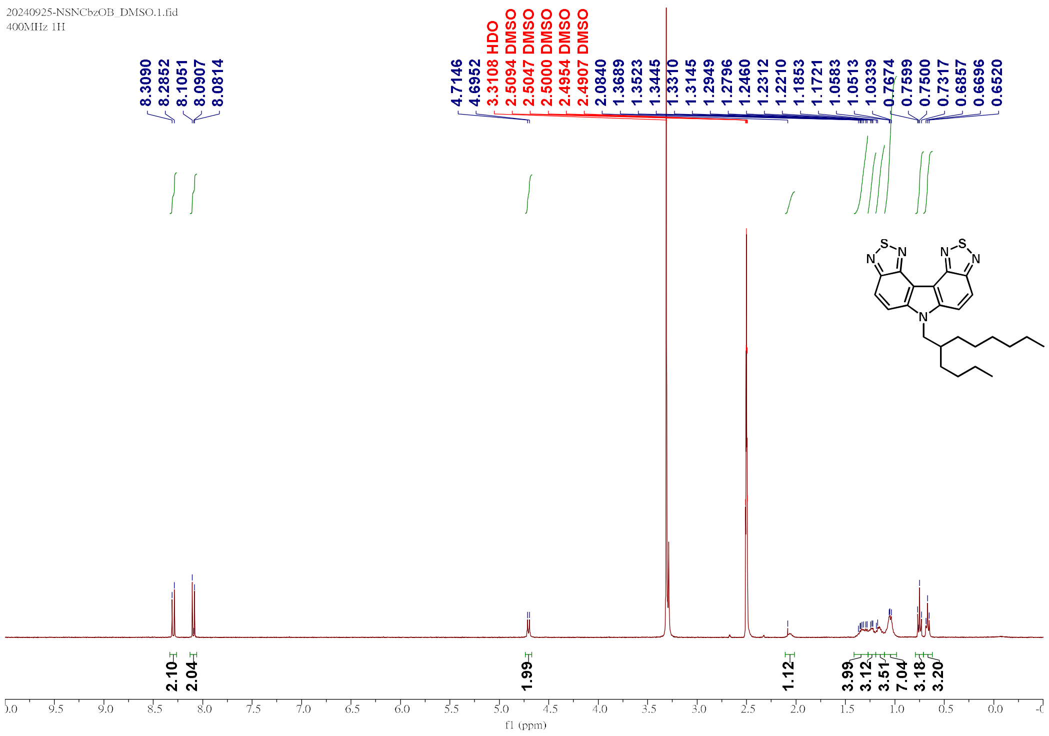

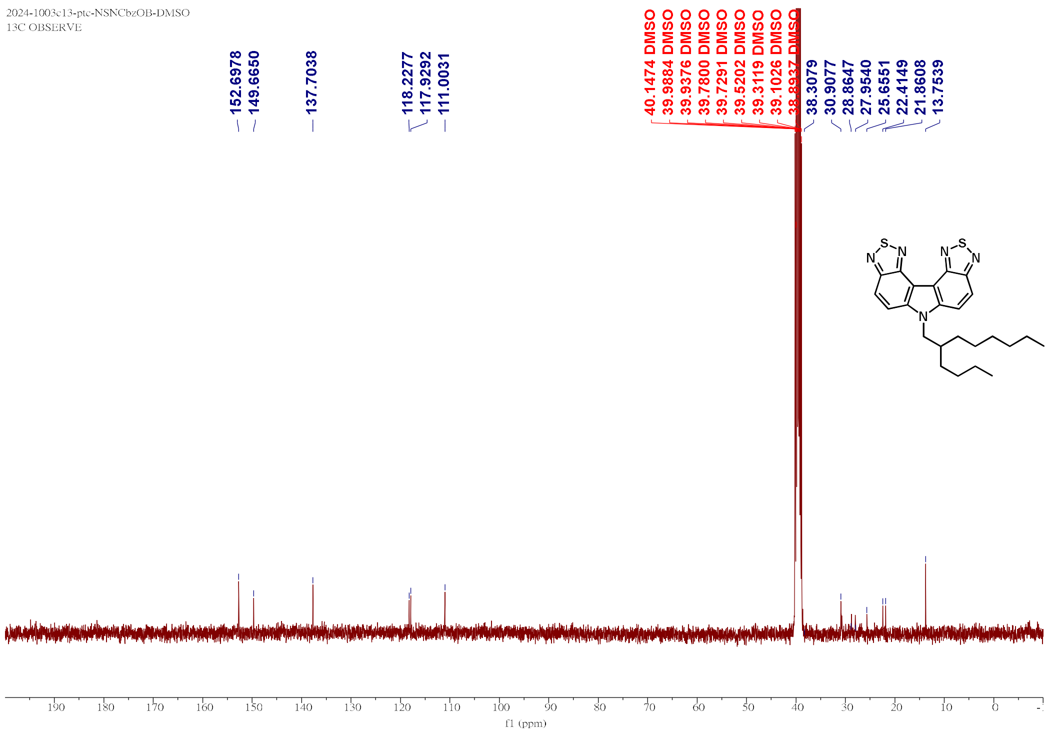

Figure S40. ^{1}H and ^{13}C NMR spectra of **NSN-BO** (DMSO-d_6).

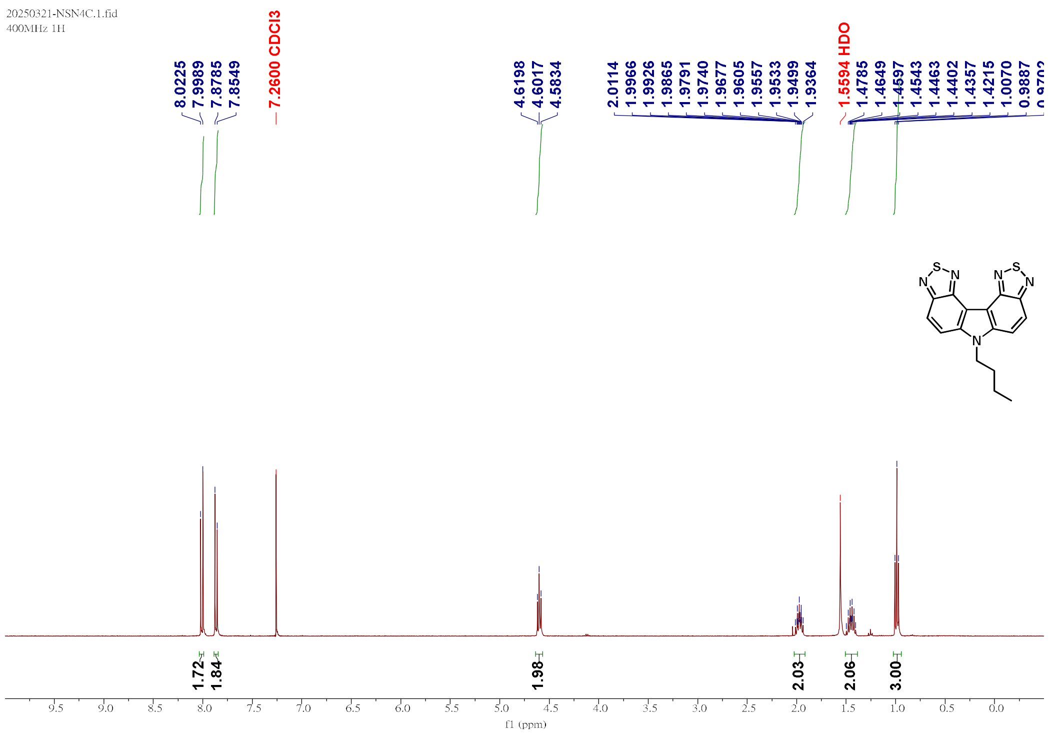

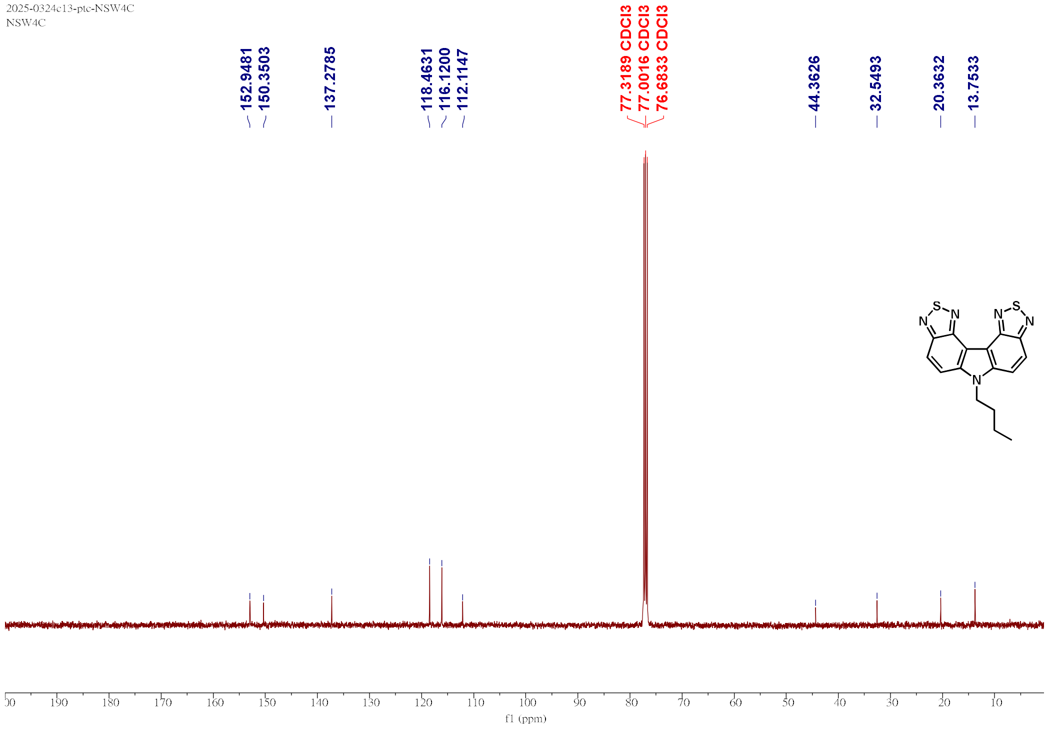

Figure S41. ^{1}H and ^{13}C NMR spectra of **NSN-C4** ($CDCl_3$).

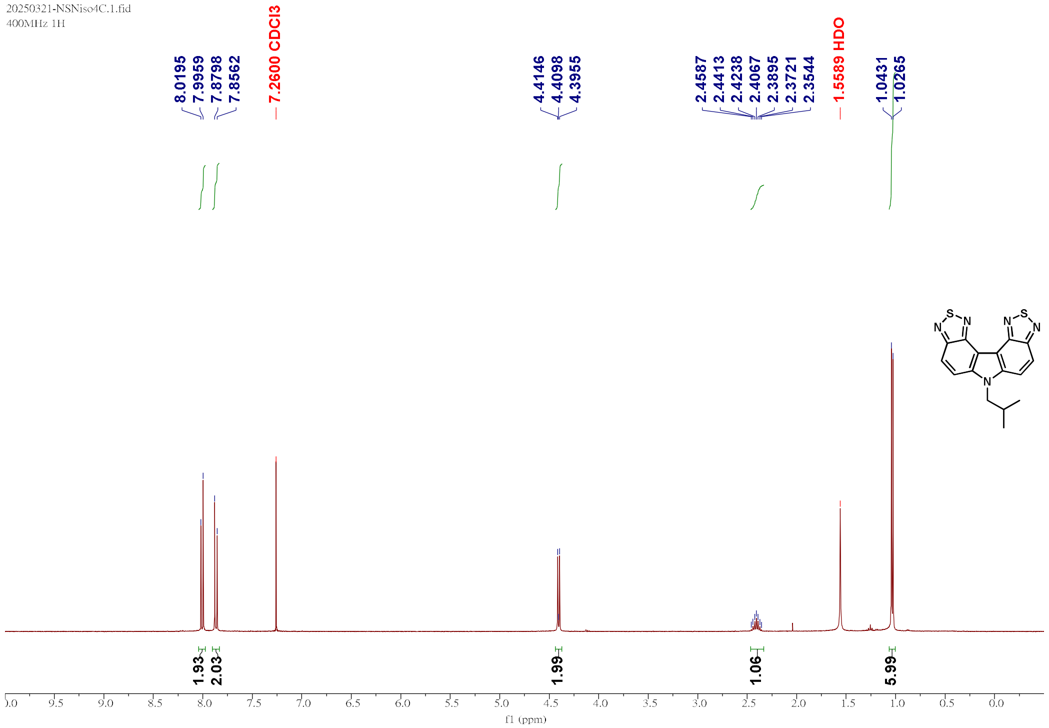

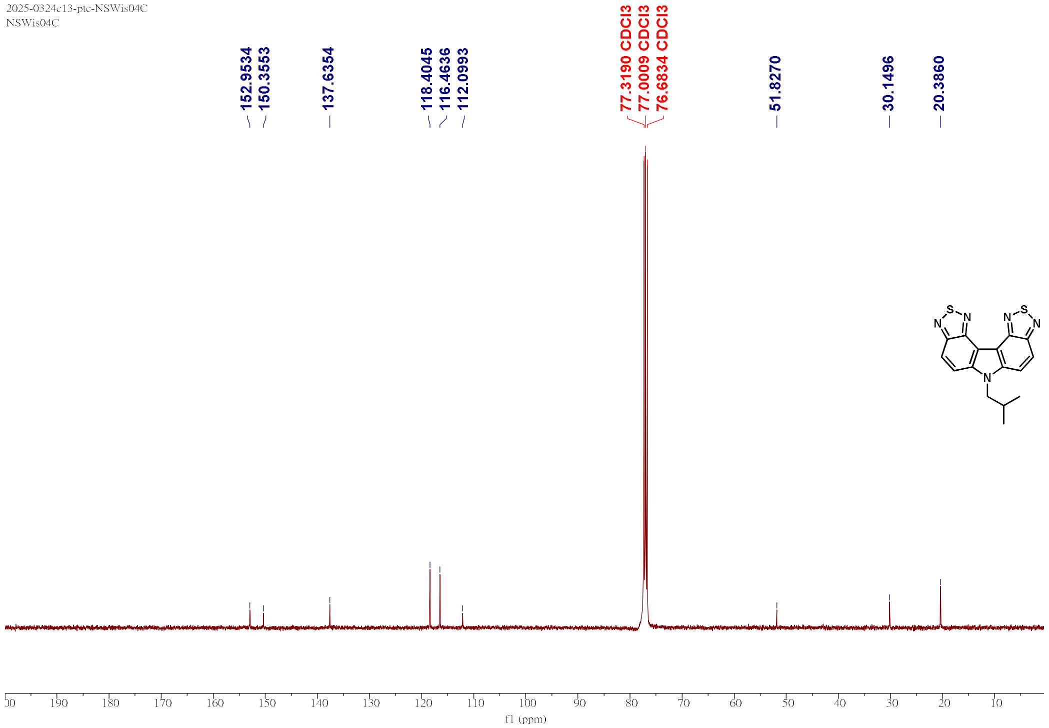

Figure S42. ^{1}H and ^{13}C NMR spectra of **NSN-IB** ($CDCl_3$).

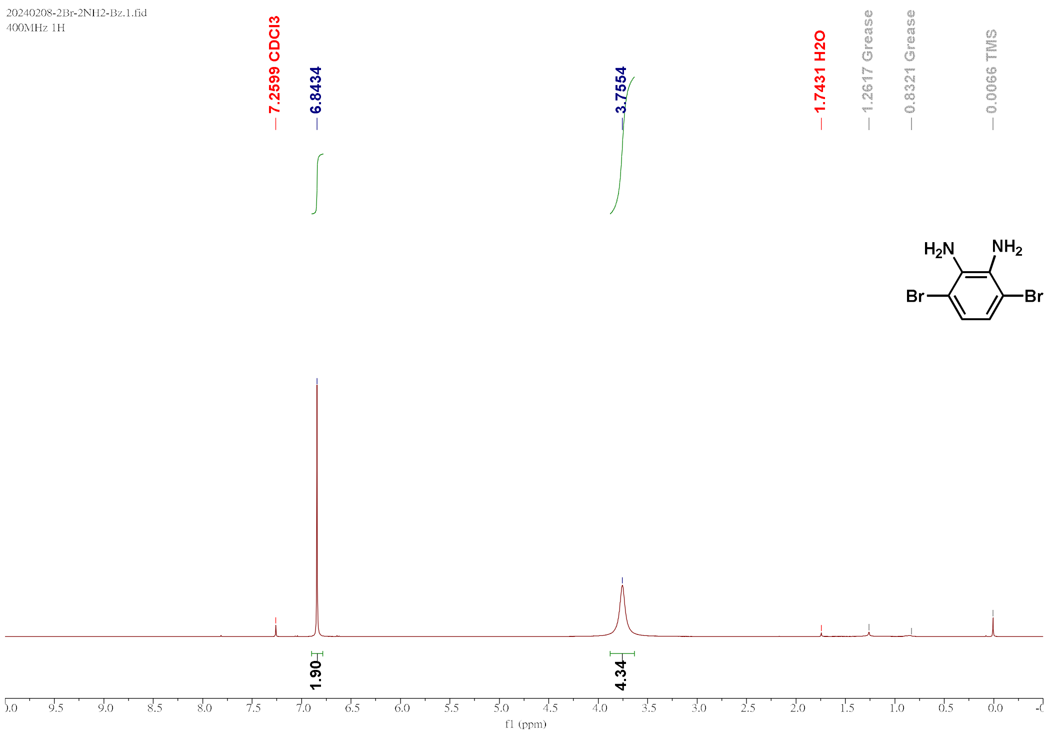

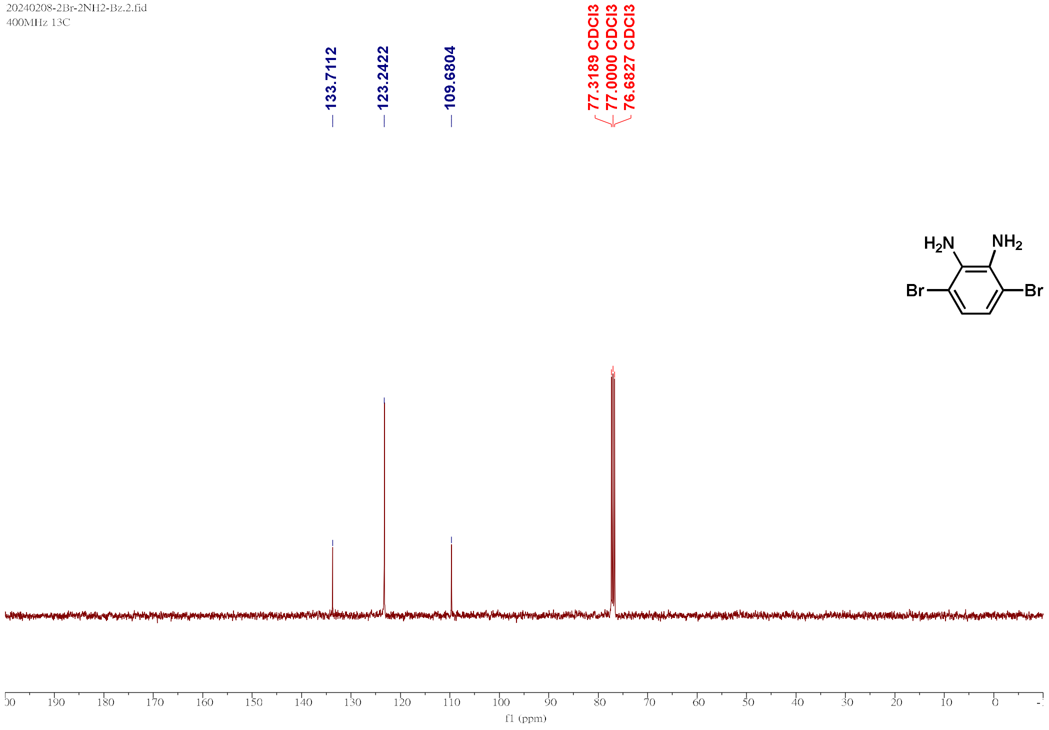

Figure S43. ^{1}H and ^{13}C NMR spectra of compound **5** ($CDCl_3$).

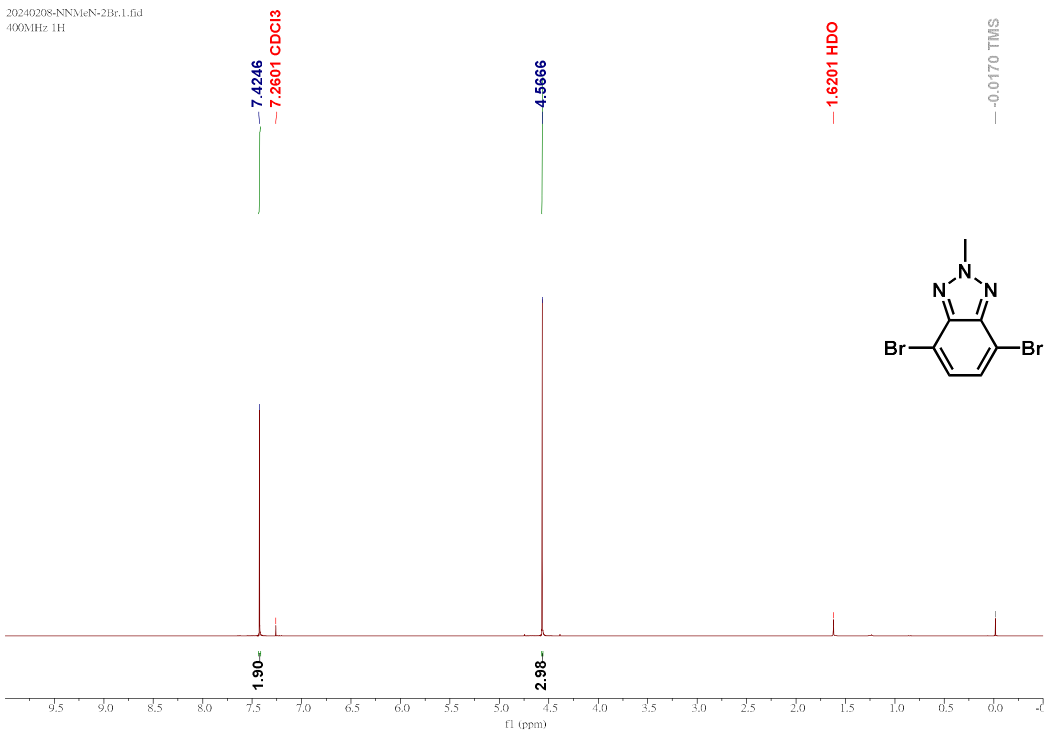

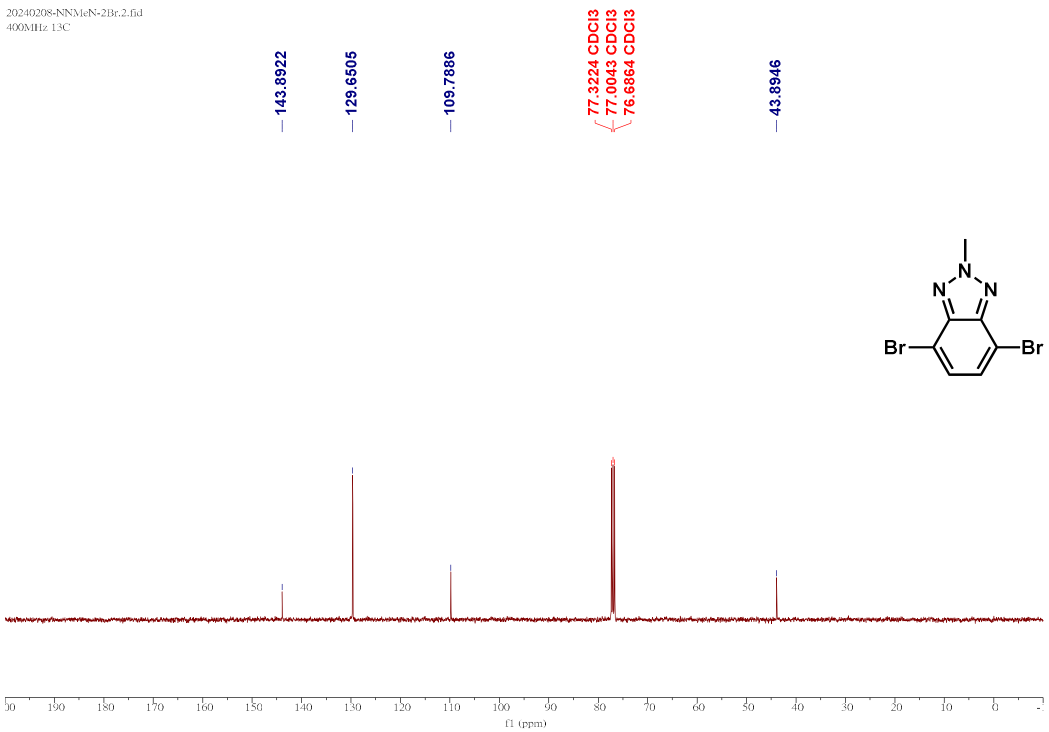

Figure S44. ^{1}H and ^{13}C NMR spectra of compound **6** ($CDCl_3$).

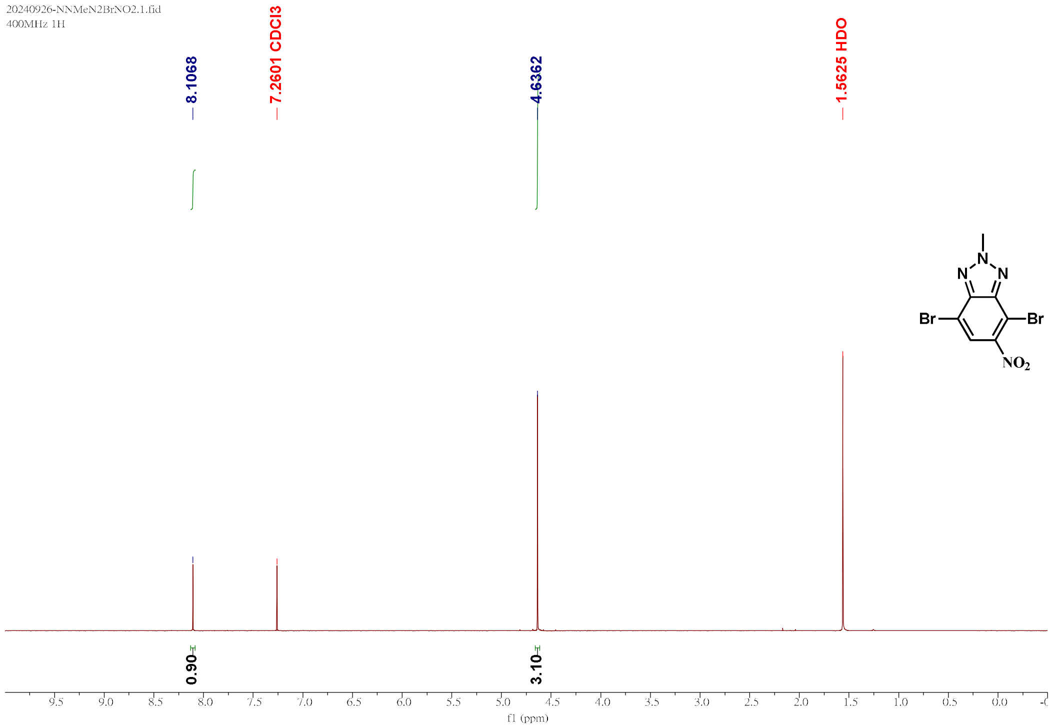

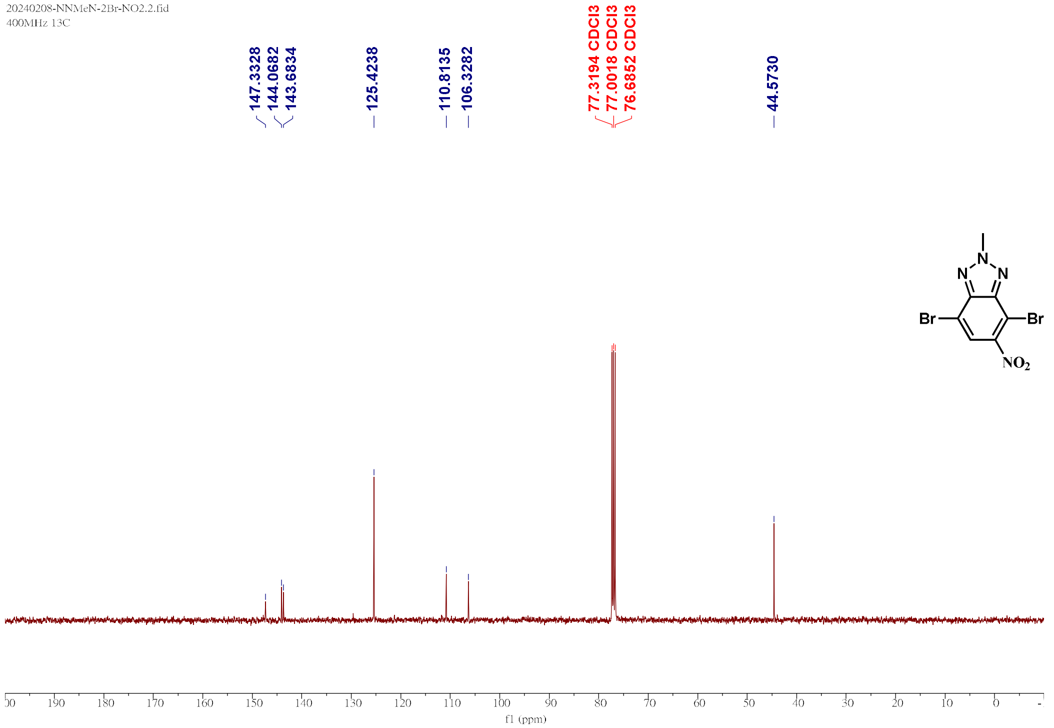

Figure S45. ^{1}H and ^{13}C NMR spectra of compound **7** ($CDCl_3$).

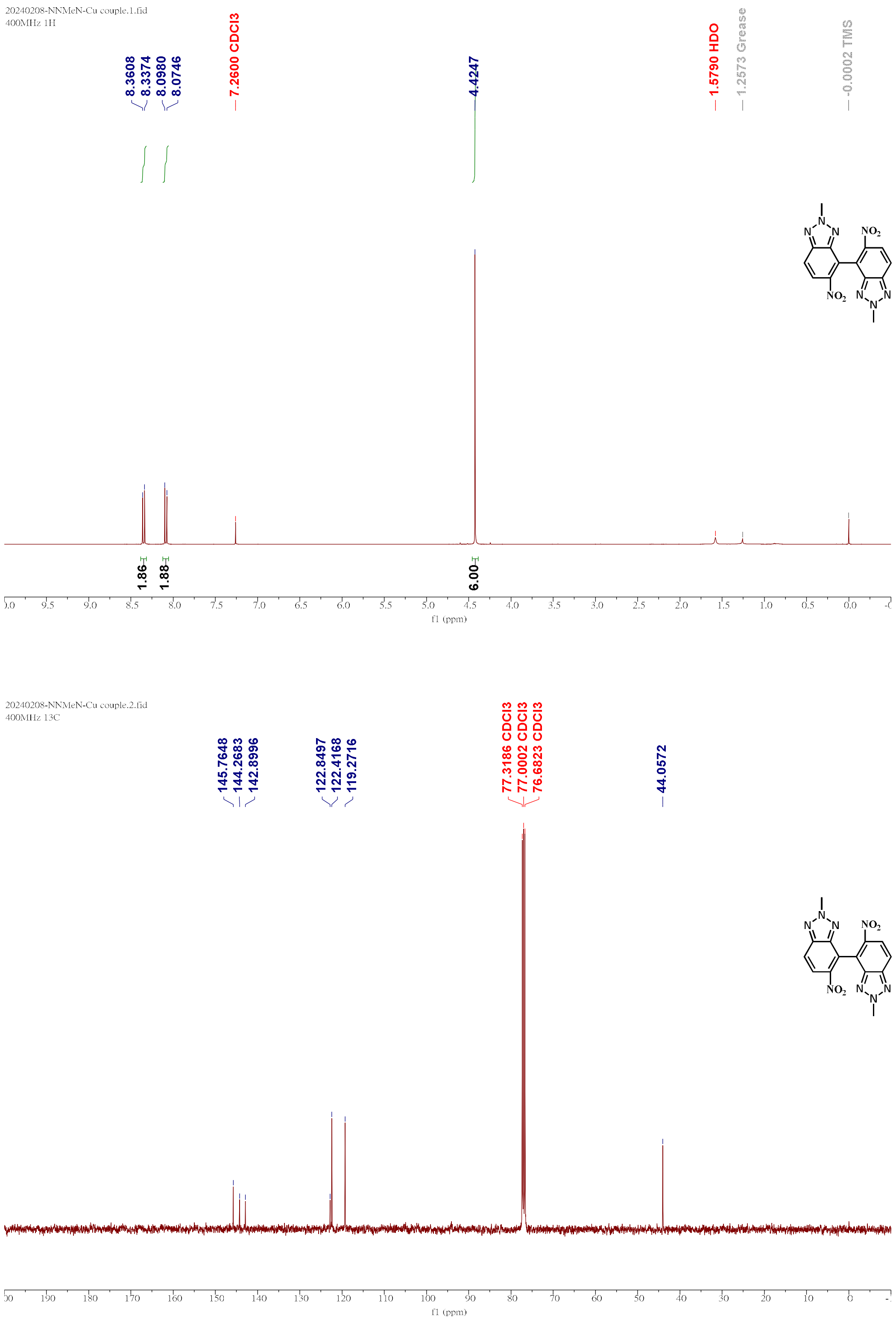

Figure S46. ^{1}H and ^{13}C NMR spectra of compound **8** ($CDCl_3$).

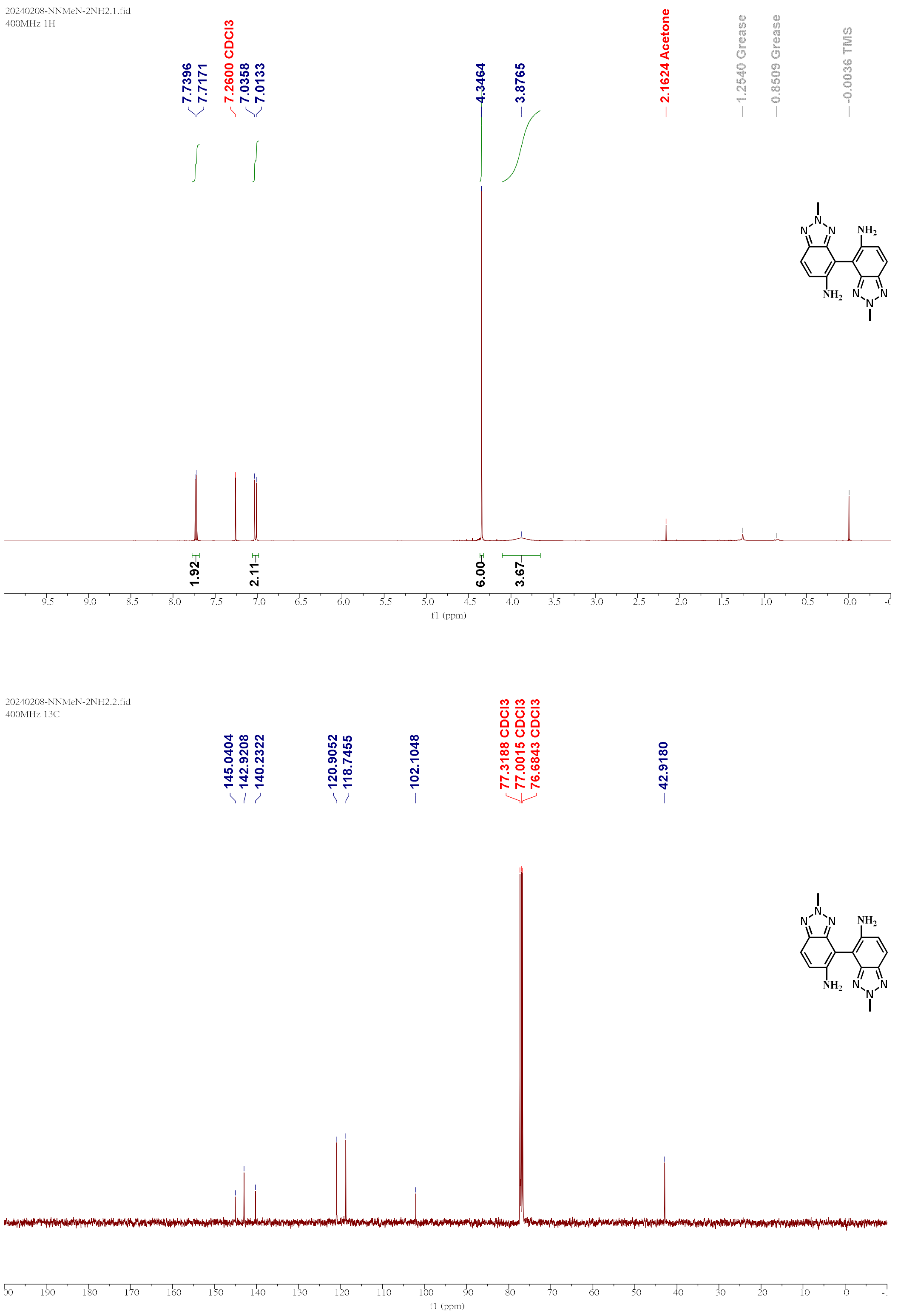

Figure S47. ^{1}H and ^{13}C NMR spectra of compound **9** ($CDCl_3$).

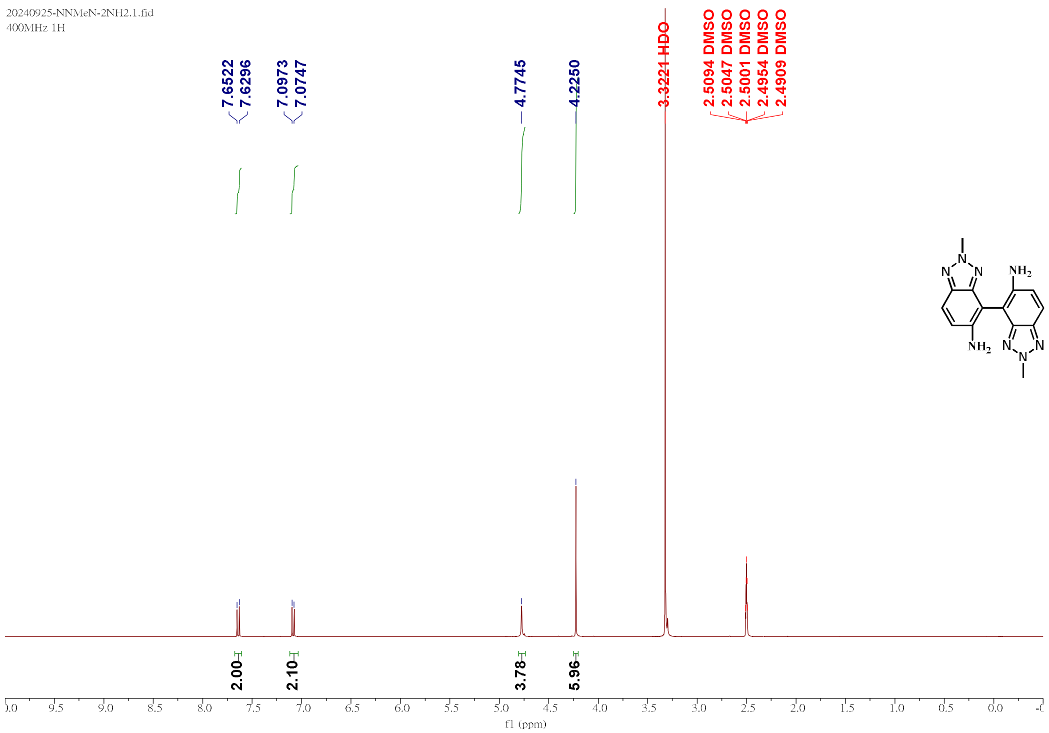

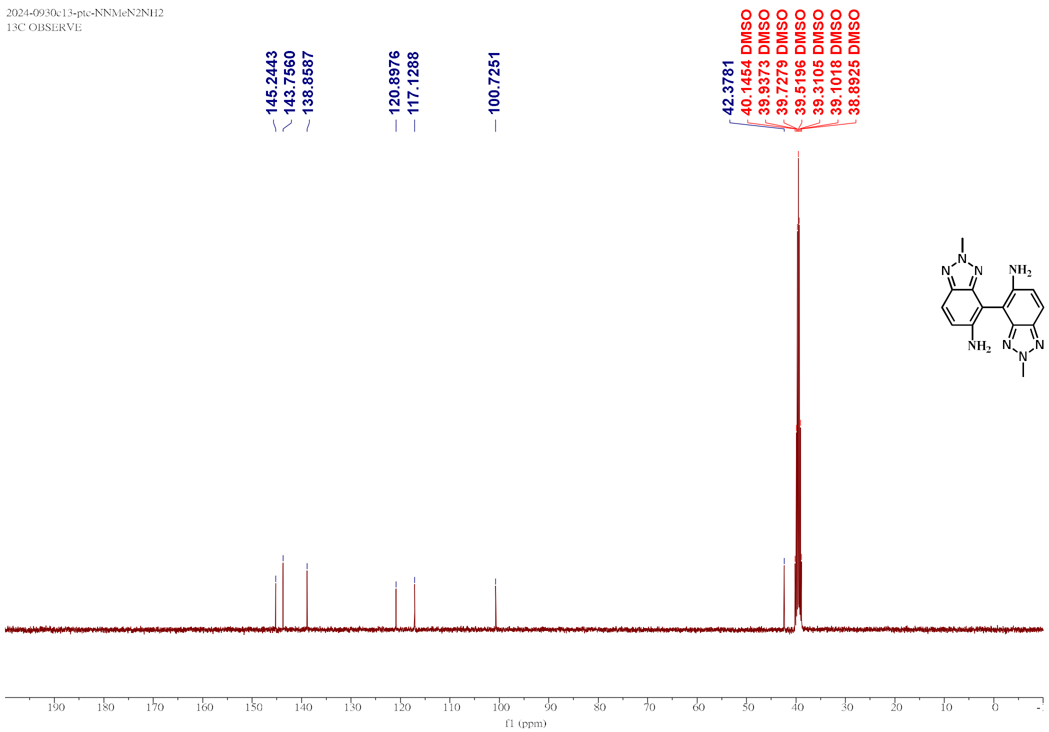

Figure S48. ^{1}H and ^{13}C NMR spectra of compound **9** (DMSO-d_6).

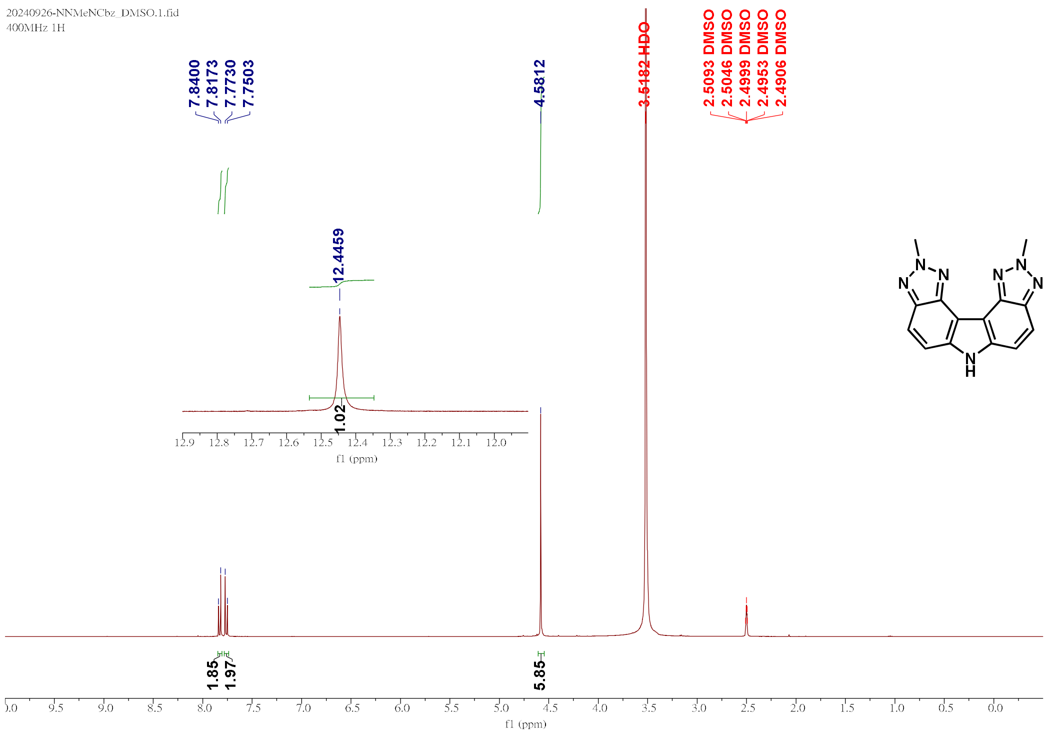

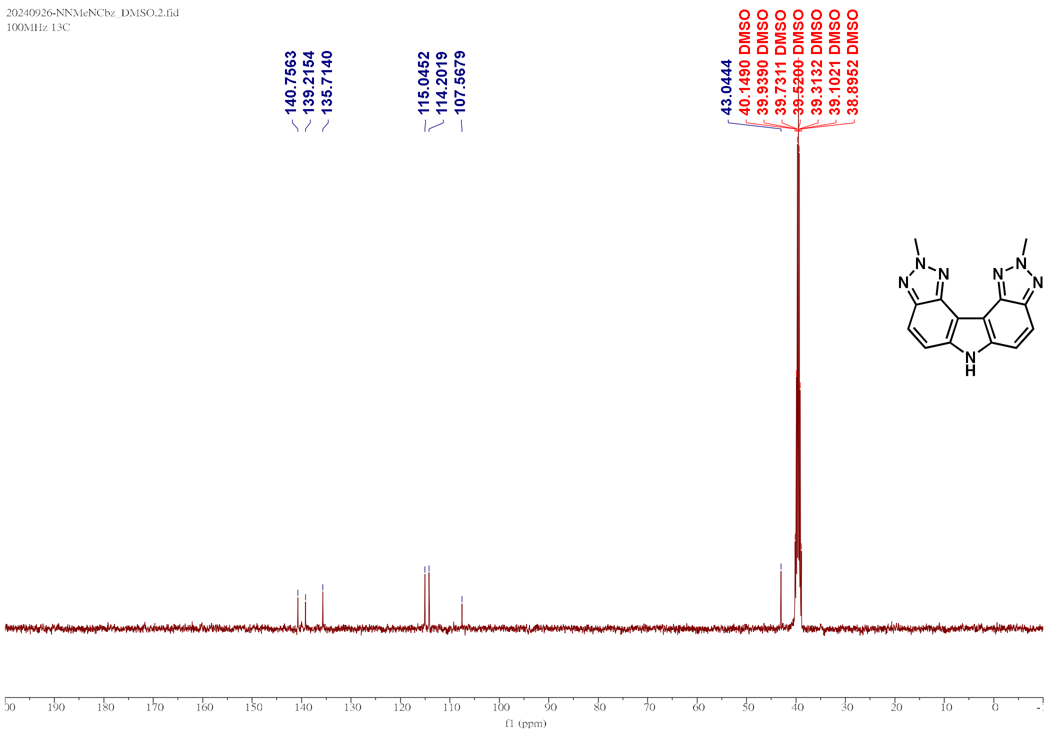

Figure S49. ^{1}H and ^{13}C NMR spectra of compound **10** (DMSO-d_6).

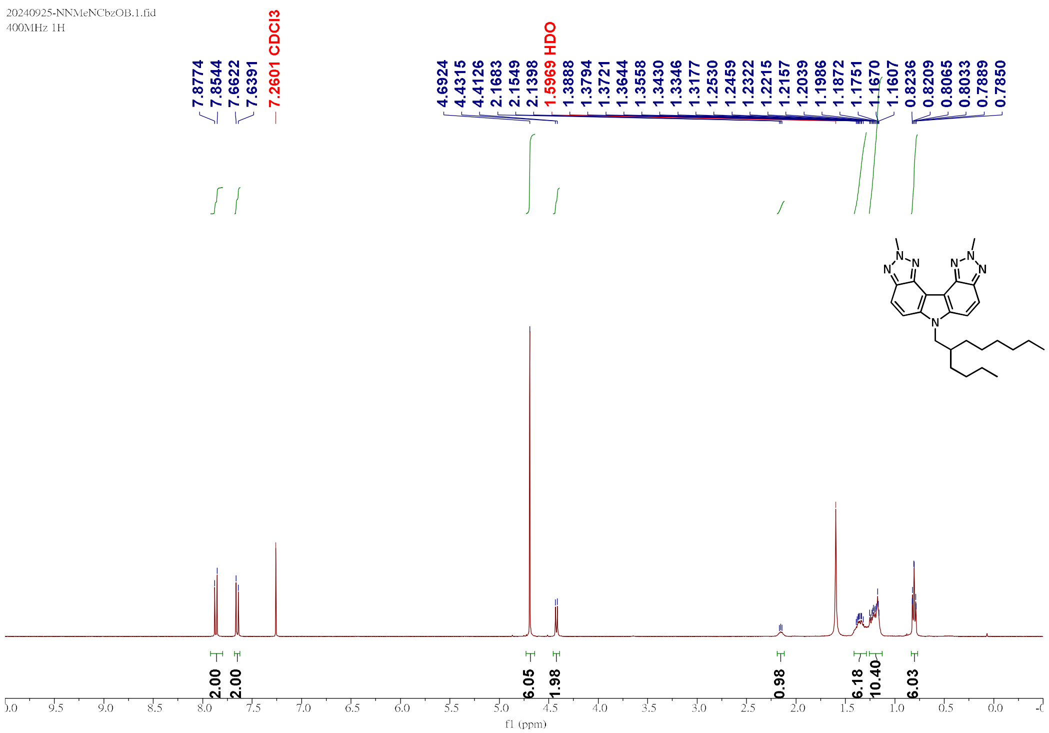

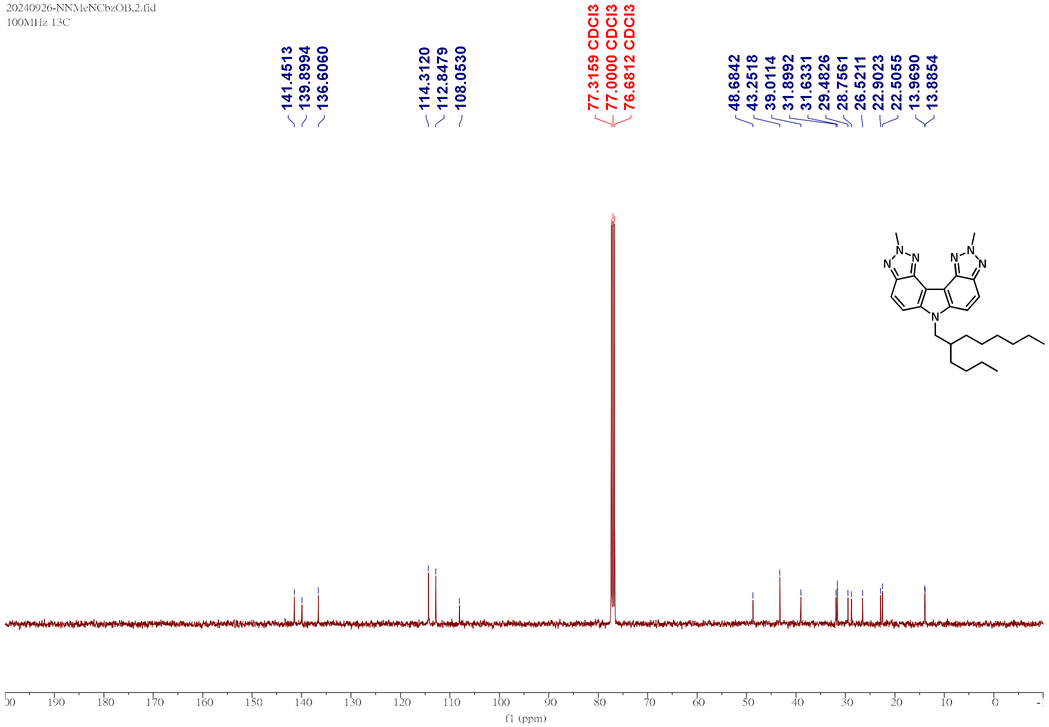

Figure S50. ^{1}H and ^{13}C NMR spectra of **NNN-BO** ($CDCl_3$).

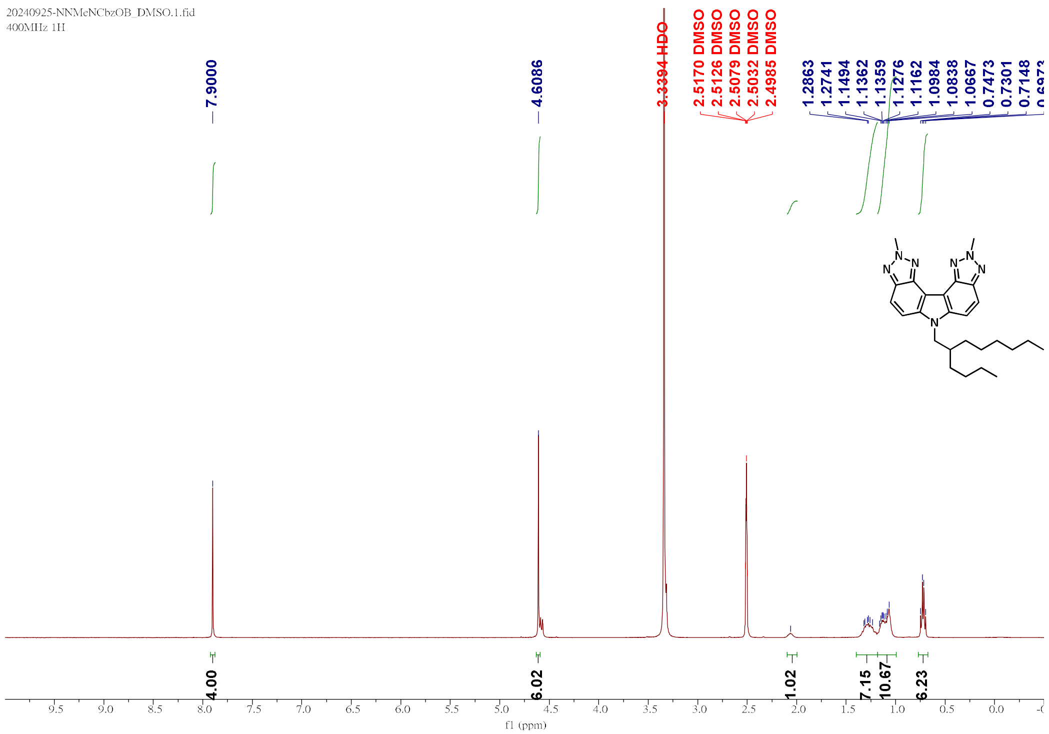

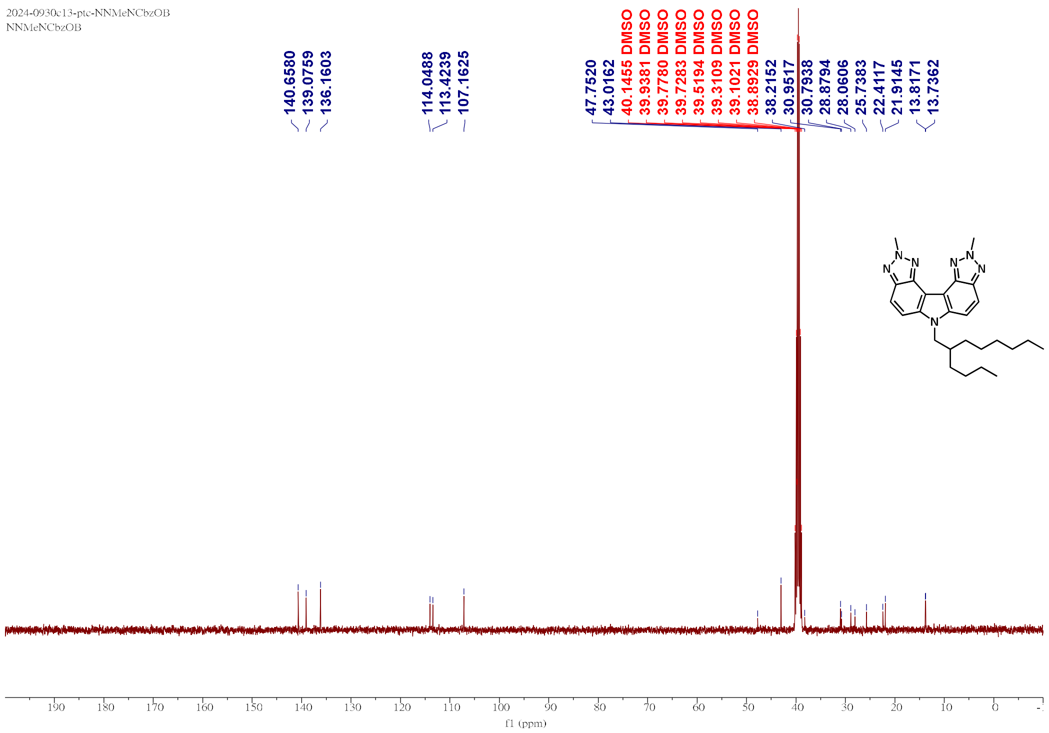

Figure S51. ^{1}H and ^{13}C NMR spectra of **NNN-BO** (DMSO-d_6).

X-ray Crystallography

Single crystals of titled compounds were achieved from slow evaporation of dichloromethane/hexane solution at room temperature. Single crystal X-ray diffraction data were obtained from a Bruker D8 VENTURE single-crystal XRD equipped with Oxford Cryostream 800+ at a temperature of 200 K. Structures of the crystals were solved by direct methods using the SHELXS-97 software.

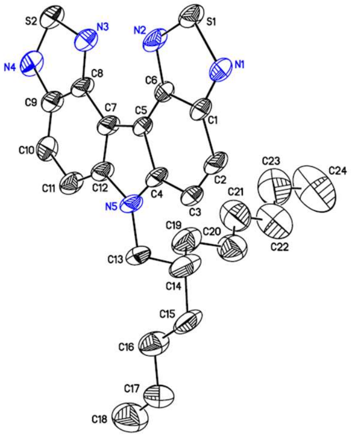

Figure S52. The crystal structure of **NSN-BO**.

Table S2. Crystal data and experimental details for **NSN-BO**.

**CCDC NO**	2421112	
**Empirical formula**	C24 H29 N5 S2	
**Formula weight**	451.64	
**Crystal system**	Monoclinic	
**Space group**	$P2_1/c$	
**Unit cell dimensions**	a = 22.146(3) Å	α= 90°.
	b = 10.7147(9) Å	β= 102.651(6)°.
	c = 10.0561(11) Å	γ = 90°.
**Volume**	2328.2(4) $Å^3$	
**Z**	4	
**F(000)**	960	
**Density (calculated)**	1.288 Mg/m^3	
**Wavelength**	1.54178 Å	
**Cell parameters reflections used**	6774	
**Theta range for Cell parameters**	4.09 to 69.73°.	
**Absorption coefficient**	2.230 mm^{-1}	
**Temperature**	100(2) K	
**Crystal size**	0.100 x 0.050 x 0.020 mm^3	
**Data collection**		
**Diffractometer**	Bruker AXS D8 VENTURE, PhotonIII_C28	

**Absorption correction**	Semi-empirical from equivalents
**Max. and min. transmission**	1.0000 and 0.4259
**No. of measured reflections**	15789
**No. of independent reflections**	4228 [R(int) = 0.1499]
**No. of observed [I>2_igma(I)]**	2470
**Completeness to theta = 67.679°**	99.8 %
**Theta range for data collection**	2.045 to 67.999°.
**Refinement**	
**Final R indices [I>2sigma(I)]**	R1 = 0.1298, wR2 = 0.2992
**R indices (all data)**	R1 = 0.1812, wR2 = 0.3279
**Goodness-of-fit on F2**	1.499
**No. of reflections**	4228
**No. of parameters**	283
**No. of restraints**	3
**Largest diff. peak and hole**	0.908 and -0.404 e.Å^{-3}

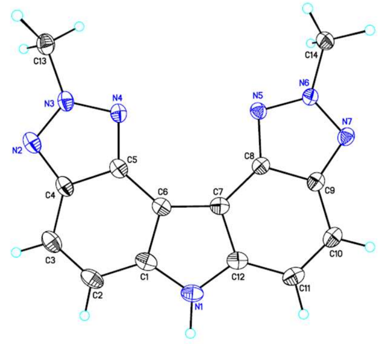

Figure S53. The crystal structure of compound **10**.

Table S3. Crystal data and experimental details for compound **10**.

**CCDC NO**	2421114	
**Empirical formula**	C14 H13 N7 O	
**Formula weight**	295.31	
**Crystal system**	Monoclinic	
**Space group**	$P2_1/c$	
**Unit cell dimensions**	a = 19.8504(6) Å	α= 90°.
	b = 3.91550(10) Å	β= 98.4764(10)°.
	c = 17.1158(5) Å	γ = 90°.
**Volume**	1315.78(6) Å^3	

**Z**	4
**F(000)**	616
**Density (calculated)**	1.491 Mg/m^3
**Wavelength**	1.54178 Å
**Cell parameters reflections used**	9943
**Theta range for Cell parameters**	2.25 to 70.15°.
**Absorption coefficient**	0.846 mm^{-1}
**Temperature**	100(2) K
**Crystal size**	0.080 x 0.050 x 0.050 mm^3
**Data collection**	
**Diffractometer**	Bruker AXS D8 VENTURE, PhotonIII_C28
**Absorption correction**	Semi-empirical from equivalents
**Max. and min. transmission**	1.0000 and 0.9005
**No. of measured reflections**	17569
**No. of independent reflections**	2474 [R(int) = 0.0354]
**No. of observed [I>2_igma(I)]**	2381
**Completeness to theta = 67.679°**	99.4 %
**Theta range for data collection**	2.250 to 70.142°.
**Refinement**	
**Final R indices [I>2sigma(I)]**	R1 = 0.0401, wR2 = 0.1073
**R indices (all data)**	R1 = 0.0410, wR2 = 0.1081
**Goodness-of-fit on F^2**	0.989
**No. of reflections**	2474
**No. of parameters**	213
**No. of restraints**	0
**Largest diff. peak and hole**	0.825 and -0.255 e.Å^{-3}

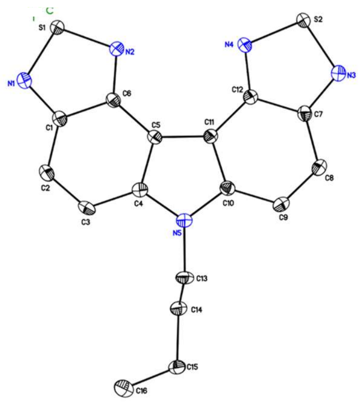

Figure S54. The crystal structure of **NSN-C4**.

Table S4. Crystal data and experimental details for **NSN-C4**.

**CCDC NO**	2434210	
**Empirical formula**	C16 H13 N5 S2	
**Formula weight**	339.43	
**Crystal system**	Monoclinic	
**Space group**	$P2_1/c$	
**Unit cell dimensions**	a = 12.4940(5) Å<br>b = 13.9096(5) Å<br>c = 9.0080(3) Å	α= 90°.<br>β= 110.4578(10)°.<br>γ = 90°.
**Volume**	1466.74(9) Å^3	
**Z**	4	
**F(000)**	704	
**Density (calculated)**	1.537 Mg/m^3	
**Wavelength**	1.54178 Å	
**Cell parameters reflections used**	9920	
**Theta range for Cell parameters**	4.94 to 70.09°.	
**Absorption coefficient**	3.341 mm^{-1}	
**Temperature**	100(2) K	
**Crystal size**	0.300 x 0.250 x 0.100 mm^3	
**Data collection**		
**Diffractometer**	Bruker D8 VENTURE, PhotonIII_C28	
**Absorption correction**	Semi-empirical from equivalents	
**Max. and min. transmission**	1.0000 and 0.7146	
**No. of measured reflections**	26145	
**No. of independent reflections**	2776 [R(int) = 0.0277]	
**No. of observed [I>2_igma(I)]**	2720	
**Completeness to theta = 67.679°**	99.8 %	
**Theta range for data collection**	3.776 to 70.143°.	
**Refinement**		
**Final R indices [I>2sigma(I)]**	R1 = 0.0294, wR2 = 0.0819	
**R indices (all data)**	R1 = 0.0298, wR2 = 0.0823	
**Goodness-of-fit on F^2**	1.058	
**No. of reflections**	2776	
**No. of parameters**	208	
**No. of restraints**	0	
**Largest diff. peak and hole**	0.214 and -0.492 e.Å^{-3}	

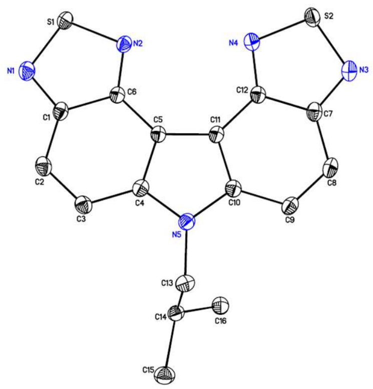

Figure S55. The crystal structure of **NSN-IB**.

Table S5. Crystal data and experimental details for **NSN-IB**.

**CCDC NO**	2434211
**Empirical formula**	C16 H13 N5 S2
**Formula weight**	339.43
**Crystal system**	Monoclinic
**Space group**	$P2_1/c$
**Unit cell dimensions**	a = 7.5600(2) Å α= 90°.<br>b = 10.2306(3) Å β= 100.0198(10)°.<br>c = 19.3737(6) Å γ = 90°.
**Volume**	1475.57(7) $Å^3$
**Z**	4
**F(000)**	704
**Density (calculated)**	1.528 Mg/m^3
**Wavelength**	1.54178 Å
**Cell parameters reflections used**	9144
**Theta range for Cell parameters**	4.64 to 70.15°.
**Absorption coefficient**	3.321 mm^{-1}
**Temperature**	100(2) K
**Crystal size**	0.200 x 0.150 x 0.100 mm^3
**Data collection**	
**Diffractometer**	Bruker D8 VENTURE, PhotonIII_C28
**Absorption correction**	Semi-empirical from equivalents
**Max. and min. transmission**	1.0000 and 0.7781
**No. of measured reflections**	23691
**No. of independent reflections**	2805 [R(int) = 0.0291]
**No. of observed [I>2_igma(I)]**	2730
**Completeness to theta = 67.679°**	99.9 %

**Theta range for data collection**	4.635 to 70.139°.
**Refinement**	
**Final R indices [I>2sigma(I)]**	R1 = 0.0294, wR2 = 0.0845
**R indices (all data)**	R1 = 0.0317, wR2 = 0.0947
**Goodness-of-fit on F^2**	1.141
**No. of reflections**	2805
**No. of parameters**	210
**No. of restraints**	0
**Largest diff. peak and hole**	0.298 and -0.471 e.$Å^{-3}$

References:

(1) Wei, Y.-C.; Chen, B.-H.; Ye, R.-S.; Huang, H.-W.; Su, J.-X.; Lin, C.-Y.; Hodgkiss, J.; Hsu, L.-Y.; Chi, Y.; Chen, K.; Lu, C.-H.; Yang, S.-D.; Chou, P.-T. Excited-State THz Vibrations in Aggregates of PtII Complexes Contribute to the Enhancement of Near-Infrared Emission Efficiencies. *Angew. Chem. Int. Ed.* **2023**, *62* (16), e202300815.

(2) Lu, C.-H.; Tsou, Y.-J.; Chen, H.-Y.; Chen, B.-H.; Cheng, Y.-C.; Yang, S.-D.; Chen, M.-C.; Hsu, C.-C.; Kung, A. H. Generation of intense supercontinuum in condensed media. *Optica* **2014**, *1* (6), 400-406.

(3) Lu, C.-H.; Wu, W.-H.; Kuo, S.-H.; Guo, J.-Y.; Chen, M.-C.; Yang, S.-D.; Kung, A. H. Greater than 50 times compression of 1030 nm Yb:KGW laser pulses to single-cycle duration. *Opt. Express* **2019**, *27* (11), 15638-15648.
